# Indazol-Pyrimidine Hybrids: Design, Synthesis, and Antiproliferative Activity Against Human Cancer Cell Lines

**DOI:** 10.3390/molecules30183773

**Published:** 2025-09-17

**Authors:** Hanaa M. Al-Tuwaijri, Ahmed A. El-Rashedy, Siddique Akber Ansari, Aliyah Almomen, Hamad M. Alkahtani, Ebtehal S. Al-Abdullah, Mogedda E. Haiba

**Affiliations:** 1 Department of Pharmaceutical Chemistry, College of Pharmacy, King Saud University, P.O. Box 2457, Riyadh 11451, Saudi Arabia; haltuwajri@gmail.com (H.M.A.-T.); ansariakber@gmail.com (S.A.A.); alalmomen@ksu.edu.sa (A.A.); ahamed@ksu.edu.sa (H.M.A.); 2 Department of Natural and Microbial Products National Research Center, El Buhouth Street, Dokki, Cairo 12622, Egypt; ahmedelrashedy45@gmail.com; 3 Department Organic and Medicinal Chemistry, Faculty of Pharmacy, University of Sadat City, Menoufia 32897, Egypt; 4 Department of Therapeutic Chemistry, Pharmaceutical and Drug Industries Research Division, National Research Center, El Buhouth Street, Dokki, Cairo 12622, Egypt

**Keywords:** indazol-pyrimidine derivatives, cytotoxic activity, molecular docking, molecular dynamics

## Abstract

The current study outlines a synthetic method for creating a new class of indazol-pyrimidine derivatives **4a**–**h** and **5a**–**h**. The new derivatives were evaluated as in vitro cytotoxic agents against three types of cancer cell lines (MCF-7, A549 and Caco-2), utilizing the MTT assay. Compounds **4a**, **4c**, **4d**, **5a** and **5f** demonstrated potent cytotoxic activity against MCF-7 cell line, showing higher activity than the reference drug Staurosporine. Among the examined compounds, **5f** showed a strong cytotoxic effect against all three tested cancer cells (MCF-7, A549 and Caco-2), with IC_50_ values of 1.858, 3.628 and 1.056 µM, respectively. In comparison, the reference drug exhibited IC_50_ values of 8.029, 7.354 and 4.202 µM respectively, indicating promising anti-proliferative potential of compound **5f**. On the other hand, Compound 4b demonstrated the greatest potency against Caco-2 cell line, with an IC_50_ of 0.827 µM, markedly outperforming reference compound’s IC_50_ of 4.202 µM. Furthermore, compound **5h** revealed significant anti-proliferative activity against A549 cell line, with an IC_50_ value of 1.378 µM, compared to the reference drug, with an IC_50_ value of 7.354 µM. Additionally, the molecular docking study revealed a strong binding affinity of compound **5f** within the binding site of the c-Kit tyrosine kinase protein, and the molecular dynamics study confirmed its stability.

## 1. Introduction

**Figure 1:**
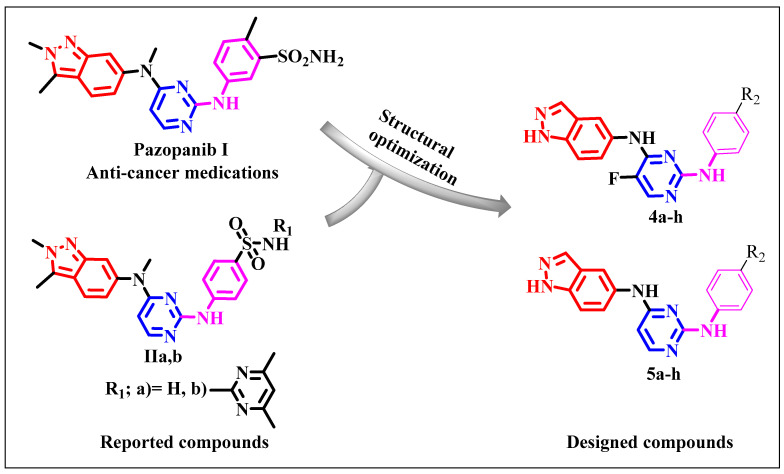
The proposed structures of the new 1*H*-indazol-pyrimidine derivatives.

The word “cancer” is a general phrase that encompasses about 277 distinct cancer disease types. It includes various phases with varying gene alterations that contribute to the development and growth of cancerous cells [1]. Cancer is considered one of the most burdensome diseases leading to death worldwide [2]. According to the estimation of the 2022 cancer mortality rate provided by the World Health Organization (WHO), lung cancer accounted for 12.4%, female breast cancer for 11.6%, and colorectal cancer for 9.6% of all cancer-related deaths [3]. Moreover, the global cancer burden is steadily increasing [4]. Accordingly, researchers from all around the world are attempting to understand the molecular biology of cancer cells in order to develop new and less harmful chemotherapeutic medications with broader cytotoxicity toward tumor cells. Despite advances in cancer treatment, traditional chemotherapeutic specificity drugs still produce significant side effects that can seriously impair healthy human life because chemotherapeutic treatment is ineffective and not only targets cancer cells, but also the normal cells [5]. It was reported that the most widely used medications worldwide are nitrogen-containing heterocycles [6,7]. These heterocyclic backbones solid aromatic structures may be incorporated into the binding pockets and offer a variety of chemical interactions, including non-covalent, hydrophobic, hydrogen, and ionic bonds, which facilitate ligand binding with receptor protein [8,9]. The structurally varied anilinopyrimidine and indazole analogues are an important family of nitrogen-containing heterocycles due to their broader range of biological applications. These have been reported to include antibacterial, anti-inflammatory, anti-tubercular, anti-depressant, and anti-cancer effects [10,11,12,13]. The bicyclic structure of indazole, which consists of both aromatic and indazole moieties, gives it its structural flexibility, which suggests pharmacological effects of indazole compounds on a variety of illnesses [14,15,16,17]. In parallel, pyrimidine derivatives are key components of nucleic acids such as DNA and RNA [18], which led to pyrimidine and its derivatives being accepted for a wide range of medicinal applications, particularly as anticancer [19,20,21,22,23]. One notable example is Pazopanib, a marketed anti-cancer drug that contains both indazole and pyrimidine moieties. It targets tyrosine kinases and has been approved for the treatment of renal cell carcinoma and soft tissue sarcoma [24]. Also it was reported that pazopanib derivatives **IIa** and **IIb** revealed strong action against VEGFR-2 with IC_50_ values of 25 nM and 12 nM, respectively, compared to pazopanib which has an IC_50_ value of 43 nM [25], Figure 1. These observations have motivated us to hybridize indazole and pyrimidine moieties into a single molecule, and to explore a new series of indazole-pyrimidine derivatives, aiming to investigate their anti-proliferative potential against human cell lines, specifically breast cancer (MCF-7), lung cancer (A549), and colorectal cancer (Caco-2), by means of the MTT assay.

## 2. Results and Discussion

### 2.1. Chemistry

**Scheme 1:**
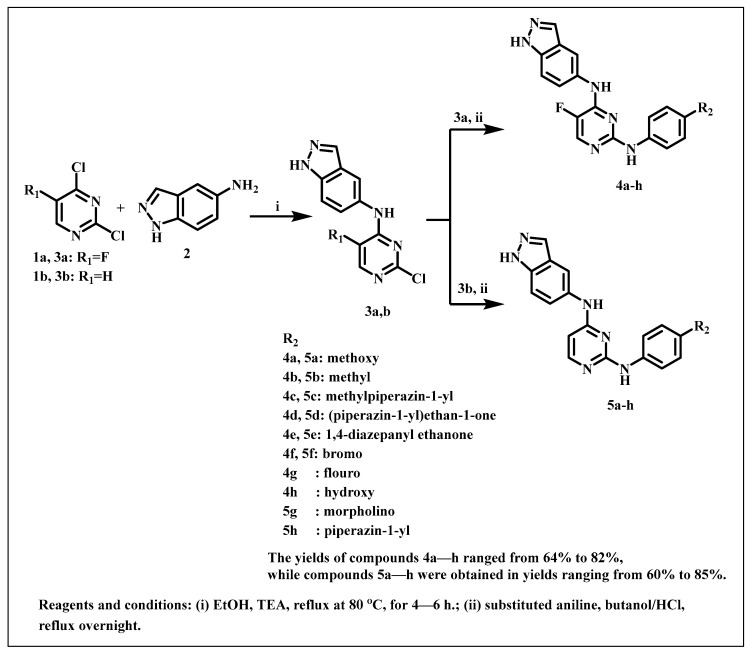
Synthetic routes of the substituted pyrimidinyl-indazol derivatives **4a**–**h**, **5a**–**h**.

**Scheme 2:**
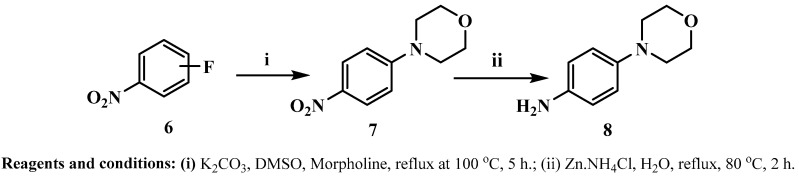
Synthetic routes of morpholino aniline.

The intermediate *N*-(2-chloro-5-substituted pyrimidin-4-yl)-1*H*-indazol -5-amine **3a**,**b** were synthesized according to the reported methodology [23]. The synthesis of the new compounds, 5-fluoro-*N4*-(*1H*-indazol-5-yl)-*N2*-(substituted) phenyl pyrimidines (**4a**–**h**) or *N4*-(1*H*-indazol-5-yl)-*N2*-(substituted) pyrimidines (**5a**–**h**), was carried out through a nucleophilic substitution reaction. Refluxing the appropriate aniline derivatives with compounds **3a** or **3b** in *n*-butanol, followed by acidifying the reaction mixture with a few drops of HCl, producing the desired compounds **4a**–**h** and **5a**–**h**, respectively, as illustrated in Scheme 1. While majority of anilines employed in this study were obtained commercially, morpholino aniline was synthesized according to a previously reported method [26] as depicted in Scheme 2. The chemical structures of the new derivatives were established using different spectral analyses (^1^H NMR, ^13^C NMR, IR, and mass spectrometry).

For example, ^1^H NMR of compounds **4a**–**h** are characterized by the presence of three NH groups, appearing as three singlets around δ 9–13.1 ppm, whereas the aromatic protons appeared around δ 6.5–8.3 ppm as singlets, doublets, or multiples and a singlet at 8.09–8.3 ppm is a characteristic signal of the fluoropyrimidine proton CH-6, singlet signal at the range of 7.9–8.09 ppm related to the indazole CH-3 and a third singlet signal at the range of 7.5–7.8 related to the indazole proton CH-4. While ^1^H NMR of compounds **5a**–**h** confirmed the presence of only two singlet signals, one at the range of 6.5 related to the indazole CH-4, the other in between 8.09–8.1 related to the indazole CH-3, besides the protons of three NH groups and the aromatic protons. ^13^C spectrum of the aromatic carbons appeared around δ 110–157 ppm, and for compounds that contain C=O, the signal appeared around δ 168–170 ppm (Supplementary Materials). The detailed NMR, IR, and mass spectral data of these compounds are given in the experimental section.

### 2.2. Biological Evaluation

**Figure 2:**
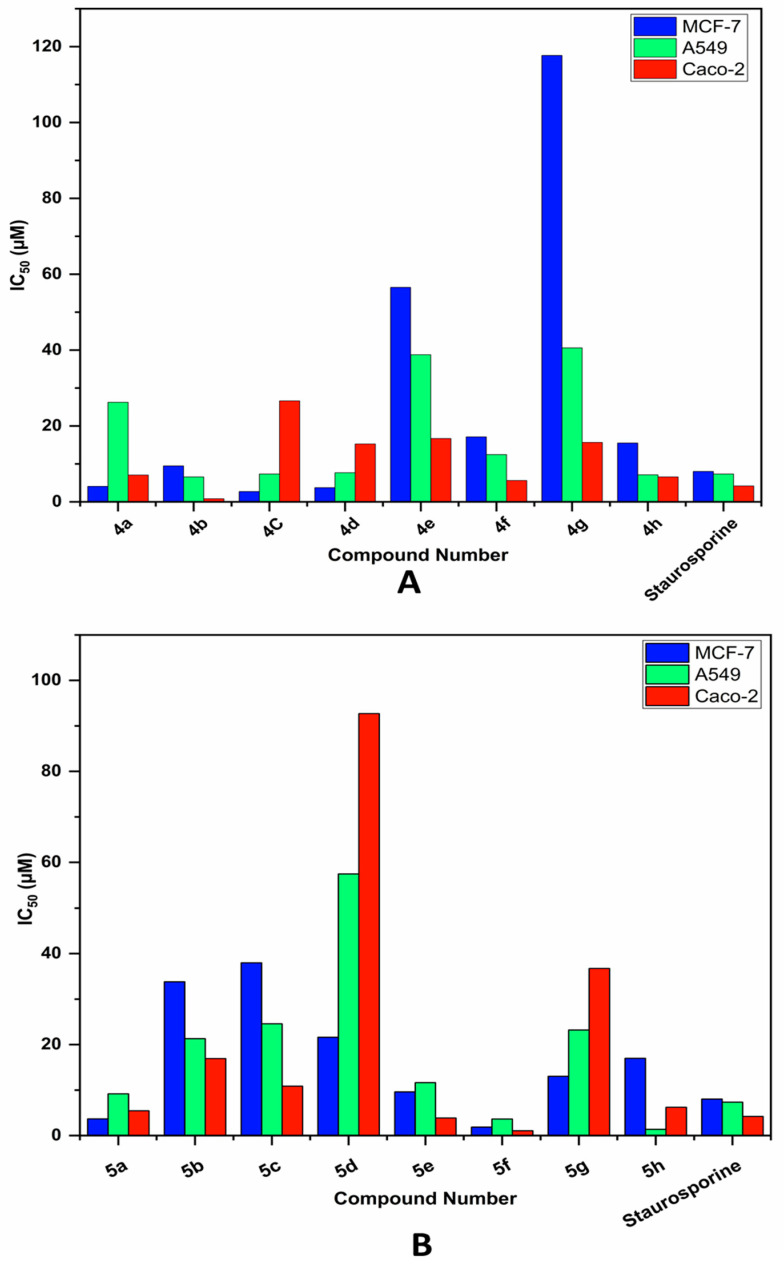
(**A**,**B**): In vitro cytotoxicity assay of anilinopyrimidine derivatives using the MTT Assay.

The cytotoxicity of the new indazol-pyrimidine derivatives **4a**–**h** and **5a**–**h** against different types of cancer cells was first assessed using the MTT assay [27]. The colorimetric 3-(4,5-dimethylthiazol-2-yl)-2,5-diphenyltetrazolium bromide (MTT) assay assesses the metabolic activity of cells. The recommended cancer cell lines for the MTT experiment were colon (Caco-2), breast (MCF-7), and epithelial lung (A549). After allowing the culture plate to incubate for 24 h, the anilinopyrimidine derivatives were evaluated at various concentrations (0.4 µM, 1.6 µM, 6.3 µM, 25 µM, and 100 µM), Using a Bioline ELISA plate reader, the optical density of the resultant solution was determined at 570 nm. The IC_50_ values, which are shown in Figure 2A,B, were calculated and compared in order to evaluate the cytotoxicity of each anilinopyrimidine derivative. The results presented in Table 1.

The obtained data showed that the compounds **4a**, **4c**, **4d**, **5a** and **5f** had potent cytotoxic activity against MCF-7 cell line, with IC_50_ values of 4.08, 2.73, 3.74, 3.67 and 1.85 µM respectively, showing greater activity than the reference drug Staurosporine with an IC_50_ value of 8.029 µM. Conversely, compounds, **4b**, **5e** and **5f** exhibited potent cytotoxic activity against Caco-2 cell line with IC_50_ values of 0.827, 3.869 and 1.056 µM, respectively, also surpassing the standard drug, which showed an IC_50_ value of 4.202 µM. Additionally, compounds **4b**, **5f** and **5h** displayed potent anti-proliferative activity against A549 cell line with IC_50_ values of 6.576, 3.628 and 1.378 µM, respectively, stronger than the reference drug with an IC_50_ value of 7.354 µM. Among all the compounds, **5f** demonstrated potent cytotoxic effects across all three cancer cell lines (MCF-7, A549, and Caco-2), with IC_50_ values of 1.858, 3.628 and 1.056 µM, respectively. These values are significantly lower than those of the reference compound which showed IC_50_ values of 8.029, 7.354, and 4.202 µM, for the same cell lines, indicating that 5f is markedly more effective.

#### 2.2.1. Molecular Dynamic and System Stability

**Figure 3:**
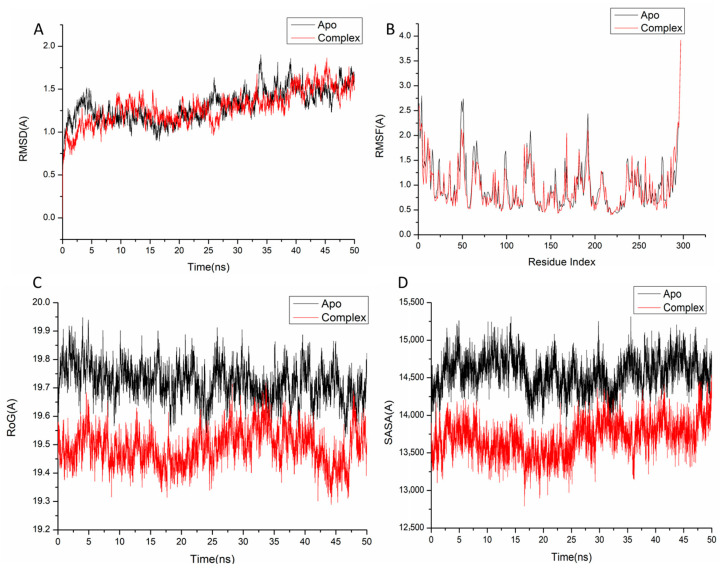
(**A**) Root Mean Square Deviation (RMSD) of Cα atoms in the protein backbone. (**B**) Root Mean Square Fluctuation (RMSF) of each residue’s Cα atom in the protein backbone. (**C**) Radius of Gyration (ROG) of Cα atoms. (**D**) Solvent Accessible Surface Area (SASA) of the Cα backbone atoms, shown relative to the initial minimized state (black) and over 50 ns for the catalytic binding site in the compound **5f**–c-KIT complex system (red).

A molecular dynamics simulation was conducted to evaluate the binding affinity of the synthesized compounds to the protein active site and to analyze their interactions and structural stability [28,29]. System stability was monitored to identify anomalous motions and minimize simulation artifacts The stability of each system was quantified by calculating the Root-Mean-Square Deviation (RMSD). The average RMSD values for the apo-protein and the **5f**–c-KIT complex were 1.33 ± 0.19 Å and 1.29 ± 0.20 Å, respectively (Figure 3A). These results suggest that the **5f**-bound complex adopted a more stable conformation than the other systems studied. To examine protein flexibility upon ligand binding and to analyze residue behavior and ligand interactions [30], the Root-Mean-Square Fluctuation (RMSF) was measured. The average RMSF values for the apo-protein and the **5f**–c-KIT complex were 1.00 ± 0.50 Å and 0.94 ± 0.46 Å, respectively (Figure 3B). This indicates that the **5f** complex exhibited lower residual fluctuation. The Radius of Gyration (Rg) was employed to assess the compactness and structural stability of the protein-ligand complex after ligand binding [31,32].

The average Rg values for the apo-protein and the **5f**–c-KIT complex were 19.71 ± 0.06 Å and 19.48 ± 0.06 Å, respectively (Figure 3C). This suggests that the 5f-bound complex has a highly rigid structure within the catalytic binding site. The stability of the protein’s hydrophobic core was further investigated by determining the Solvent Accessible Surface Area (SASA), which measures the protein surface area exposed to solvent [33]. The mean SASA values were 14,579.99 Å^2^ for the apo-protein and 13,722.27 Å^2^ for the **5f**–c-KIT complex. Together, the SASA, RMSD, RMSF, and Rg results consistently indicate that the 5f complex remained stable within the target receptor’s catalytic binding region.

#### 2.2.2. Binding Interaction Mechanism Based on Binding Free Energy Calculation

Binding free energies were calculated using the molecular mechanics energy method (MM/GBSA), a standard approach that leverages generalized Born and surface area continuum solvation for improved accuracy over docking scores [34]. Snapshots from the molecular dynamics trajectories were analyzed with the MM-GBSA tool in AMBER18. The resulting energy decomposition, detailed in Table 2, reveals that all components except for the solvation free energy (ΔGsolv) exhibit strongly negative values, confirming a thermodynamically favorable binding interaction.

An analysis of individual energy contributions revealed that the binding of compound **5f** to the target protein is primarily stabilized by a highly favorable van der Waals component, which is the major driver of the reported binding free energy (Table 2).

#### 2.2.3. Identification of the Critical Residues Responsible for Ligands Binding

**Figure 4:**
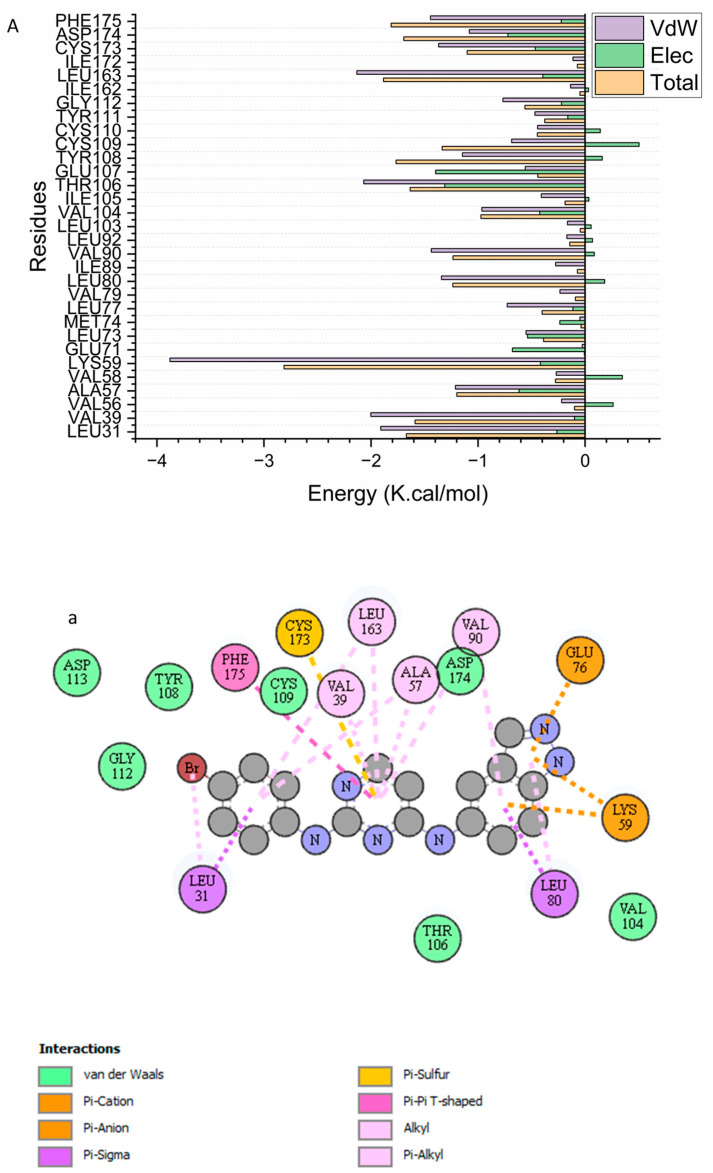
Per-residue breakdown plots demonstrate the specific energy contributions to the binding and stability of compound 5f within the catalytic binding region of the c-KIT receptor (**A**) The associated intermolecular interactions are shown in the corresponding figure (**a**).

To identify the key residues involved in inhibiting the receptor’s catalytic site, the individual energy contributions of compound **5f** binding to the enzyme were evaluated. This approach clarified the functional significance of specific active site residues. As shown in Figure 4, the most favorable energy contributions to binding are predominantly associated with these residues Leu 31 (−1.909 kcal/mol), Val 39 (−2.002 kcal/mol), Val 56 (−0.219 kcal/mol), Ala 57 (−1.21 kcal/mol), Val58 (−0.27 kcal/mol), Lys 59 (−3.878 kcal/mol), Leu 77 (−0.728 kcal/mol), Val79 (−0.235 kcal/mol), Leu 80 (−1.342 kcal/mol), Ile 89 (−0.275 kcal/mol), Val 90 (−1.437 kcal/mol), Leu 92 (−0.17 kcal/mol), Val 104 (−0.964 kcal/mol), Ile 105 (−0.409 kcal/mol), Thr 106 (−2.068 kcal/mol), Glu 107 (−0.559 kcal/mol), Tyr 108 (−1.146 kcal/mol), Cys 109 (−0.686 kcal/mol), Cys110 (−0.444 kcal/mol), Tyr 111 (−0.468 kcal/mol), Gly 112 (−0.768 kcal/mol), Leu 163 (−2.132 kcal/mol), Cys173 (−1.369 kcal/mol), Asp 174 (−1082 kcal/mol), and Phe 175 (−1.444 kcal/mol).

#### 2.2.4. Ligand–Residue Interaction Network Profiles

**Figure 5:**
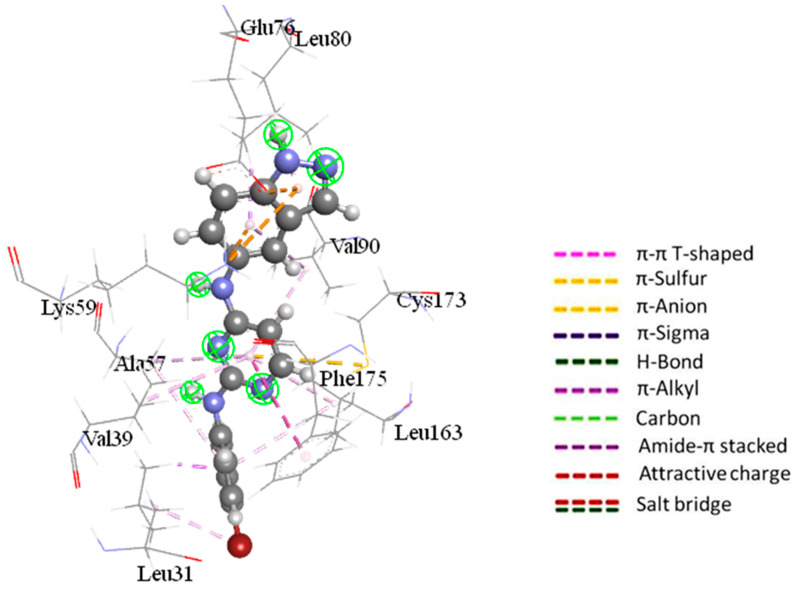
The interaction residue of compound 5f into the catalytic binding site of c-KIT receptor.

The primary objective of drug design is to modify therapeutic compounds to enhance bioavailability, reduce toxicity, and optimize pharmacokinetic properties [35]. Among the principal targets is the receptor tyrosine kinase (RTK) c-KIT. This protein, encoded by the proto-oncogene c-kit, functions as the receptor for stem cell factor (SCF) and regulates essential cellular processes such as proliferation, survival, and migration. Additionally, c-KIT is involved in physiological functions including hematopoiesis, pigmentation, and intestinal motility. Increasing evidence demonstrates that aberrant c-KIT activity, frequently resulting from mutations or overexpression, contributes to tumor development and progression in various human cancers [36]. Compound **5f** has established an api-alkyl interaction with Val90, Ala57, Leu 163, and Val 39, as shown in Figure 3. Additionally, compound **5f** and Phe 175 have developed a pi-pi T-shaped contact. Additionally, compound **5f** and Cys 173 have developed a pi-sulfur connection. With compound **5f**, the pharmacophoric hot spot residues Leu 31 and 80 have established pi-sigma and pi-alkyl interactions. Ultimately, compound **5f** has developed a Pi-cation contact with Glu 76 and Lys 59 Figure 5.

## 3. Experimental

### 3.1. Chemistry

Branstead 9100 electrothermal melting point device measured uncorrected melting points (°C). A JEOl-500 MHz NMR spectrometer (JEOL, Ltd., Akishima, Tokyo Japan), measured ^1^H and ^13^C spectra at 500 and 125 MHz, respectively. Chemical shifts are measured below tetramethylsilane (TMS) as the internal standard, in δ (ppm). *J* is coupling constant in Hz. Solvents included DMSO-d6. An Electron ionization Mass Spectrometry 70 electron volt made in Italy 2009 thermo scientific ISQ recorded positive and negative ion electrospray ionization mass spectra (Eims). Elemental analysis for carbon (C), hydrogen (H), and nitrogen (N) was performed at the Micro Analytical Center, Faculty of Science, Cairo University, Egypt.

#### 3.1.1. Synthesis of N-(2-chloro-5-substituted pyrimidin-4-yl)-1H-indazol-5-amine (**3a**,**b**)

The intermediate *N*-(2-chloro-5-substituted pyrimidin-4-yl)-1*H*-indazol -5-amines **3a**,**b** were synthesized by the reaction of 5-aminoindazole **2** with 5-flouro-2,4 dichloropyrimidine **1a** or 2,4 dichloropyrimidine **1b** in ethanol according to the reported methodology [23].

#### 3.1.2. Synthesis of Indazolyl-Pyrimidine Derivatives **4a**–**h** and **5a**–**h**

To a stirred solution of compound **3a** or **3b** (0.0018 mol) in butanol (25 mL), the appropriate aniline derivative (0.0018 mol) was added, followed by the addition of 4 drops of conc. HCl. Under constant stirring, the reaction mixture was refluxed overnight. Upon completion the mixture was cooled to room temperature, resulting in the formation of a precipitate. Which was collected by filtration, thoroughly washed with ethanol and/or ethyl acetate, and recrystallized from ethanol to afford the desired compounds **4a**–**h** or **5a**–**h**, respectively.

5-fluoro-*N4*-(*1H*-indazol-5-yl)-*N*2-(4-methoxyphenyl)pyrimidine-2,4-diamine **4a**

Yield: 72%; mp.: 271–273 °C; IR (υ_max_/cm^−1^): 3116 (br., 3NH), 3030 (CH, aromatic), 2886, 2831 (aliphatic CH), 1670 (C=N); ^1^H NMR (DMSO-*d*_6_, *δ* ppm): 3.7 (s, 3H, OCH_3_), 6.8 (d, *J* = 8.6, 2H, ph, CH-3,5), 7.3 (d, *J* = 9.1, 2H, ph, CH-2,6), 7.51–7.53 (m, 2H, Ar), 7.9 (d, *J* = 6.2, 1H, indazole, CH-7), 8.0 (s, 1H, indazole, CH-3), 8.3 (s, 1H, pyrimidine), 10.5, 10.7, 13.1 (3s, 3H, 3NH); ^13^C-NMR (DMSO-*d_6_*, *δ* ppm): 55.8 (OCH_3_), 110.7, 114.5, 115.3, 123.0, 123.6, 124.5, 129.7, 130.3, 133.9, 138.5, 140.2, 151.2, 153.0, 153.1, 157.0 (Ar-C); 351(M + 1) 100%, 352 (M + 2) 25%, Anal. Calcd. for: C_18_H_15_FN_6_O (350.36): C, 61.71; H, 4.32; N, 23.99, found: C, 61.58; H, 4.29; N, 23.68.

5-fluoro-*N4*-(*1H*-indazol-5-yl)-*N*2-(p-tolyl) pyrimidine-2,4-diamine **4b**

Yield: 70%; mp.: >300 °C; IR (υ_max_/cm^−1^): 3190 (br., 3NH), 3060 (CH, aromatic), 2890 (aliphatic CH), 1660 (C=N); ^1^H NMR (DMSO-*d*_6_, *δ* ppm): 2.2 (s, 3H, CH_3_), 6.9 (d, *J* = 8.1, 2H, ph, CH-3,5), 7.3 (d, *J* = 8.1, 2H, ph, CH-2,6), 7.5 (m, 2H, Ar), 7.9 (s, 1H, indazole, CH-3), 8.1 (m, 2H, 1H, pyrimidine and 1H, indazole, CH-7), 9.7, 10.0, 13.1 (3s, 3H, 3NH); ^13^C-NMR (DMSO-*d_6_*, *δ* ppm): 20.9 (CH_3_), 110.6, 114.3, 120.8, 120.7, 121.1, 123.2, 123.5, 129.5, 129.7, 130.9, 132.2, 133.8, 136.9, 152.0, 153.5, 160.2 (Ar-C); MS, *m*/*z* (%): 333 (M-1) (15), Anal. Calcd for C_18_H_15_FN_6_ (334.36): C, 64.66; H, 4.52; N, 25.14, found: C, 64.47; H, 4.30; N, 24.94.

5-fluoro-*N4*-(*1H*-indazol-5-yl)-*N2*-(4-(4-methylpiperazin-1-yl)phenyl)pyrimidine-2,4-diamine **4C**

Yield: 68%; mp.: >300 °C; IR (υ_max_/cm^−1^): 3400, 3114 (br., 3NH), 3029 (CH, aromatic), 2935, 2825 (aliphatic CH); 1612 (C=N); ^1^H NMR (DMSO-*d*_6_, *δ* ppm): 2.7 (s, 3H, CH_3_), 3.1–3.3 (m, 8H, 2(N-(CH_2_)_2_)), 6.7 (d, *J* = 9.5, 2H, ph, CH-3,5), 7.4–7.5 (m, 4H, Ar), 7.9 (s, 1H, indazole, CH-3), 8.0 (d, *J* = 3.8, 1H, indazole, CH-7), 8.2 (s, 1H, pyrimidine), 9.0, 9.3, 13.1 (3s, 3H, 3NH); ^13^C-NMR (DMSO-*d_6_*, *δ* ppm): 42.5 (NCH_3_), 46.3 (N(-CH_2_)_2_)_,_ 52.7 (N(-CH_2_)_2_)_,_ 110.5, 113.1, 117.0, 117.5, 120.9, 121.3, 123.3, 132.1, 133.7, 134.5, 137.7, 139.8, 139.9, 141.8, 144.8, 150.8, 156.0 (Ar-C); MS, *m*/*z* (%): 419 (M + 1) (75), Anal. Calcd for C_22_H_23_FN_8_ (418.48): C, 63.14; H, 5.54; N, 26.78, found: C, 62.89; H, 5.32; N, 26.50.

1-(4-(4-((4-((*1H*-indazol-5-yl)amino)-5-fluoropyrimidin-2-yl)amino)phenyl)piperazin-1-yl) ethan-1-one **4d**

Yield: 65%; mp.: 244–246 °C; IR (υ_max_/cm^−1^): 3126 (br., 3NH), 3037 (br, CH, aromatic), 2880 (aliphatic CH), 1737 (-COCH_3_), 1631 (C=N); ^1^H NMR (DMSO-*d*_6_, *δ* ppm): 2.0 (s, 3H, COCH_3_), 3.0 (m, 4H, N-(CH_2_)_2_, piperazinyl 2CH_2_-3,5), 3.5 (m, 4H, N-(CH_2_)_2_, piperazinyl 2CH_2_-2,6), 6.8 (m, 2H, Ar), 7.3 (d, *J* = 8.1, 2H, ph, CH-3,5), 7.5 (d, *J* = 8.1, 2H, ph, CH-2,6), 7.9 (s, 1H, indazole, CH-3), 8.09 (s, 1H, pyrimidine), 8.16 (d, *J* = 4.3,1H, indazole, CH-7), 9.7, 10.1, 13.1 (3s, 3H, 3NH); ^13^C-NMR (DMSO-*d_6_*, *δ* ppm): 21.7 (COCH_3_), 46.5 (N-(CH_2_)_2_), 50.5 (N-(CH_2_)_2_), 108.0, 110.6, 114.2, 117.2, 117.7, 122.5, 123.1, 123.4, 130.9, 133.8, 134.4, 138.1, 152.0, 153.4, 156.0, 168.8 (Ar-C and C=O); MS, *m*/*z* (%): 447 (M + 1) (25), Anal. Calcd for C_23_H_23_FN_8_O (446.49): C, 61.87; H, 5.19; N, 25.10, found: C, 61.67; H, 5.06; N, 24.91.

1-(4-(4-((4-((*1H*-indazol-5-yl)amino)-5-fluoropyrimidin-2-yl)amino)phenyl)-1,4-diazepan-1-yl)ethan-1-one **4e**

Yield: 64%; mp.: >300 °C; IR (υ_max_/cm^−1^): 3240 (br., 3NH), 3028 (CH, aromatic), 2880 (aliphatic CH), 1740 (-COCH_3_); 1638 (C=N); ^1^H NMR (DMSO-*d*_6_, *δ* ppm): 1.7 (m, 2H, diazepan, CH-6), 1.9 (s, 3H, -CO-CH_3_), 3.2 (m, 2H, diazepan), 3.4–3.6 (m, 6H, diazepan), 6.6 (d, *J* = 7, 2H, ph, CH-3,5), 7.1 (d, *J* = 7, 2H, ph, CH-2,6), 7.5 (m, 2H, indazole, CH-4,6), 8.0–8.1 (m, 3H, indazole, CH-3,7 and pyrimidine), 9.7, 10.4, 13.0 (3s, 3H, 3NH); ^13^C-NMR (DMSO-*d_6_*, *δ* ppm): 21.4, (COCH_3_), 24.6 (diazepan CH_2_-6), 44.5 (diazepan CH_2_-5), 47.8 (diazepan CH_2_-3), 72,0, 74.9 (diazepan CH_2_-7 and CH_2_-2), 110.7, 112.3, 115.2, 123.1, 123.6, 124.1, 124.3, 130.1, 134.0, 138.3, 152.7, 169.5, 169.6 (Ar-C); MS, *m*/*z* (%): 461 (M + 1) (95), Anal. Calcd for C_24_H_25_FN_8_O (460.52): C, 62.60; H, 5.47; N, 24.33, found: C, 62.48; H, 5.29; N, 24.10.

*N2*-(4-bromophenyl)-5-fluoro-*N4*-(*1H*-indazol-5-yl) pyrimidine-2,4-diamine **4f**

Yield: 82%; mp.: 291–293 °C; IR (υ_max_/cm^−1^): 3190, 3052 (br., 3NH), 3044 (CH, aromatic), 1614 (C=N); ^1^H NMR (DMSO-*d*_6_, *δ* ppm): 7.3 (d, *J* = 8.1, 2H, ph, CH-2,6), 7.5–7.4 (m, 4H, Ar), 8.0 (m 2H, Ar), 8.2 (s, br, 1H, pyrimidine), 10.0, 10.1 (2s, 2H, 2NH); ^13^C-NMR (DMSO-*d_6_*, *δ* ppm): 110.7, 110.9, 114.7, 114.8, 122.2, 122.4, 123.2, 123.8, 130.7, 131.7, 133.8, 138.3, 138.9, 152.1, 152.2, 153.0 (Ar-C); MS, *m*/*z* (%): 399 (M+) (37), Anal. Calcd for C_17_H_12_BrFN_6_ (399.23): C, 51.15; H, 3.03; N, 21.05, found: C, 50.91; H, 2.80; N, 20.80.

5-fluoro-*N2*-(4-fluorophenyl)-*N4*-(*1H*-indazol-5-yl)pyrimidine-2,4-diamine **4g**

Yield: 69%; mp.: 273–275 °C; IR (υ_max_/cm^−1^): 3298, 3115 (br., 3NH), 3052 (CH, aromatic), 1614 (C=N); ^1^H NMR (DMSO-*d*_6_, *δ* ppm): 7.5 (d, *J* = 8.1, 2H, ph, CH-2,6), 7.7 (m, 4H, Ar), 8.1 (m 2H, Ar), 8.2 (s, br, 1H, pyrimidine), 10.1, 10.3, 13.4 (3s, 3H, 3NH); ^13^C-NMR (DMSO-*d_6_*, *δ* ppm): 110.7, 114.3, 121.7, 121.8, 124.1, 124.6, 129.1, 129.9, 131.2, 132.6, 134.1, 134.5, 136.8, 137.2, 152.3, 153.9 (Ar-C); MS, *m*/*z* (%): 338 (M+) (5), Anal. Calcd for C_17_H_12_F_2_N_6_ (338.32): C, 60.35; H, 3.58; N, 24.84, found: C, 60.10; H, 3.42; N, 24.60.

4-((4-((*1H*-indazol-5-yl)amino)-5-fluoropyrimidin-2-yl)amino)phenol **4h**

Yield: 70%; mp.: 229–231 °C; IR (υ_max_/cm^−1^): 3250–3056 (br.,3NH, OH and CH, aromatic), 1623 (C=N); ^1^H NMR (DMSO-*d*_6_, *δ* ppm): 6.7 (d, *J* = 8.1, 2H, ph, CH-3,5), 7.1 (m, 3H, Ar), 7.5 (s, 1H, indazole, CH-4), 8.0 (s, 1H, indazole, CH-3), 8.2 (m, 2H, pyrimidine and indazole, CH-7), 10.01, 10.1, 10.5 (3s, 3H, 3NH), 13.2 (br, 1H, OH); ^13^C-NMR (DMSO-*d_6_*, *δ* ppm): 110.2, 114.3, 120.7, 120.8, 121.1, 123.2, 123.5, 129.5, 129.7, 130.9, 132.2, 133.8, 134.7, 136.9, 137.0, 152.0, 153.8 (Ar-C); MS, *m*/*z* (%): 336 (M+) (25), 337 (M + 1) (85), Anal. Calcd for C_17_H_13_FN_6_O (336.33): C, 60.71; H, 3.90; N, 24.99, found: C, 60.91; H, 4.02; N, 25.23.

*N4*-(*1H*-indazol-5-yl)-*N2*-(4-methoxyphenyl)pyrimidine-2,4-diamine **5a**

Yield: 59%; mp.: 207–209 °C; IR (υ_max_/cm^−1^): 3246 (br., 3NH), 3003 (CH, aromatic), 2827 (aliphatic CH), 1632 (C=N);^1^H NMR (DMSO-*d*_6_, *δ* ppm): 3.7 (s, 3H, OCH3), 6.5 (s, 1H, indazole, CH-4), 6.9 (d,, *J* = 8.6, 2H, ph, CH-3,5), 7.3 (d, *J* = 8.1, 2H, ph, CH-2,6), 7.4–7.5 (m, 2H, Ar), 7.8 (br, 2H, Ar), 8.0 (br, 1H, indazole, CH-7), 10.4, 11.1, 13.0 (3s, 3H, 3NH); ^13^C-NMR (DMSO-*d_6_*, *δ* ppm): 55.8 (OCH_3_), 99.8, 110.4, 115.9, 123.1, 123.2, 124.3, 124.3, 128.9, 129.7, 130.3, 133.7, 138.4, 140.5, 151.8, 153.5 (Ar-C); MS, *m*/*z* (%): 332 (M+) (25), Anal. Calcd for C_18_H_16_N_6_O (332.37): C, 65.05; H, 4.85; N, 25.29, found: C, 64.92; H, 4.65; N, 25.08.

*N4*-(*1H*-indazol-5-yl)-*N2*-(p-tolyl)pyrimidine-2,4-diamine **5b**

Yield: 63%; mp.: 267–269 °C; IR (υ_max_/cm^−1^): 3300 (br., 3NH), 3040 (CH, aromatic), 2960 (aliphatic CH),1643 (C=N);^1^H NMR (DMSO-*d*_6_, *δ* ppm): 2.2 (s, 3H, CH_3_), 6.5 (s, 1H, indazole, CH-4), 7.1 (d, *J* = 8.1, 2H, ph, CH-3,5), 7.3 (d, *J* = 7.1, 2H, ph, CH-2,6), 7.4 (d, *J* = 8.6, 1H, pyrimidine, CH-5), 7.5 (d, *J* = 8.5, 1H, indazole, CH-6), 7.9 (m, 2H, Ar), 8.0 (s, 1H, indazole, CH-3), 10.4, 10.9, 13.1 (3s, 3H, 3NH); ^13^C-NMR (DMSO-*d_6_*, *δ* ppm): 20.9 (CH_3_), 99.5, 110.7, 111.4, 115.3, 122.9, 123.0, 124.7, 129.5, 132.2, 133.8, 138.0, 143.3, 149.1, 150.3, 155.7 (Ar-C); MS, *m*/*z* (%): 316 (M+) (30), Anal. Calcd for C_18_H_16_N_6_ (316.37): C, 68.34; H, 5.10; N, 26.56, found: C, 68.52; H, 5.34; N, 26.73_._

*N4*-(*1H*-indazol-5-yl)-*N2*-(4-(4-methylpiperazin-1-yl)phenyl)pyrimidine-2,4-diamine **5C**

Yield: 60%; mp.: 293–295 °C; IR (υ_max_/cm^−1^): 3218 (br., 3NH), 3038 (CH, aromatic), 2896 (aliphatic CH), 1636 (C=N); ^1^H NMR (DMSO-*d*_6_, *δ* ppm): 2.4 (s, 3H, N-CH_3_), 2.7–3.9 (m, 8H, 2(N-(CH_2_)_2_) 6.5 (s, 1H, indazole, CH-4), 6.9 (d, *J* = 7.6, 2H, ph, CH-3,5), 7.3 (d, *J* = 7.1, 2H, ph, CH-2,6), 7.4 (m, 2H, Ar), 7.9 (m, 2H, Ar), 8.1 (s, 1H, indazole, CH-3), 10.5, 11.1, 13.1 (3s, 3H, 3NH); ^13^C-NMR (DMSO-*d_6_*, *δ* ppm): 42.5 (CH_3_), 48.1(N(CH_2_)_2_), 52.5 (N(CH_2_), 99.9, 111.0, 113.3, 116.8, 117.2, 122.5, 123.1, 124.4, 129.9, 131.3, 134.1, 138.0, 144.1, 147.4, 153.9, 161.3 (Ar-C); MS, *m*/*z* (%): 399 (M-1) (10) Anal. Calcd for C_22_H_24_N_8_ (400.49): C, 65.98; H, 6.04; N, 27.98, found: C, 65.69; H, 5.83; N, 27.76.

1-(4-(4-((4-((*1H*-indazol-5-yl)amino)pyrimidin-2-yl)amino)phenyl)piperazin-1-yl)ethan-1-one **5d**

Yield: 66%; mp.: >300 °C; IR (υ_max_/cm^−1^): 3145 (br., 3NH), 3042 (br, CH, aromatic), 2890 (aliphatic CH), 1739 (COCH_3_), 1635 (C=N); ^1^H NMR (DMSO-*d*_6_, *δ* ppm): 2.01 (s, 3H, COCH_3_), 3.05–3.2 (m, 4H, N-(CH_2_)_2_), 3.54–3.56 (m, 4H, N-(CH_2_)_2_), 6.5 (s, 1H, indazole, CH-4), 6.9 (d, *J* = 8.6, 2H, ph, CH-3,5), 7.3 (d, *J* = 8.1, 2H, ph, CH-2,6), 7.4 (m, 2H, Ar), 7.9 (m, 2H, Ar), 8.1 (s, 1H, indazole, CH-3), 10.4, 11.1, 13.7 (3s, 3H, 3NH); ^13^C-NMR (DMSO-*d_6_*, *δ* ppm): 21.7 (-COCH_3_), 46.1 (N(CH_2_)_2_), 49.3 (N(CH_2_)_2_), 110.9, 113.4, 116.5, 116.7, 117.3, 122.4, 123.1, 124.8, 128.7, 131.0, 133.9, 138.0, 142.9, 148.9, 153.4, 161.2, 161.4, 168.8 (Ar-C); MS, *m*/*z* (%): 429 (M + 1) (95), Anal. Calcd for C_23_H_24_N_8_O (428.50): C, 64.47; H, 5.65; N, 26.15, found: C, 64.61; H, 5.80; N, 26.32.

1-(4-(4-((4-((*1H*-indazol-5-yl)amino)pyrimidin-2-yl)amino)phenyl)-1,4-diazepan-1-yl)ethan-1-one **5e**

Yield: 57%; mp.: 264–266 °C; IR (υ_max_/cm^−1^): 3200–3030 (br., 3NH and CH, aromatic), 2871 (aliphatic CH), 1741 (COCH_3_), 1639 (C=N); ^1^H NMR (DMSO-*d*_6_, *δ* ppm): 1.8 (m, 2H, CH_2_ diazepan, CH-6) 2.08 (s, 3 H, COCH_3_), 2.3, 2,6 (m, 2H, CH_2_ diazepan), 3.17 (m, 4 H, 2CH_2_ diazepan), 4.08–4.1 (m, 2 H, CH_2_, diazepan), 6.5 (s, 1H, indazole, CH-4), 7.62–7.46 (m, 7H, Ar), 8.02 (s, 1H, NH), 8.09 (s,1H, indazole, CH-3), 8.28 (d, 1 H, d, *J* = 3.5, indazole, CH-7), 10.00, 13.09 (2s, 2 H, 2NH); ^13^C-NMR (DMSO-*d_6_*, *δ* ppm): 14.3 (CH_3_), 19.1 (CH_2-_6), 21.2 (CH_2_-5), 35.2 (CH_2_-3), 55.9 (CH_2_-7), 60.8 (CH_2_-2), 100.0, 110.8, 113.2, 114.7, 114.9, 115.9, 122.3, 122.6, 123.1, 124.9, 130.9, 133.8, 142.3, 153.2, 159.2, 159.6, 161.1 (Ar-C); MS, *m*/*z* (%): 441 (M-1) (32), Anal. Calcd for C_24_H_26_N_8_O (442.53): C, 65.14; H, 5.92; N, 25.32, found: C, 64.82; H, 5.72; N, 25.19.

*N2*-(4-bromophenyl)-*N4*-(*1H*-indazol-5-yl)pyrimidine-2,4-diamine **5f**

Yield: 58%; mp.: 232–234 °C; IR (υ_max_/cm^−1^): 3240 (br, 3NH), (CH, aromatic), 1639 (C=N); ^1^H NMR (DMSO-*d*_6_, *δ* ppm): 6.5 (s, 1H, indazole, CH-4), 7.4–7.5 (m, 5H, Ar), 7.58 (d, *J* = 9, 1H, pyrimidine, CH-5), 7.9 (m, 2H, Ar), 8.0 (s, 1H, indazole, CH-3), 10.7, 11.0, 13.1 (3s, 3H, 3NH); ^13^C-NMR (DMSO-*d_6_*, *δ* ppm): 100.0, 110.5, 113.9, 115.8, 120.8, 123.3, 130.8, 133.9, 134.6, 138.1, 140.8, 145.2, 153.1, 154.3, 158.0 (Ar-C); MS, *m*/*z* (%): 381 (M+) (4), Anal. Calcd for C_17_H_13_BrN_6_ (381.24): C, 53.56; H, 3.44; N, 22.04, found: C, 53.48; H, 3.24; N, 21.84.

*N4*-(*1H*-indazol-5-yl)-*N2*-(4-morpholinophenyl)pyrimidine-2,4-diamine **5g**

Yield: 60%; mp.: 298–300 °C; IR (υ_max_/cm^−1^): 3308 (br, 3NH), 3096 (CH, aromatic), 2814 (aliphatic CH); 1630 (C=N); ^1^H NMR (DMSO-*d*_6_, *δ* ppm): 3.0 (br, 4H, N-(CH_2_)_2_), 3.7 (br, 4H, O-(CH_2_)_2_), 6.5 (s, 1H, indazole, CH-4), 6.8 (br, 2H, ph, CH-3,5), 7.3 (br, 2H, ph, CH-2,6), 7.4 (m, 2H, Ar), 7.9 (br, 2H, indazole, CH-7 and pyrimidine CH-4), 8.1 (s, 1H, indazole, CH-3), 10.3, 11.1, 13.1 (3s, 2H, 3NH); ^13^C-NMR (DMSO-*d_6_*, *δ* ppm): 49.1 (N(-CH_2_)_2_), 66.6 (O(-CH_2_)_2_), 99.5, 110.8, 113.3, 115.8, 122.4, 123.2, 124.8, 128.8, 131.1, 134.0, 138.0, 143.4, 149.1, 153.7, 161.2 (Ar-C); MS, *m*/*z* (%): 387 (M+) (15), 388 (100), Anal. Calcd for C_21_H_21_N_7_O (387.45): C, 65.10; H, 5.46; N, 25.31, found: C, 64.90; H, 5.25; N, 25.19.

*N4*-(*1H*-indazol-5-yl)-*N2*-(4-(piperazin-1-yl)phenyl)pyrimidine-2,4-diamine **5h**

Yield: 73%; mp.: 247–249 °C; IR (υ_max_/cm^−1^): 3195 (br., 4NH), 3057 (CH, aromatic), 2823 (aliphatic CH), 1608 (C=N); ^1^H NMR (DMSO-*d*_6_, *δ* ppm): 2.0 (s, 1H, NH, piperizin), 2.9 (m, 6H, 3CH_2_, piperizine), 3.5 (m, 2H, CH_2,_ piperizine), 6.7 (m, 2H, Ar), 7.4 (m, 4H, Ar), 7.5 (d, *J* = 9.1, 1H, indazole, CH-6), 7.94–7.99 (m, 2H, Ar), 8.1 (d, *J* = 5.7, 1H, indazole, CH-7), 8.8, 9.2, 12.9 (3s, 3H, 3NH); ^13^C-NMR (DMSO-*d_6_*, *δ* ppm): 45.6 (N(CH_2_)_2_), 50.2 (N(CH_2_)_2_), 99.9, 116.4, 117.0, 120.8, 120.9, 123.2, 123.3, 132.3, 133.6, 133.7, 140.6, 140.7, 146.1, 150.6, 156.5, 168.7 (Ar-C); MS, *m*/*z* (%): 388 (M + 2) (100), Anal. Calcd for C_21_H_22_N_8_ (386.46): C, 65.27; H, 5.74; N, 29.00, found: C, 65.09; H, 5.56; N, 28.80.

### 3.2. Biological Assays

#### MTT Assay

The American Tissue Type Culture Collection (ATCC) provided the cell lines, which originated in different tissue types and included MCF-7 (human breast adenocarcinoma cells), Caco-2 (human colorectal adenocarcinoma cells), and A459 (epithelial lung carcinoma cells). The company Dulbecco acquired its Modified Eagle Medium (*DMEM*, Gibco, Grand Island, NY, USA) from Sigma-Aldrich (St. Louis, MO, USA). Halogen lamp 8V/50W provided fetal bovine serum (FBS). All other chemicals and reagents, including insulin, were acquired from Sigma-Aldrich (St. Louis, MO, USA). Penicillin/streptomycin (as antibiotic supplements), 10% FBS, 10 µg/mL insulin, and DMEM were added to 75 cm^2^ flasks containing the tumor cell lines. The cell temperature once the compounds were produced and prepared for testing. First, each flask’s growth medium for each cell line was removed and put into a centrifuge tube. Trypsin 0.53 mM EDTA solution (0.25% (*w*/*v*)) was used to rinse the cell layer in the flask to remove any remaining serum that contained the trypsin inhibitor. After washing, the flask holding the cells was filled with 2–3 mL of Trypsin EDTA solution, and the cells were seen under an inverted microscope until the cell layer was distributed. The cell layers were spread out, and then 6–8 mL of complete growth medium was added. The cells were then carefully pipetted out and moved to the centrifuge tube containing the culture medium and cells (from the first sub-culturing step). After centrifuging the suspension for 5 min at 1250× *g*, the supernatant was disposed of. The cell pellet was well mixed using pipetting in 10 mL of brand-new growth medium. The culture suspension was transferred to individual 25-cm 2 vented flasks and incubated for 24 h at 37 °C with 5% CO_2_. The cells were exposed to increasing concentrations of the chemical under evaluation before being cultured for 48 h at 37 °C [37]. Before performing the MTT experiment, the plates were inspected under an inverted microscope. The in vitro cytotoxicity of the generated compounds against a range of cell lines, including MCF-7, A459, and Caco-2, was monitored using the 3-(4,5-Dimethylthiazol-2-Yl)-2,5-diphenyltetrazolium bromide (MTT) assay, a colorimetric assay that assesses cell metabolic activity. Cell lines were permitted to reach 75% confluency prior to receiving either the novel anilinopyrimidine derivatives treatment or a placebo. In a 96-well plate, plate cells (densities 1.2–1.8 × 10,000 cells/well) consisted of a volume of 100 µL complete growth media plus 100 µL of the tested compounds (anilinopyrimidine derivatives) in each well, with the exception of four wells that used DMSO as a negative control in place of the tested compounds. However, since staurosporine is a broad-spectrum protein kinase inhibitor that can induce apoptosis via a number of apoptotic pathways [38], it was employed as a positive control. The anilinopyrimidine derivatives employed in the MTT assays had concentrations of 100 µM, 25 µM, 6.3 µM, 1.6 µM, and 0.4 µM, in that order. Before performing the MTT experiment, the plates were incubated for 24 h at 37 °C after the anilinopyrimidine derivatives were added in triplicate. Following a 24-h incubation period, each well received 10 µL (10% of the culture medium volume) of 5-diphenyl-tetrazolium bromide (MTT), which was then left to incubate for an additional 2–4 h. Following the incubation period, 100 µL of DMSO was applied to each well to dissolve the formazan crystals that were produced after the results were removed. The 96-well culture plate (Boekel Scientific, Philadelphia, PA, USA) was placed on a low-speed gyrator shaker to facilitate the dissolution of formazan crystals. Periodically, the plate was gently pipetted up and down to guarantee the crystals were completely dissolved. Using a Bioline ELISA R206—Microplate Reader/Elisa Reader, the optical density of the resultant solution was determined at 570 nm. The optical density of the formazan crystal solution is closely correlated with the quantity of live cells that remain in solution. To calculate the percentage of cell viability, the concentration of formazan crystals generated in the sample with anilinopyrimidine derivatives was compared to the results from the sample used as the negative control. It was assumed that the cell viability of the negative controls was 100%. The IC_50_ values were calculated using an IC_50_ calculator.

### 3.3. System Preparation and Molecular Docking

The three-dimensional structure of c-Kit tyrosine kinase (PDB ID: 1T46) was obtained from the Protein Data Bank [39]. Using UCSF Chimera 1.16 [40], the system’s pH was adjusted to 7.5 with the PROPKA tool (version 3.0) [41]. The two-dimensional molecular structure was drawn in ChemBioDraw Ultra 12.1 [42] and subsequently prepared for docking through energy minimization. This was performed using the steepest descent algorithm and the MMFF94 force field within the Avogadro software suite 1.1.0 [43]. Finally, hydrogen atoms were removed using UCSF Chimera [40] to complete the pre-docking preparation.

### 3.4. Molecular Docking

Molecular docking simulations were performed with AutoDock Vina and AutoDock MGL Tools 1.5.7 [44]. Gasteiger partial charges were assigned to all atoms for the duration of the docking process [45]. The atom types required for AutoDock were defined using the AutoDockTools (ADT) graphical interface [46]. A grid box with center coordinates of (27.59, 25.98, 43.054) and dimensions of 20 Å^3^ was established to encompass the binding site. The exhaustiveness was set to 8. Docked conformations were generated and ranked by their binding affinity using the Lamarckian genetic algorithm [47].

### 3.5. Molecular Dynamic (MD) Simulations

Molecular Dynamics (MD) simulations are a powerful tool in biological research, allowing for the detailed analysis of atomic and molecular motions that are difficult to capture with other experimental methods [48]. These simulations offer critical insights into the dynamic behavior of biological systems, including conformational rearrangements and interaction dynamics [48]. All MD simulations were conducted using the GPU-accelerated PMEMD engine within the AMBER 18 software suite [49]. Partial atomic charges for the ligands were derived using the General Amber Force Field (GAFF) via the ANTECHAMBER tool [50]. Each system was prepared using the Leap module in AMBER 18, which placed the solute in an orthorhombic box of TIP3P water molecules, maintaining a minimum buffer distance of 10 Å from the box edges. Systems were neutralized by adding Na^+^ and Cl^−^ counter ions.

An energy minimization protocol was then implemented, beginning with 2000 steps of restrained minimization (500 kcal/mol restraint force), followed by an additional 1000 steps of unrestrained minimization using the conjugate gradient algorithm. Subsequently, each system was gradually heated from 0 K to 300 K over 500 ps under constant volume (NVT) conditions, with a harmonic restraint of 10 kcal/mol applied to the solute and a collision frequency set to 1 ps^−1^. This was followed by a 500 ps equilibration period at 300 K. Production simulations were performed in the isothermal-isobaric (NPT) ensemble for 50 nanoseconds. The Berendsen barostat was used to maintain pressure at 1 bar [51]. The SHAKE algorithm constrained bonds involving hydrogen atoms [52], permitting a 2 fs integration time step. Simulations employed a single-precision model, with temperature regulated at 300 K by the Langevin thermostat (collision frequency = 1 ps^−1^) and pressure controlled with a coupling constant of 2 ps [53].

### 3.6. Post-MD Analysis

After preserving trajectories obtained from MD simulations at 1 ps intervals, the trajectories were analyzed using the CPPTRAJ module of the AMBER18 suite. The Origin version 9.1 data analysis software [54] and Chimera 1.16 were employed to produce all graphs and visualizations.

### 3.7. Thermodynamic Calculation

The Molecular Mechanics/Poisson-Boltzmann Surface Area (MM/PBSA) and Molecular Mechanics/Generalized Born Surface Area (MM/GBSA) methods are established computational techniques for estimating ligand-binding affinities [55,56]. These approaches calculate the binding free energy (ΔG) from molecular simulation trajectories by statistically averaging the energies of the complex, ligand, and receptor. For this study, the binding free energy was derived by analyzing 500 snapshots extracted from the entire 50 ns simulation trajectory, with the overall ΔG evaluated according to the following equation [57].

(1) $$∆G_{bind}=G_{complex}-G_{receptor}-G_{ligand}$$

(2) $$∆G_{bind}=E_{gas}+G_{sol}-TS$$

(3) $$E_{gas}=E_{int}+E_{vdw}+E_{ele}$$

(4) $$G_{sol}=G_{GB}+G_{SA}$$

(5) $$G_{SA}=\gamma SASA$$

The molecular mechanics energy in the gas phase (E_gas_) is a composite term comprising internal (E_int_), electrostatic (E_ele_), and van der Waals (E_vdw_) energy components, as derived from the FF14SB force field. The solvation free energy (G_sol_) was decomposed into polar (G_GB_) and non-polar (G_SA_) contributions. The non-polar component (G_SA_) was calculated from the Solvent Accessible Surface Area (SASA) using a solvent probe radius of 1.4 Å [58]. The polar solvation term (G_GB_) was obtained by solving the Generalized Born (GB) equation. The total entropy of the solute is denoted as S, and T represents the temperature. Per-residue energy decomposition analysis was conducted using the MM/GBSA methodology in Amber18 to quantify the contribution of individual residues to the total binding free energy [59].

## 4. Conclusions

In the present work, a series of novel indazol-pyrimidine derivatives were synthesized and evaluated for their in vitro cytotoxic activity against three human cancer cell lines: MCF-7 (breast adenocarcinoma), A549 (lung carcinoma), and Caco-2 (colon adenocarcinoma), by means of the MTT assay. The results indicated that five compounds—**4a**, **4c**, **4d**, **5a**, and **5f**—exhibited potent cytotoxic activity against MCF-7 cell line, showing greater potency than the reference drug Staurosporine. Notably, compound **4b** showed the highest activity against Caco-2 cell line, with an IC_50_ value of 0.827 µM, outperforming the reference drug which had an IC_50_ value of 4.202 µM. in addition, compound **5h** exhibited the most significant anti-proliferative activity against A549 cell line, with an IC_50_ value of 1.378 µM, demonstrating superior efficacy than the reference drug, with an IC_50_ value of 7.354 µM. Among all the synthesized derivatives, compound **5f** revealed robust anti-proliferative activity across the three tested cancer cell lines, showing IC_50_ values of 1.858, 3.628, and 1.056 µM, respectively. In contrast, the reference drug exhibited IC_50_ values of 8.029, 7.354 and 4.202 µM respectively. demonstrating promising anti-proliferative potency. Furthermore, molecular docking and molecular dynamics simulation studies of compound **5f** revealed strong binding affinity and favorable interactions within the active site of the c-Kit tyrosine kinase receptor. These findings suggest that compound **5f** could serve as a new scaffold for designing potent c-Kit tyrosine kinase inhibitors in anti-cancer therapy.

## Figures and Tables

**Table 1:** In vitro cytotoxicity data of anilinopyrimidine derivatives against MCF-7, A549, and Caco-2 cells.

Compound	Cytotoxicity IC_50_ (µM)		
	MCF-7	A549	Caco-2
**4a**	4.086 ± 0.16	26.24 ± 1.15	7.071 ± 0.32
**4b**	9.474 ± 0.38	6.576 ± 0.29	0.827 ± 0.04
**4C**	2.738 ± 0.11	7.354 ± 0.32	26.62 ± 1.22
**4d**	3.744 ± 0.15	7.696 ± 0.34	15.25 ± 0.7
**4e**	56.55 ± 2.24	38.79 ± 1.7	16.69 ± 0.76
**4f**	17.13 ± 0.68	12.46 ± 0.55	5.616 ± 0.26
**4g**	117.7 ± 4.66	40.59 ± 1.78	15.66 ± 0.72
**4h**	15.52 ± 0.61	7.136 ± 0.31	6.592 ± 0.3
**5a**	3.67 ± 0.15	9.18 ± 0.4	5.435 ± 0.25
**5b**	33.83 ± 1.34	21.3 ± 0.93	16.91 ± 0.77
**5C**	37.99 ± 1.5	24.58 ± 1.08	10.87 ± 0.5
**5d**	21.61 ± 0.86	57.52 ± 2.52	92.69 ± 4.25
**5e**	9.603 ± 0.38	11.65 ± 0.51	3.869 ± 0.18
**5f**	1.858 ± 0.05	3.628 ± 0.16	1.056 ± 0.05
**5g**	13.05 ± 0.52	23.21 ± 1.02	36.74 ± 1.68
**5h**	16.98 ± 0.67	1.378 ± 0.06	6.226 ± 0.29
Staurosporine	8.029 ± 0.12	7.354 ± 0.12	4.202 ± 0.19

**Table 2:** Shows the calculated binding energy of compound **5f** at the catalytic site of the c-KIT receptor.

Energy Components (kcal/mol)					
**c-KI**					
**Complex**	**ΔE_vdW_**	**ΔE_elec_**	**ΔG_gas_**	**ΔG_solv_**	**ΔG_bind_**
**Compound 5f–C-kit**	−59.79 ± 0.01	−8.48 ± 0.04	−68.27 ± 0.03	18.89 ± 0.02	−49.38 ± 0.05

∆E_vdW_ = van der Waals energy; ∆E_ele_ = electrostatic energy; ∆G_solv_ = solvation free energy; ∆G_bind_ = calculated total binding free energy.

## Data Availability

The data that support the findings of this study are available from the corresponding author upon reasonable request.

## Supplementary Materials

The following supporting information can be downloaded at: https://www.mdpi.com/article/10.3390/molecules30183773/s1.
