# Supplementary material for: Indazol-Pyrimidine Hybrids: Design, Synthesis, and Antiproliferative Activity Against Human Cancer Cell Lines
Source: Molecules. 2025 Sep 17;30(18):3773. doi: 10.3390/molecules30183773 (PMC12472346; doi:10.3390/molecules30183773)
Supplement: Supplementary file 1 [file molecules-30-03773-s001.zip › molecules-3815395-supplementary.pdf]

Supplementary Material

Indazol-Pyrimidine Hybrids: Design, Synthesis, and Antiproliferative Activity Against Human Cancer Cell Lines

Contents: ^{1}H and ^{13}C-NMR (DMSO-*d6*, δ ppm) spectra

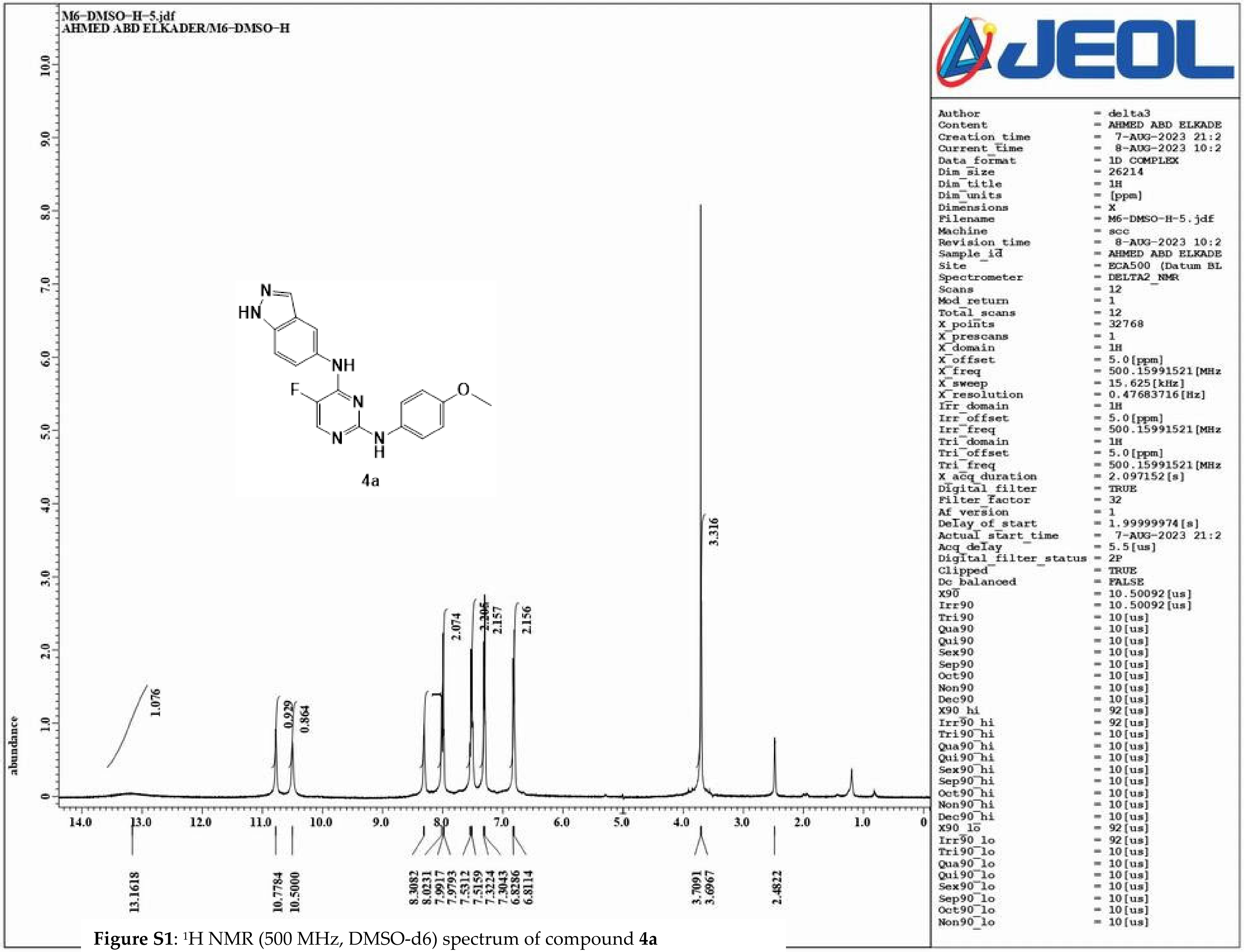

Figure S1: ^{1}H NMR (500 MHz, DMSO-d6) spectrum of compound **4a**

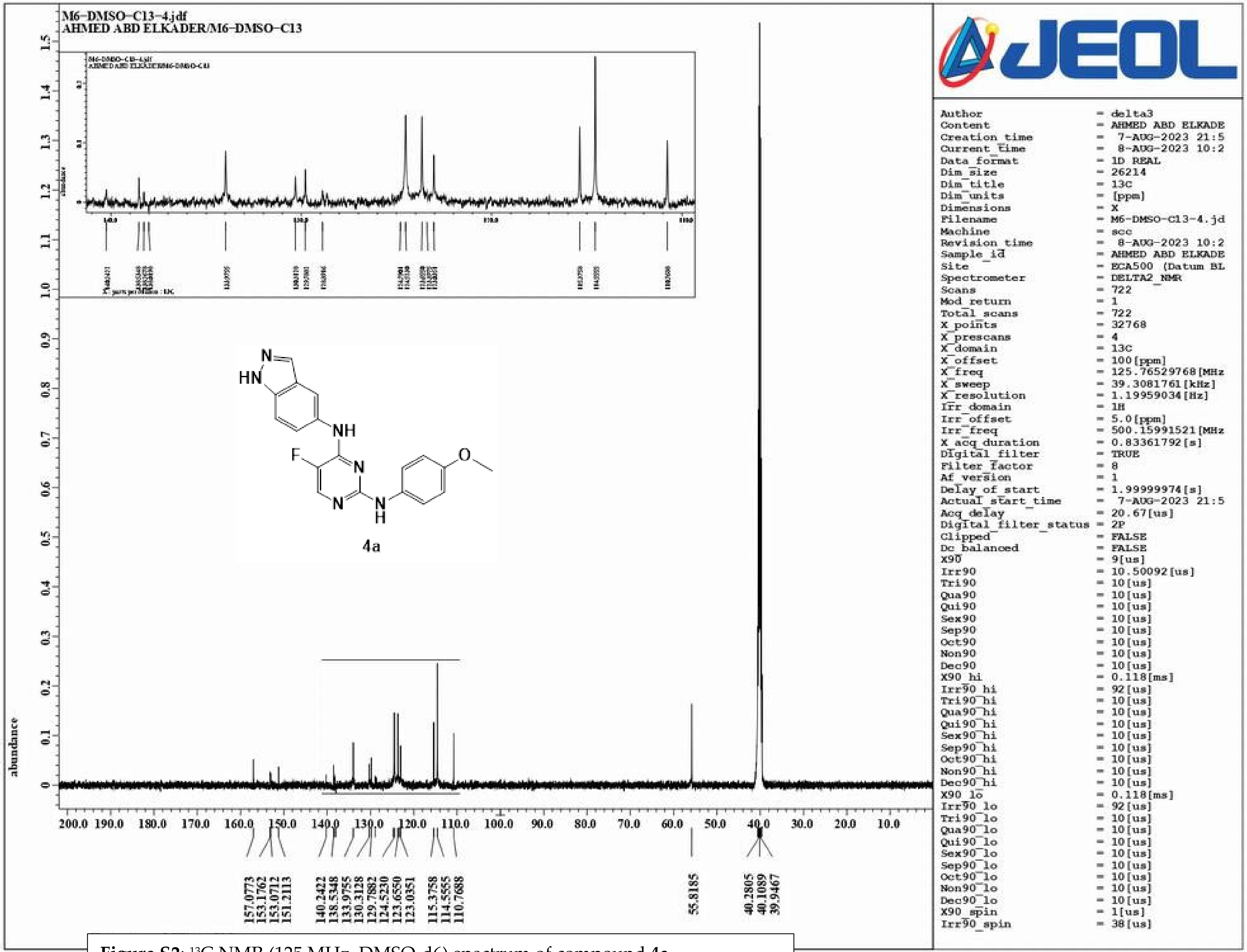

Figure S2: ^{13}C NMR (125 MHz, DMSO-d6) spectrum of compound **4a**

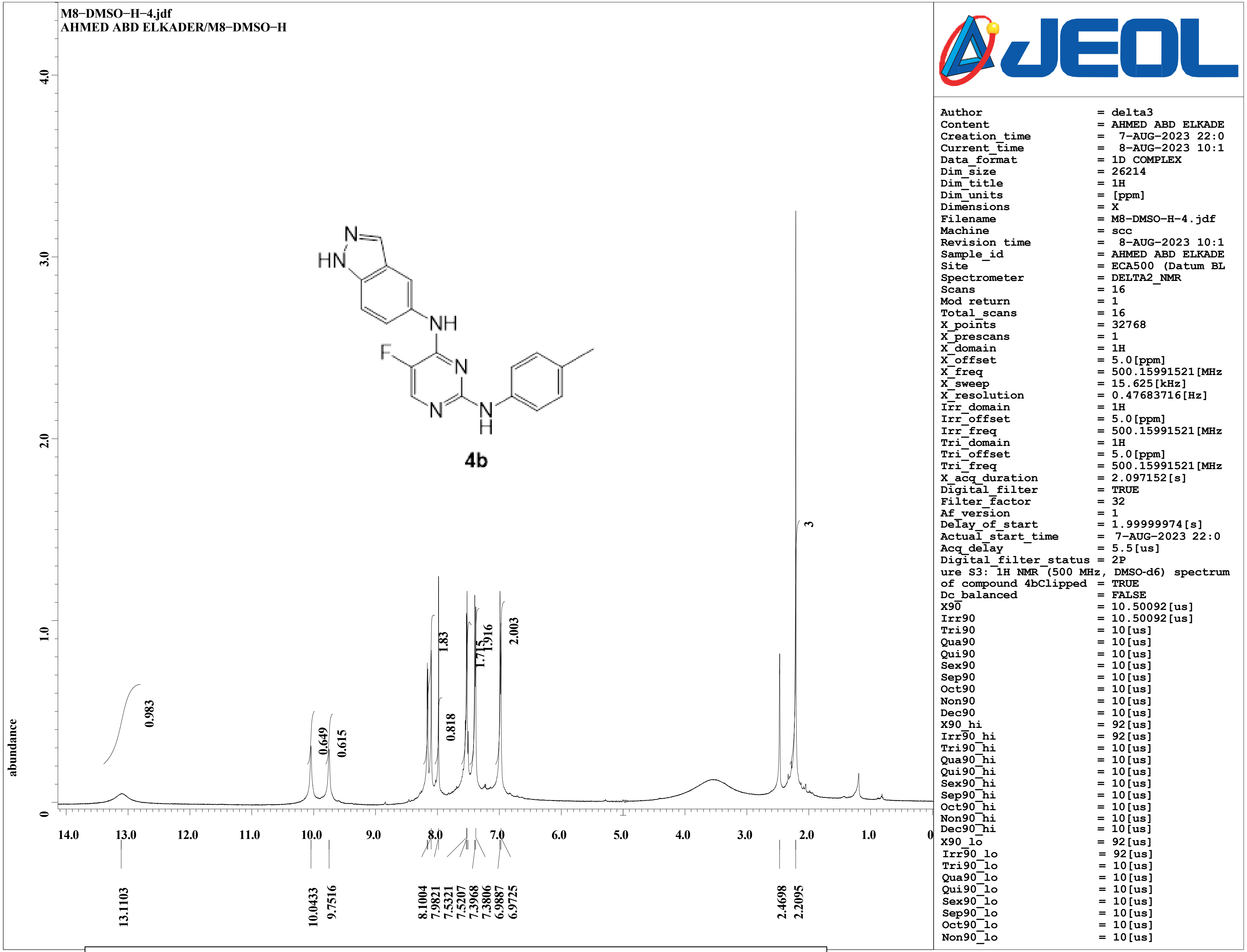

Figure S3: ^{1}H NMR (500 MHz, DMSO-d6) spectrum of compound **4b**

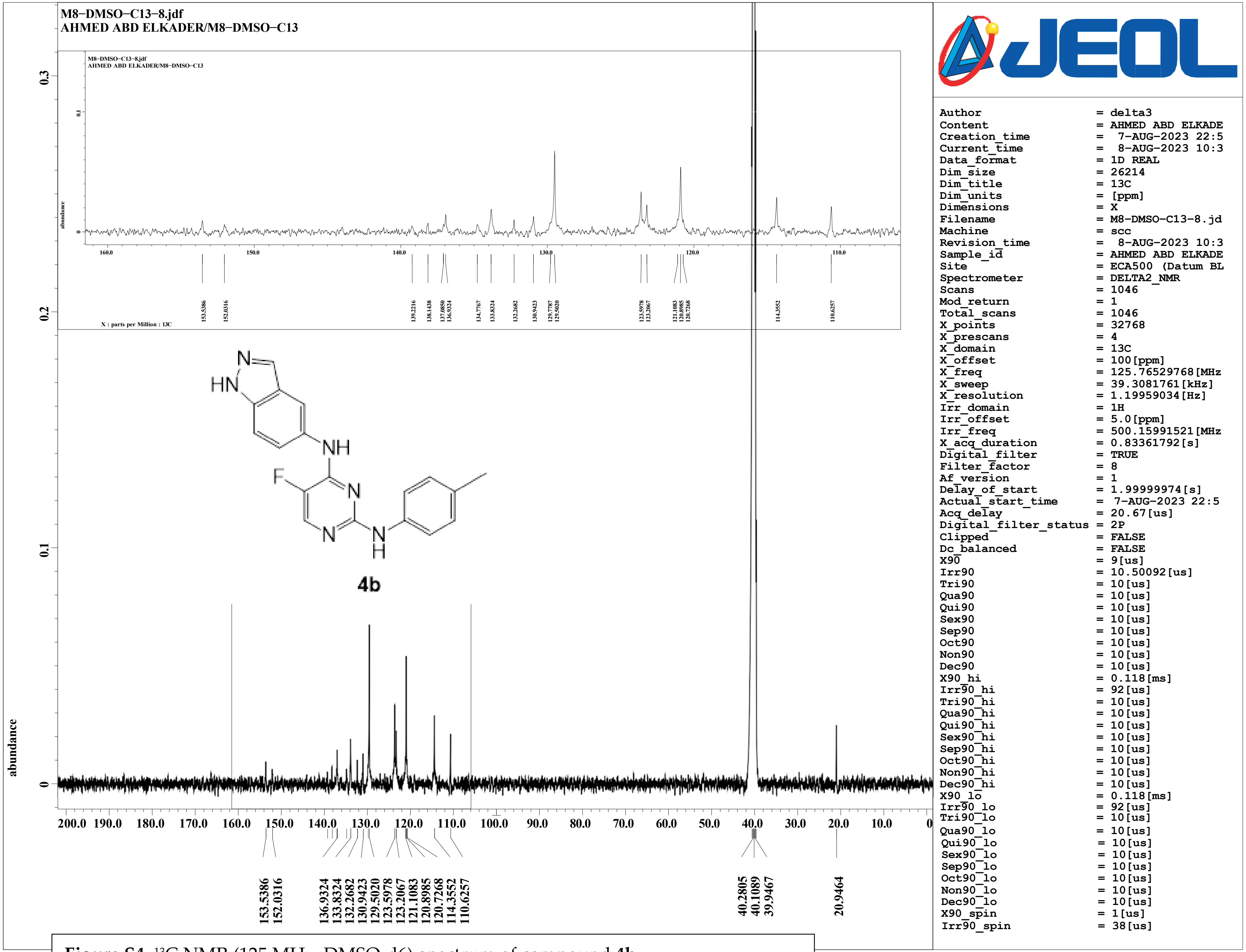

Figure S4: ^{13}C NMR (125 MHz, DMSO-d6) spectrum of compound **4b**

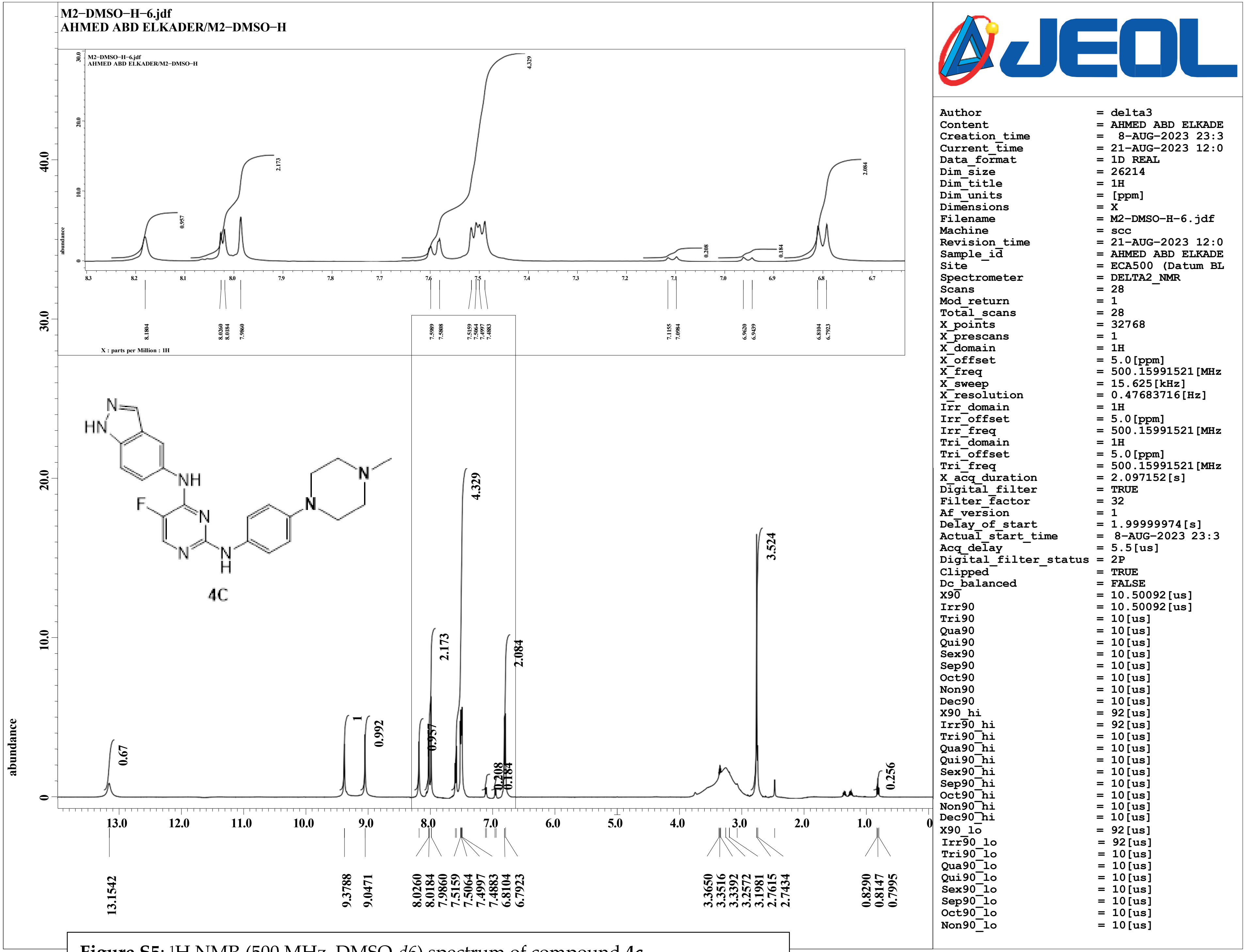

Figure S5: ^{1}H NMR (500 MHz, DMSO-*d6*) spectrum of compound **4c**

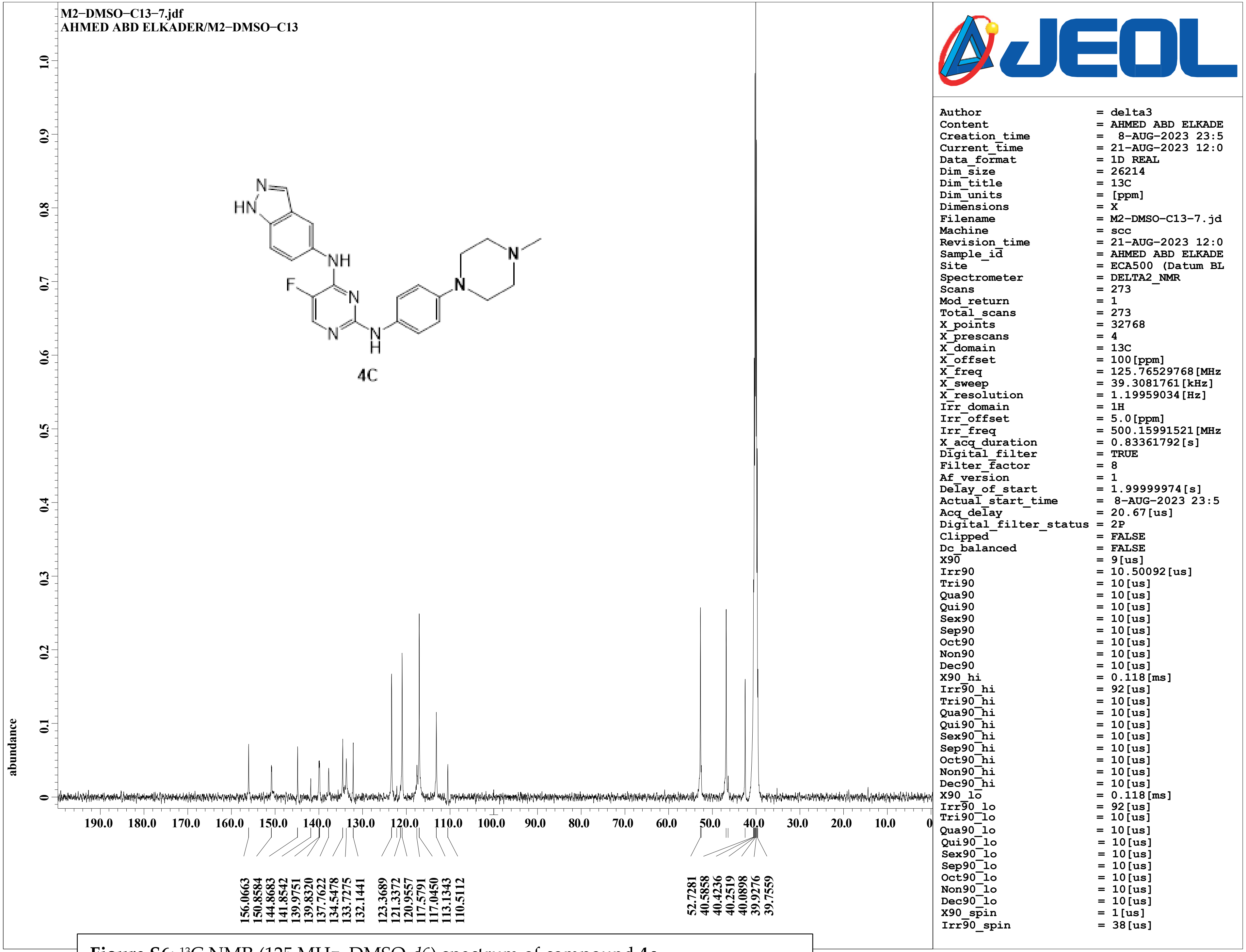

Figure S6: ^{13}C NMR (125 MHz, DMSO-*d6*) spectrum of compound **4c**

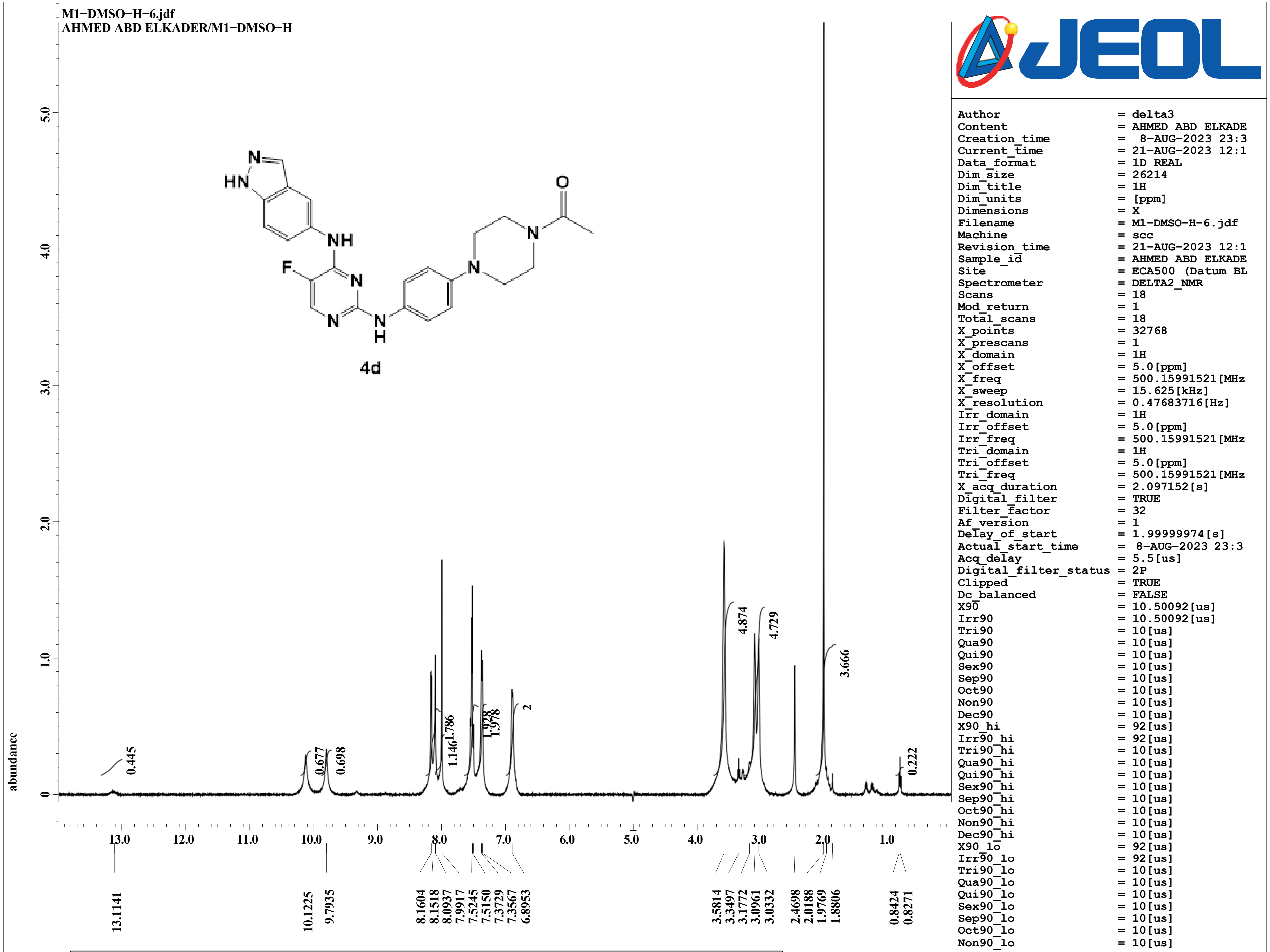

Figure S7: ^{1}H NMR (500 MHz, DMSO-*d6*) spectrum of compound **4d**

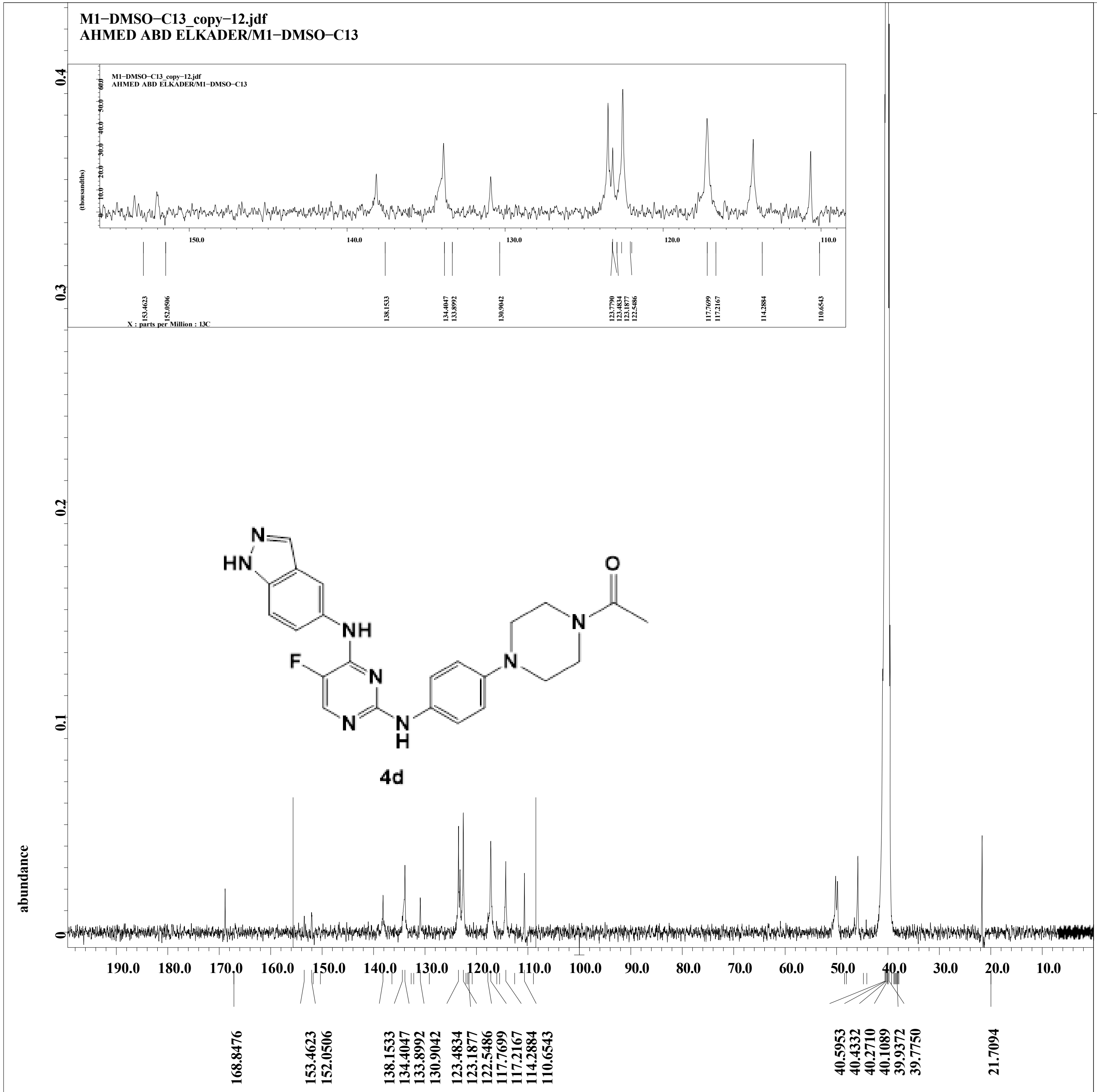

JEOL

Author	= delta3
Content	= AHMED ABD ELKADE
Creation_time	= 9-AUG-2023 00:3
Current_time	= 21-AUG-2023 12:2
Data_format	= 1D REAL
Dim_size	= 26214
Dim_title	= 13C
Dim_units	= [ppm]
Dimensions	= X
Filename	= M1-DMSO-C13_copy
Machine	= scc
Revision_time	= 21-AUG-2023 12:2
Sample_id	= AHMED ABD ELKADE
Site	= ECA500 (Datum BL
Spectrometer	= DELTA2_NMR
Scans	= 863
Mod_return	= 1
Total_scans	= 863
X_points	= 32768
X_prescans	= 4
X_domain	= 13C
X_offset	= 100[ppm]
X_freq	= 125.76529768[MHz
X_sweep	= 39.3081761[kHz]
X_resolution	= 1.19959034[Hz]
Irr_domain	= 1H
Irr_offset	= 5.0[ppm]
Irr_freq	= 500.15991521[MHz
X_acq_duration	= 0.83361792[s]
Digital_filter	= TRUE
Filter_factor	= 8
Af_version	= 1
Delay_of_start	= 1.99999974[s]
Actual_start_time	= 9-AUG-2023 00:3
Acq_delay	= 20.67[us]
Digital_filter_status	= 2P
Clipped	= FALSE
Dc_balanced	= FALSE
X90	= 9[us]
Irr90	= 10.50092[us]
Tri90	= 10[us]
Qua90	= 10[us]
Qui90	= 10[us]
Sex90	= 10[us]
Sep90	= 10[us]
Oct90	= 10[us]
Non90	= 10[us]
Dec90	= 10[us]
X90_hi	= 0.118[ms]
Irr90_hi	= 92[us]
Tri90_hi	= 10[us]
Qua90_hi	= 10[us]
Qui90_hi	= 10[us]
Sex90_hi	= 10[us]
Sep90_hi	= 10[us]
Oct90_hi	= 10[us]
Non90_hi	= 10[us]
Dec90_hi	= 10[us]
X90_lo	= 0.118[ms]
Irr90_lo	= 92[us]
Tri90_lo	= 10[us]
Qua90_lo	= 10[us]
Qui90_lo	= 10[us]
Sex90_lo	= 10[us]
Sep90_lo	= 10[us]
Oct90_lo	= 10[us]
Non90_lo	= 10[us]
Dec90_lo	= 10[us]
X90_spin	= 1[us]
Irr90_spin	= 38[us]

Figure S8: ^{13}C NMR (125 MHz, DMSO-*d6*) spectrum of compound **4d**

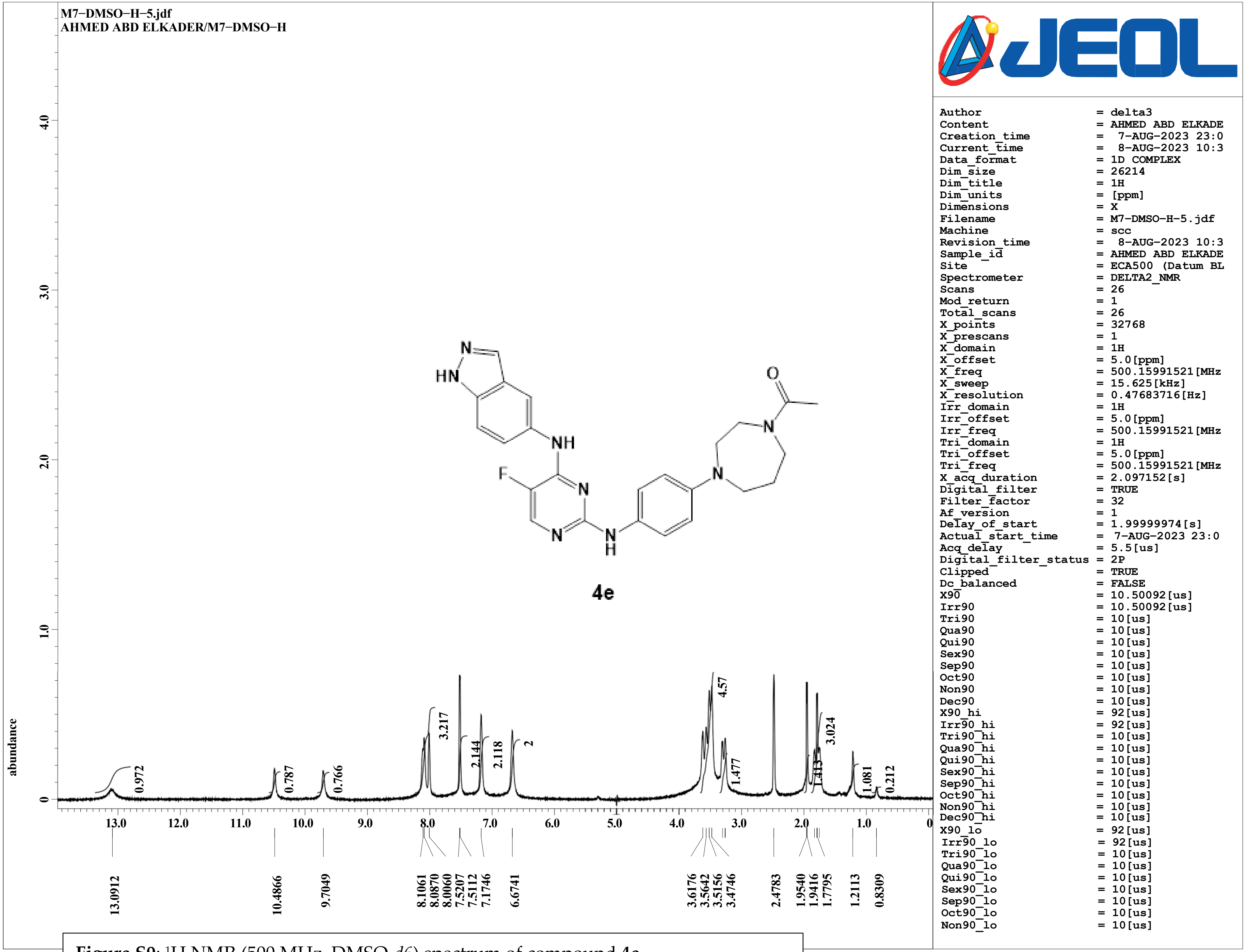

Figure S9: ^{1}H NMR (500 MHz, DMSO-*d6*) spectrum of compound **4e**

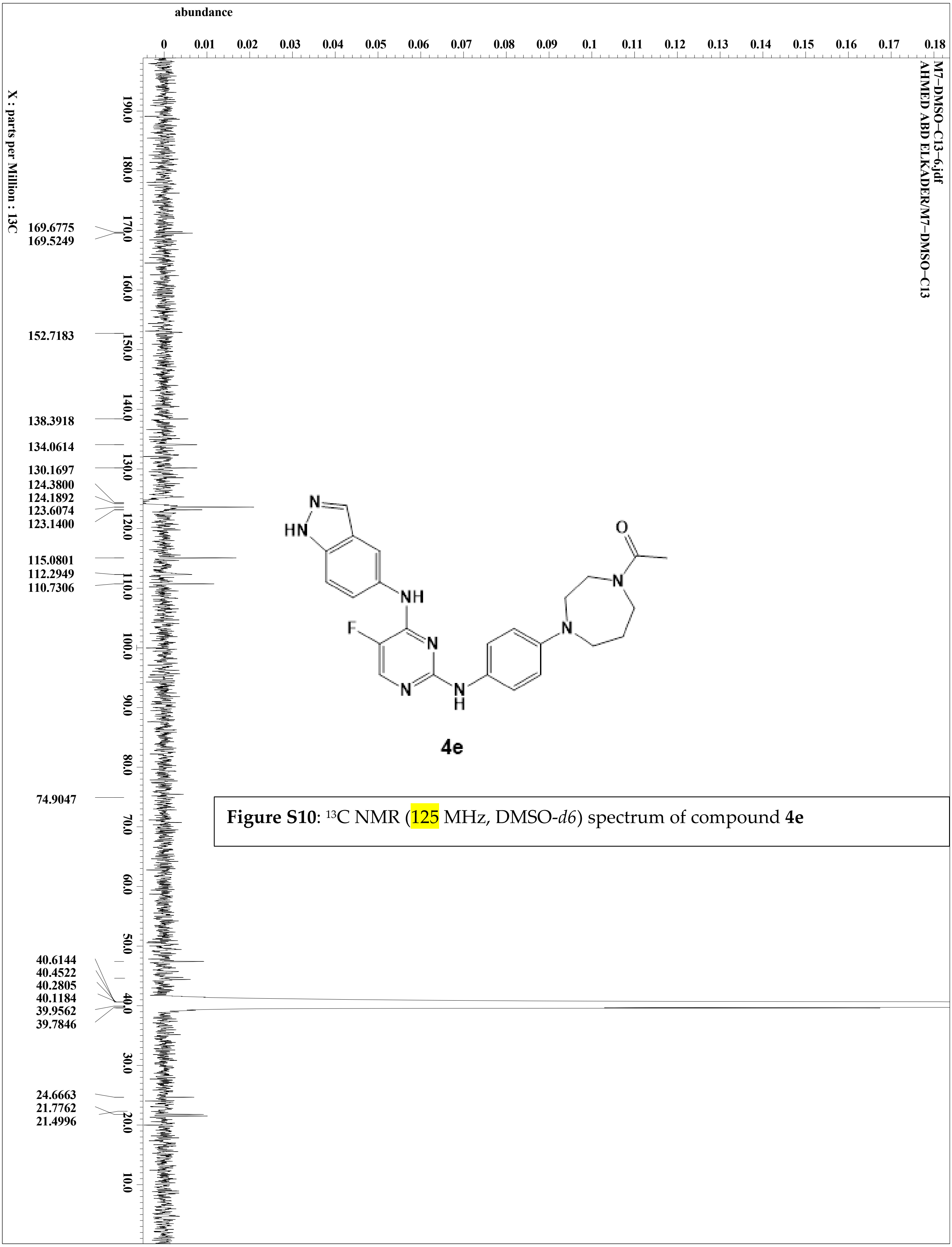

Figure S10: ^{13}C NMR (125 MHz, DMSO-*d6*) spectrum of compound **4e**

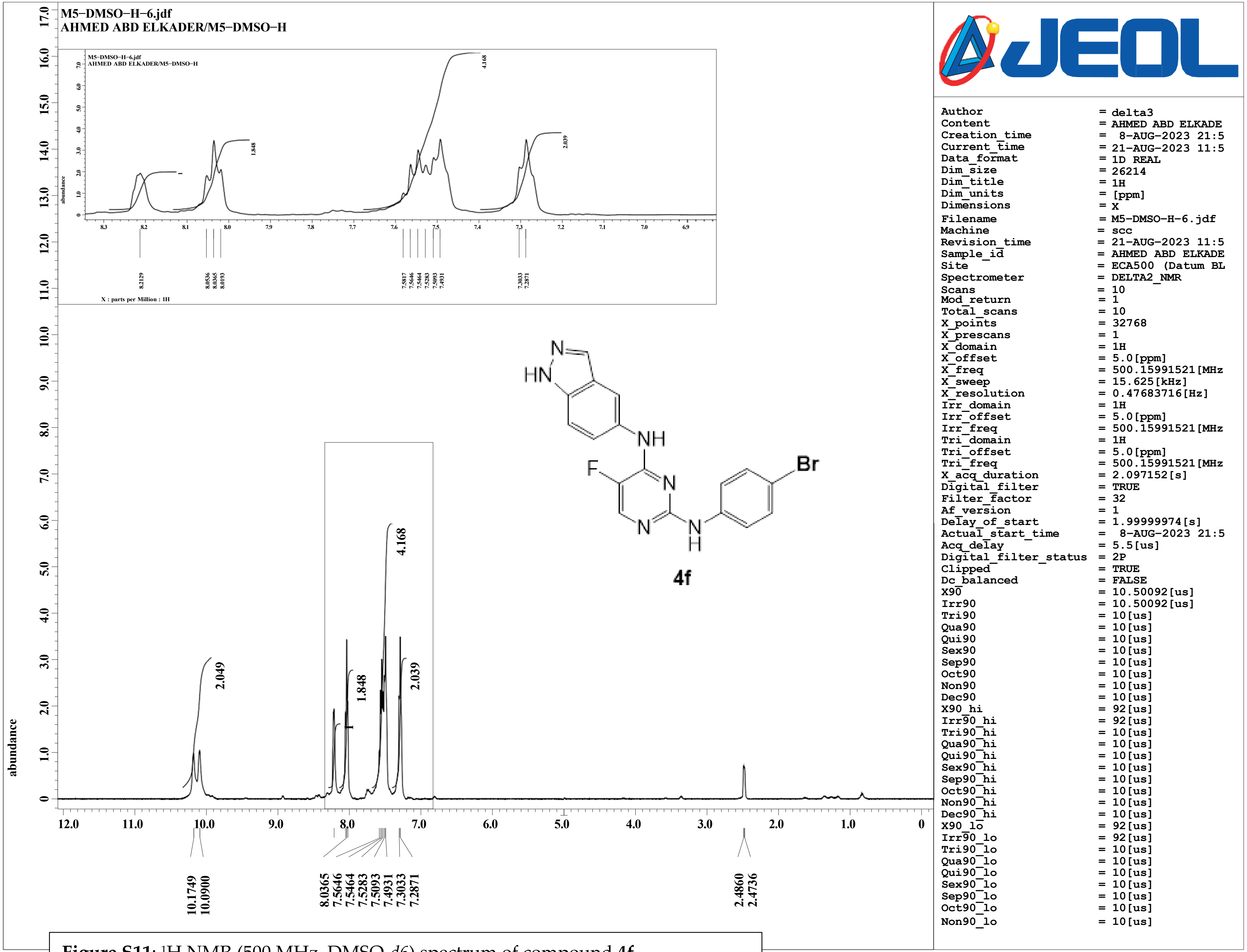

Figure S11: ^{1}H NMR (500 MHz, DMSO-*d6*) spectrum of compound **4f**

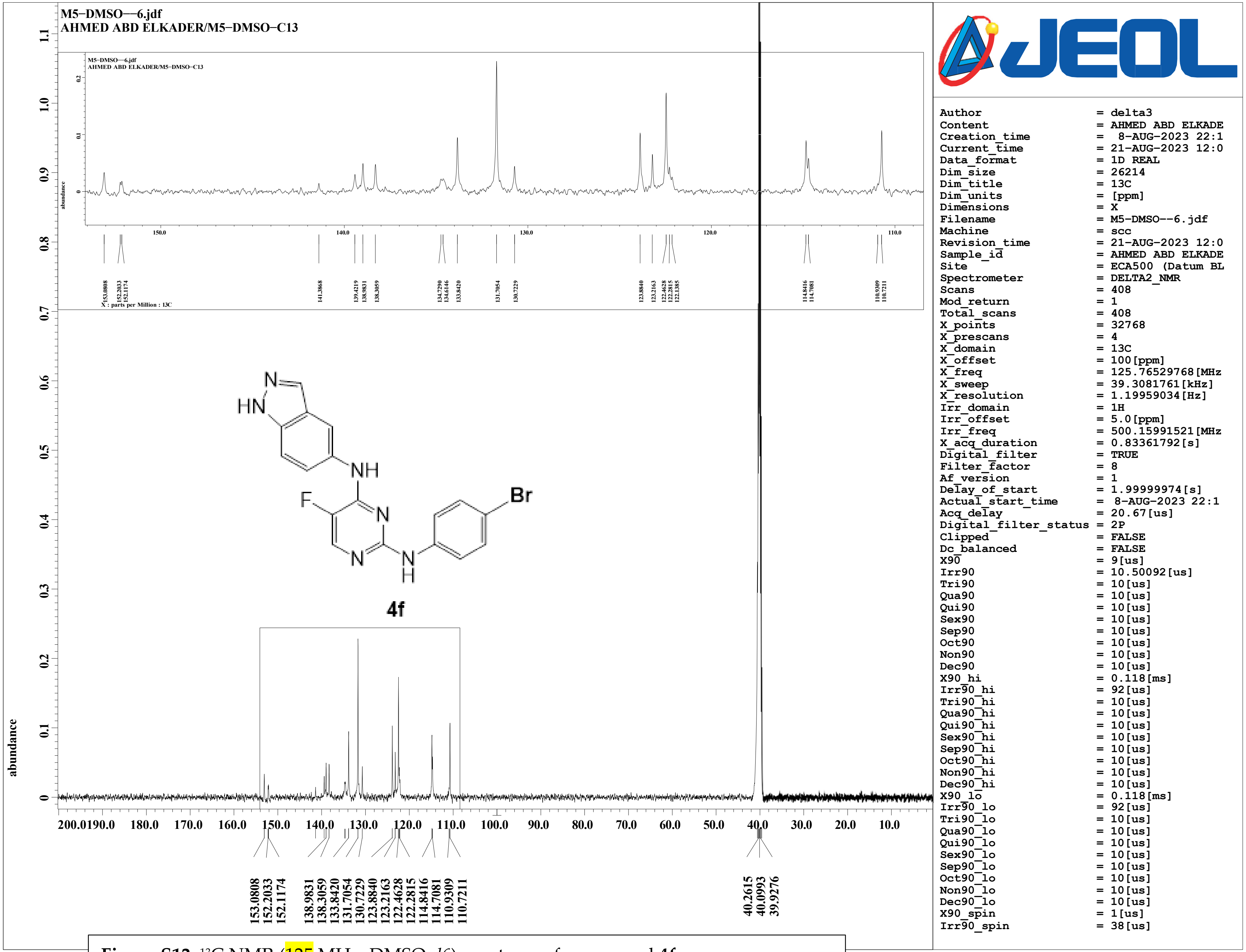

Figure S12: ^{13}C NMR (125 MHz, DMSO-*d6*) spectrum of compound **4f**

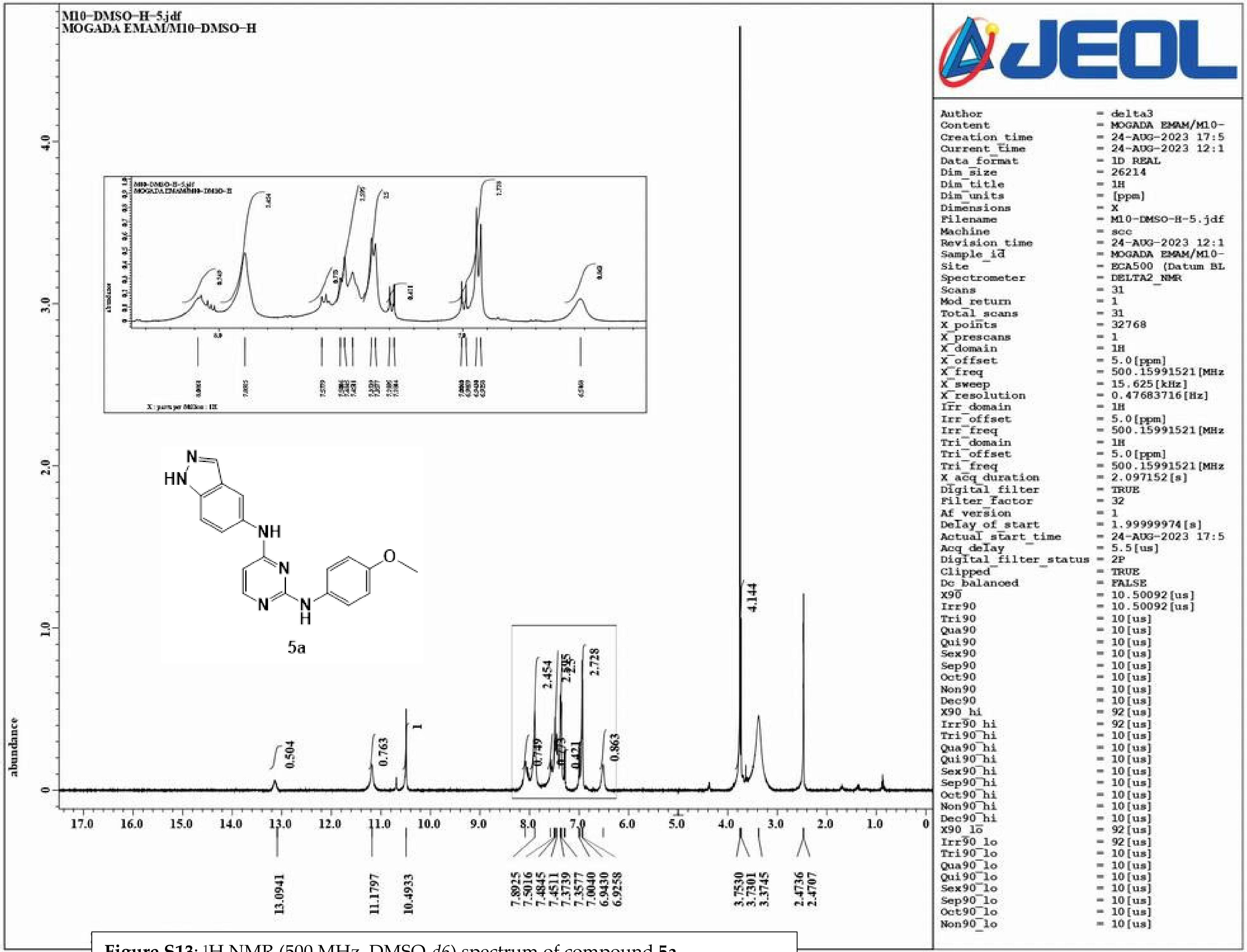

Figure S13: ^{1}H NMR (500 MHz, DMSO-*d6*) spectrum of compound **5a**

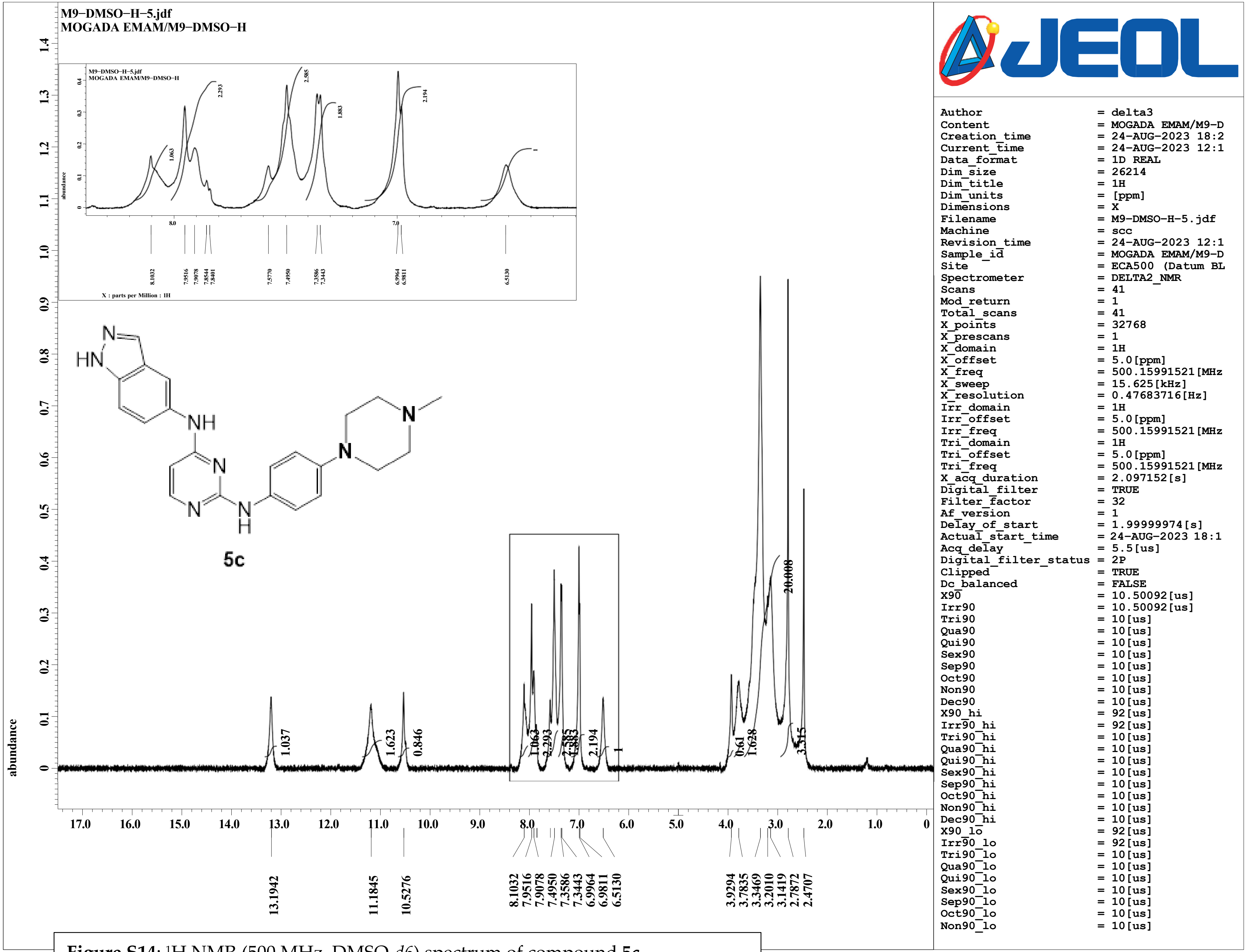

Figure S14: ^{1}H NMR (500 MHz, DMSO-*d6*) spectrum of compound **5c**

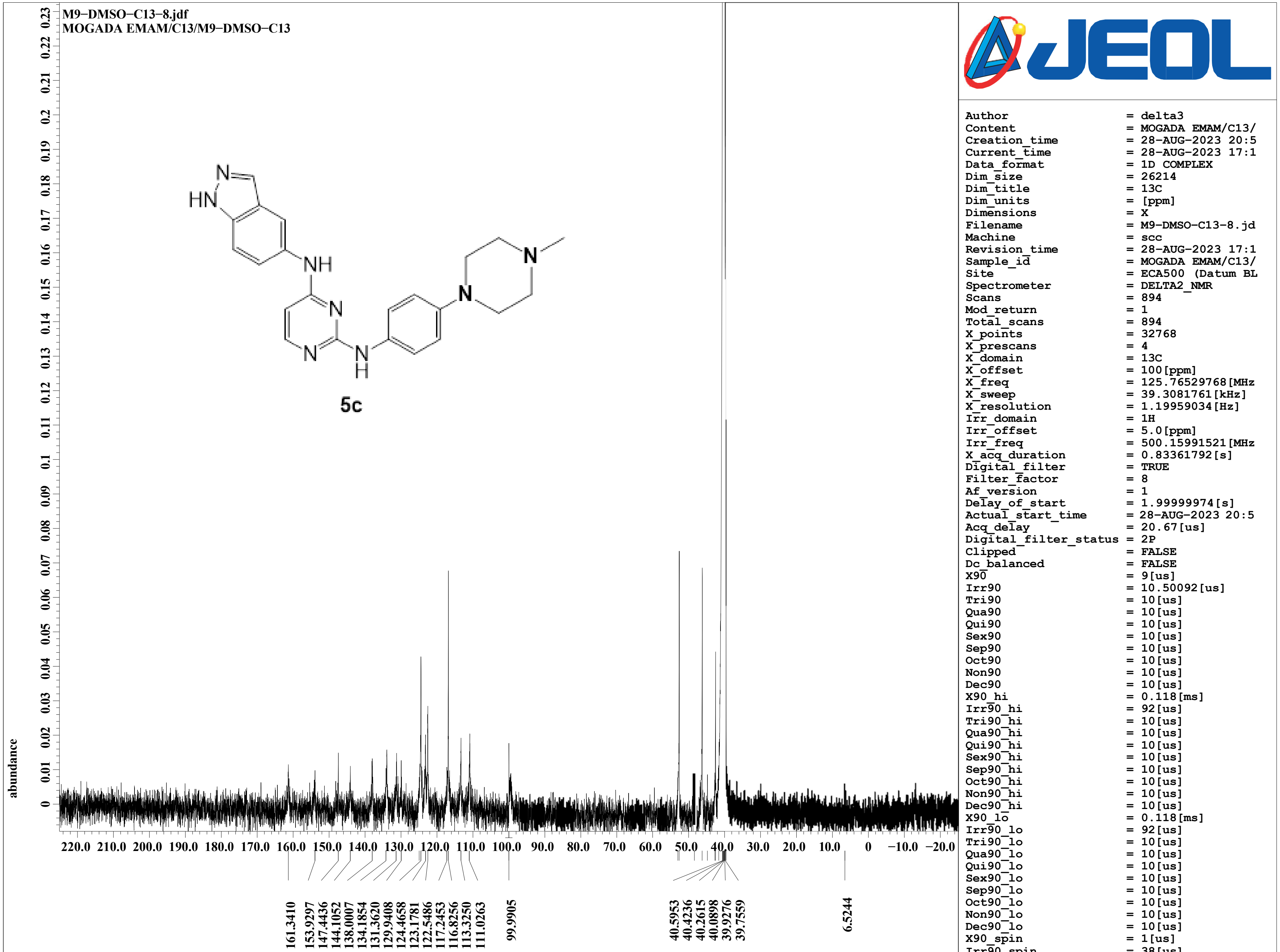

Figure S15: ^{13}C NMR (125 MHz, DMSO-*d6*) spectrum of compound **5c**

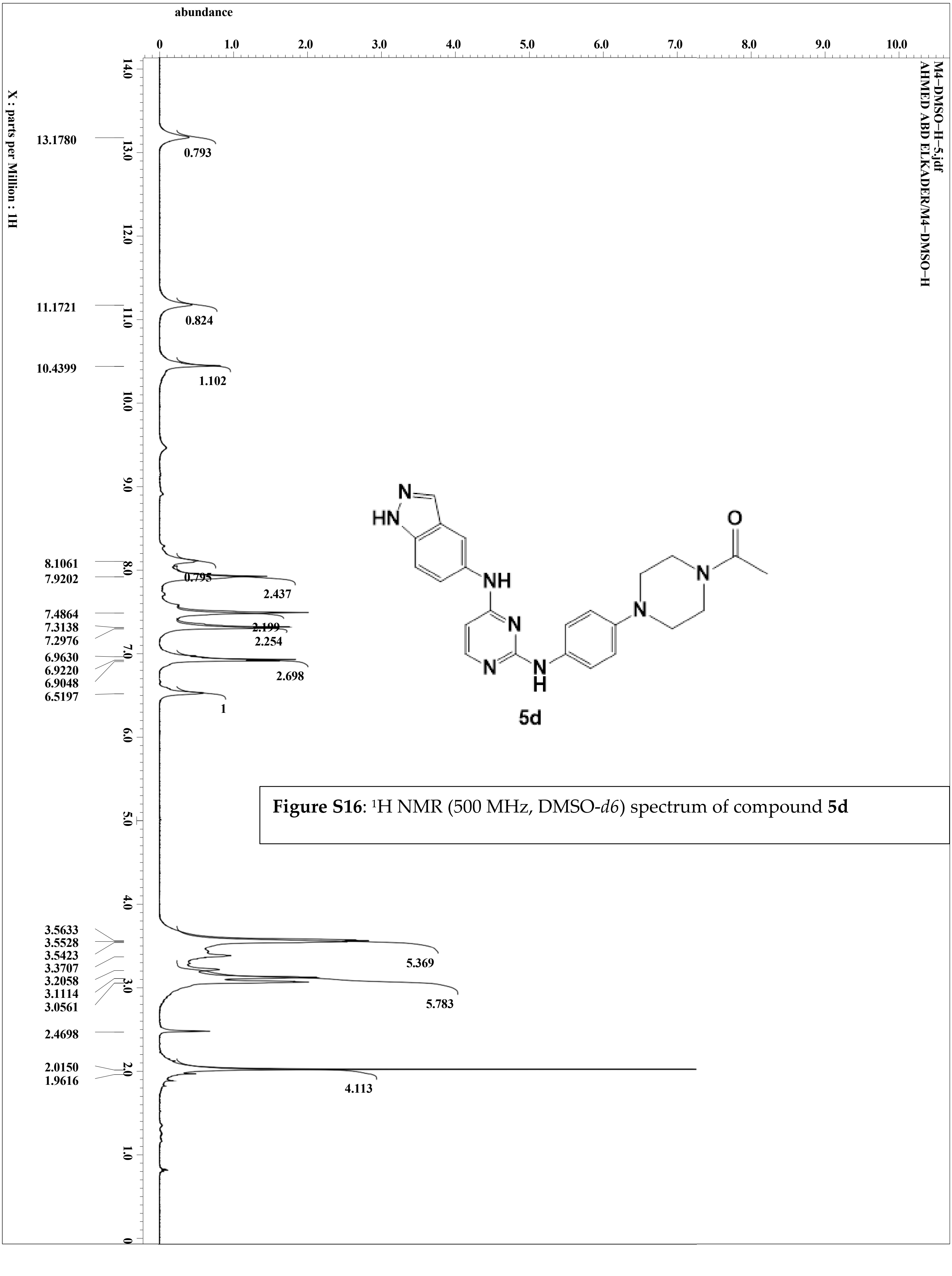

Figure S16: ^{1}H NMR (500 MHz, DMSO-*d6*) spectrum of compound **5d**

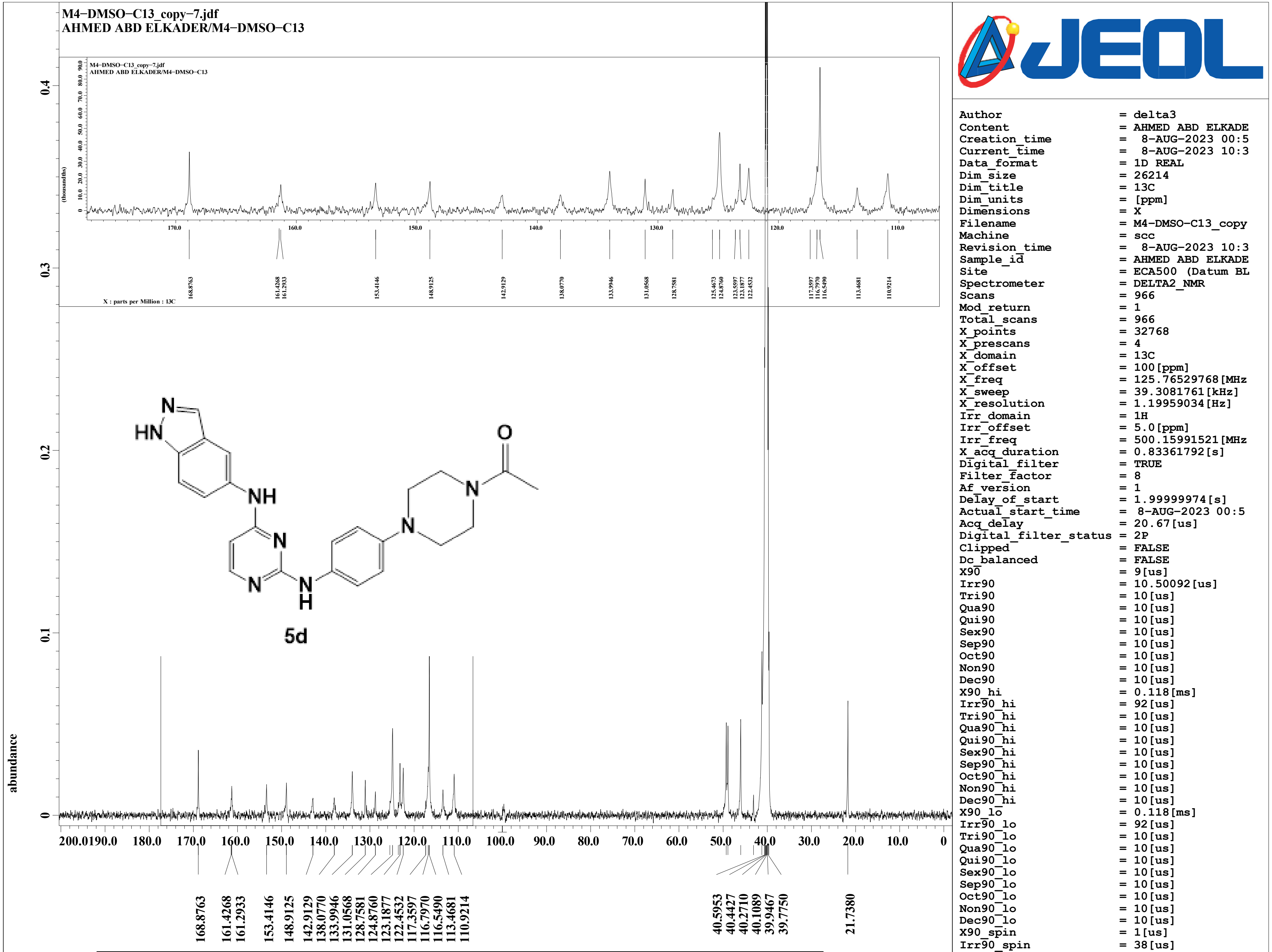

Figure S17: ^{13}C NMR (125 MHz, DMSO-*d6*) spectrum of compound **5d**

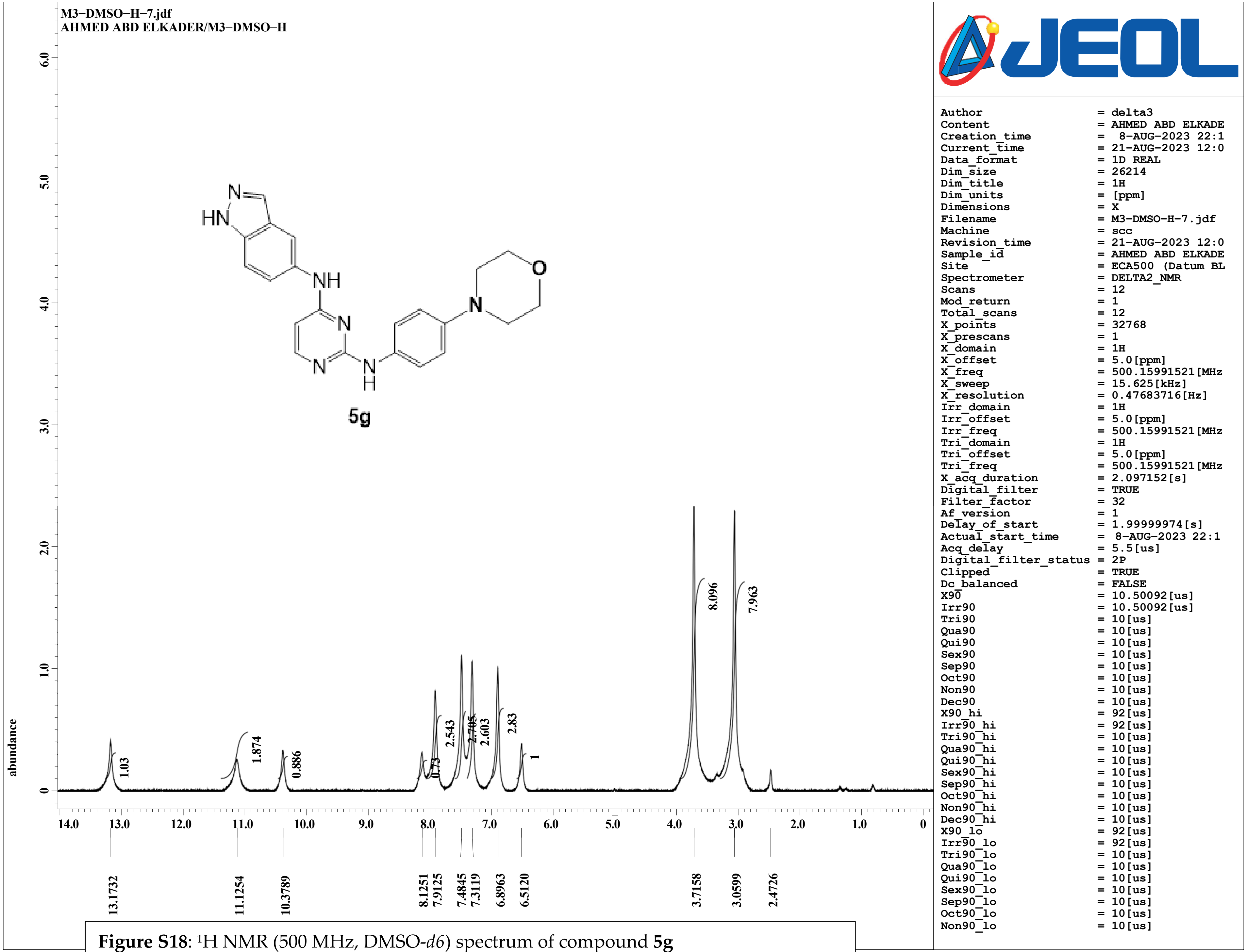

Figure S18: ^{1}H NMR (500 MHz, DMSO-*d6*) spectrum of compound **5g**

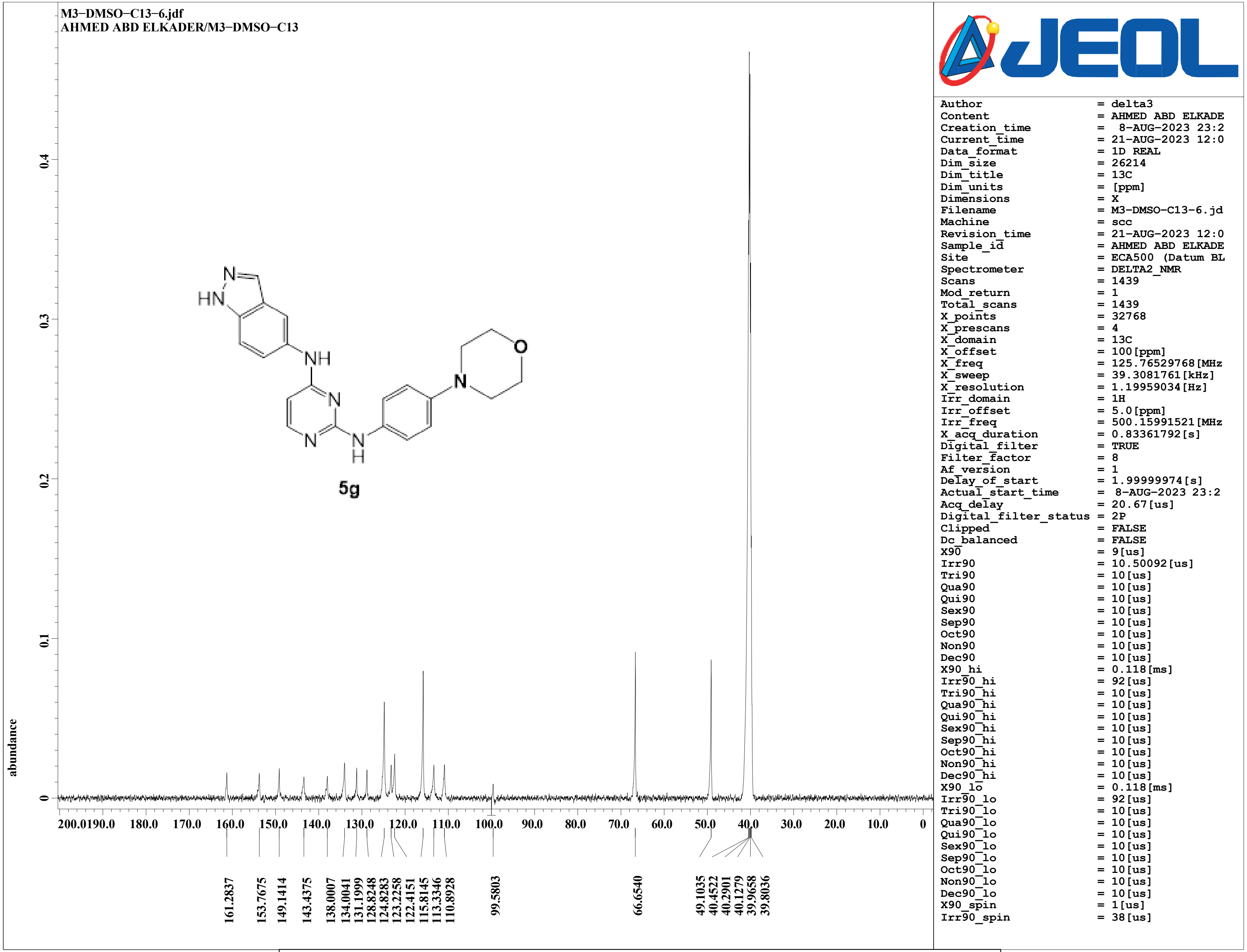

Figure S19: ^{13}C NMR (125 MHz, DMSO-d6) spectrum of compound **5g**

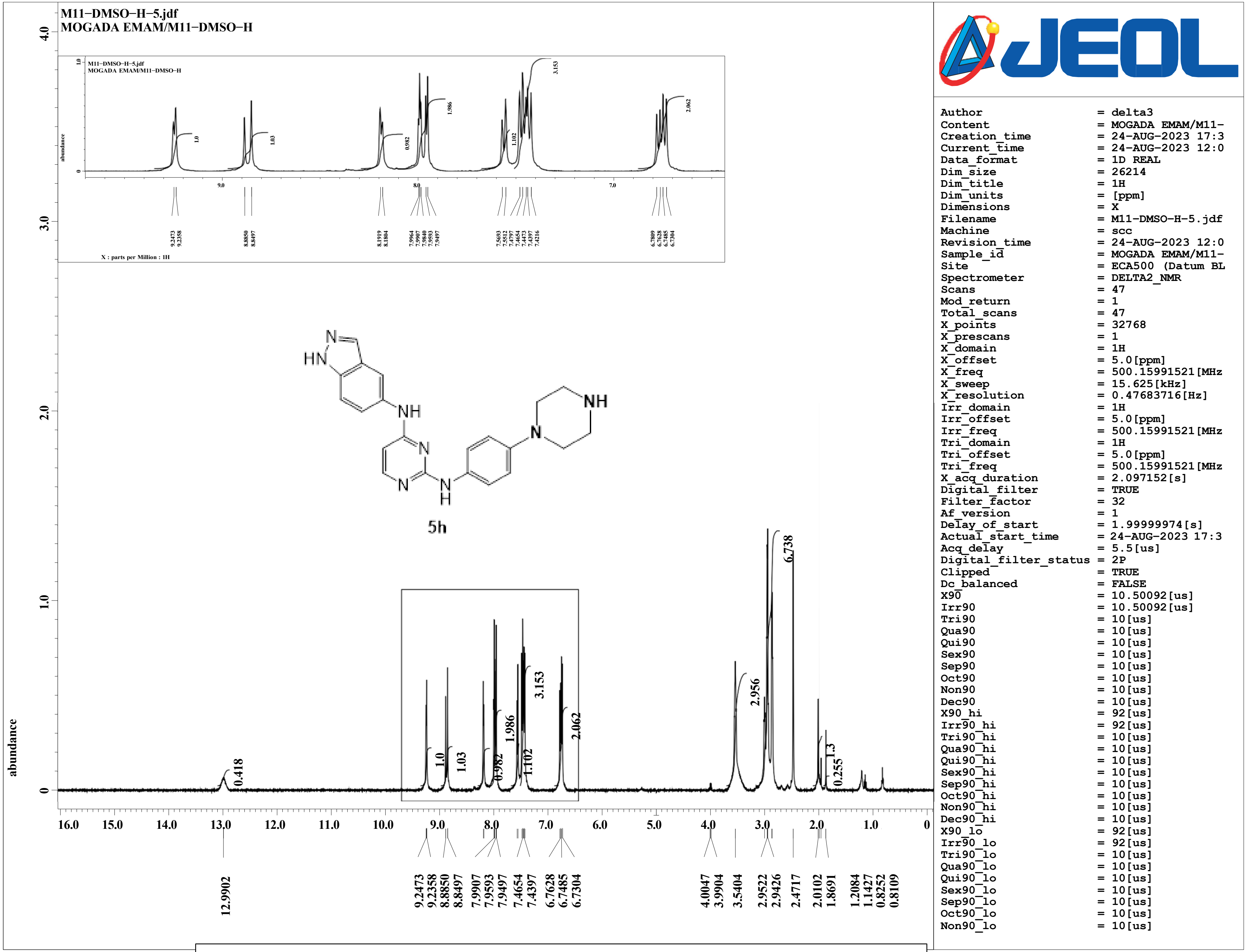

Figure S20: ^{1}H NMR (500 MHz, DMSO-*d6*) spectrum of compound **5h**

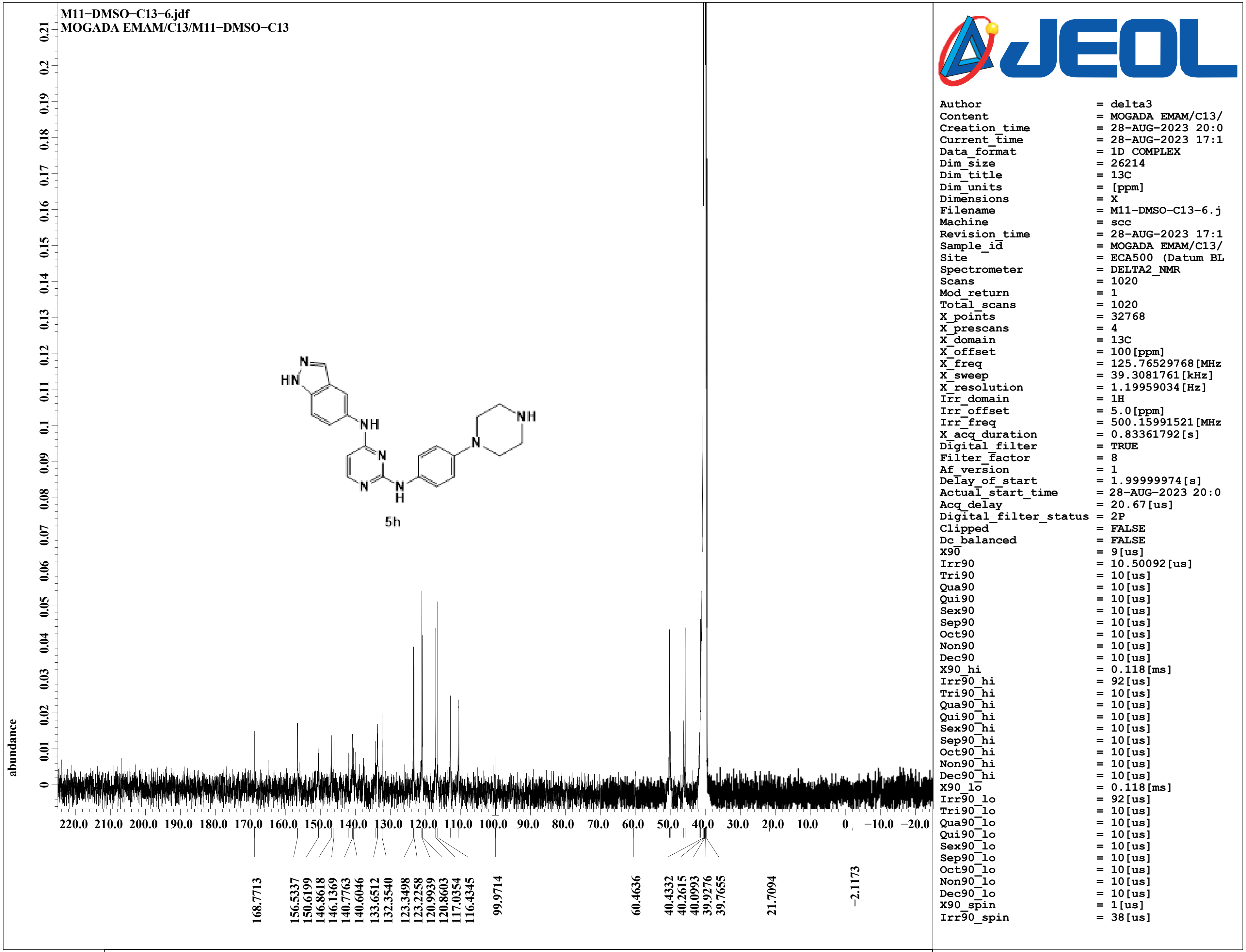

Figure S21: ^{13}C NMR (125 MHz, DMSO-*d6*) spectrum of compound **5h**

Supplementary Material

Indazol-Pyrimidine Hybrids: Design, Synthesis, and Antiproliferative Activity Against Human Cancer Cell Lines

Contents: IR spectra

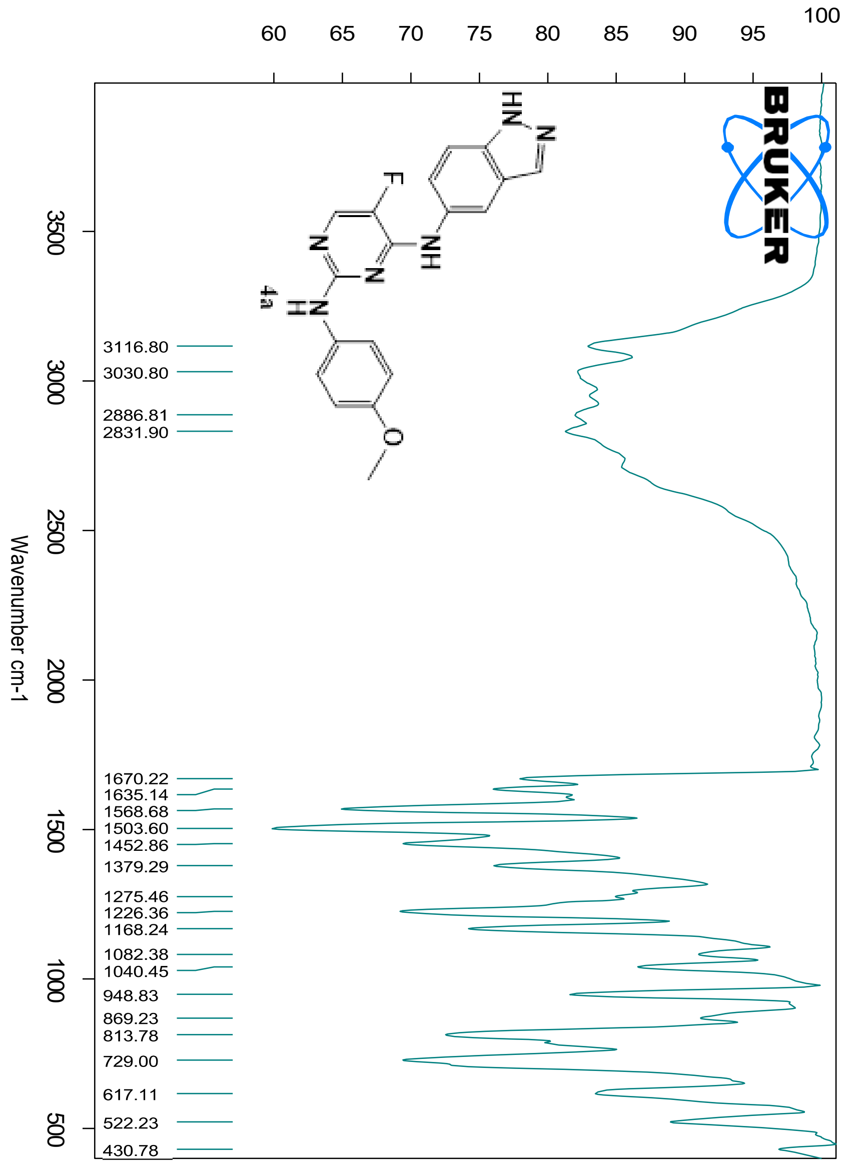

Figure S22: IR spectrum of compound **4a**

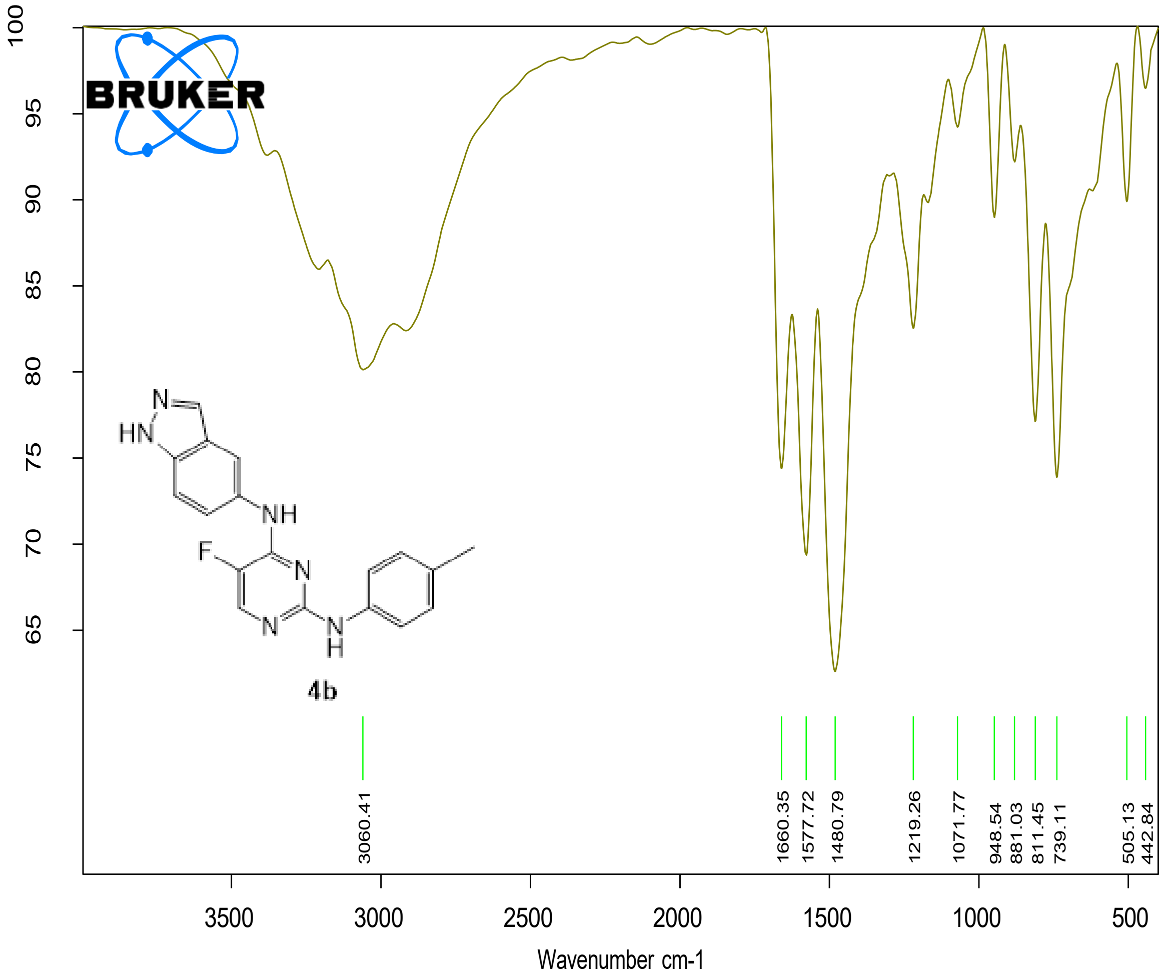

Figure S23:IR spectrum of compound **4b**

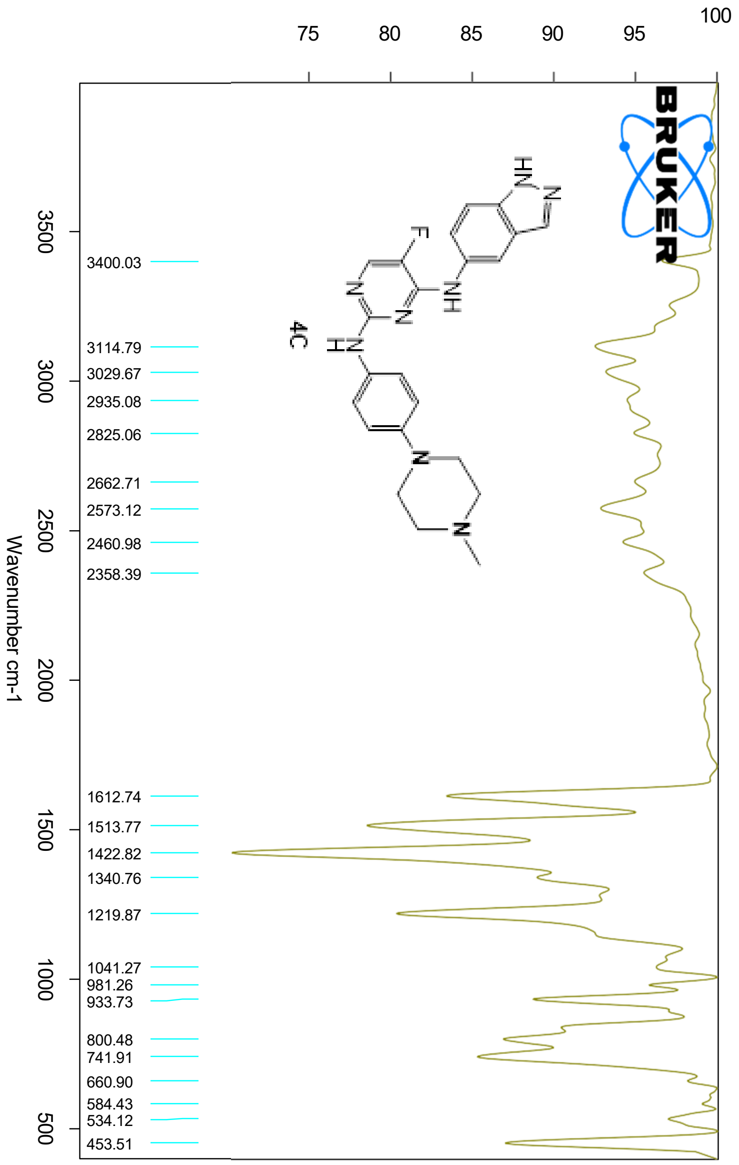

Figure S24: IR spectrum of compound **4c**

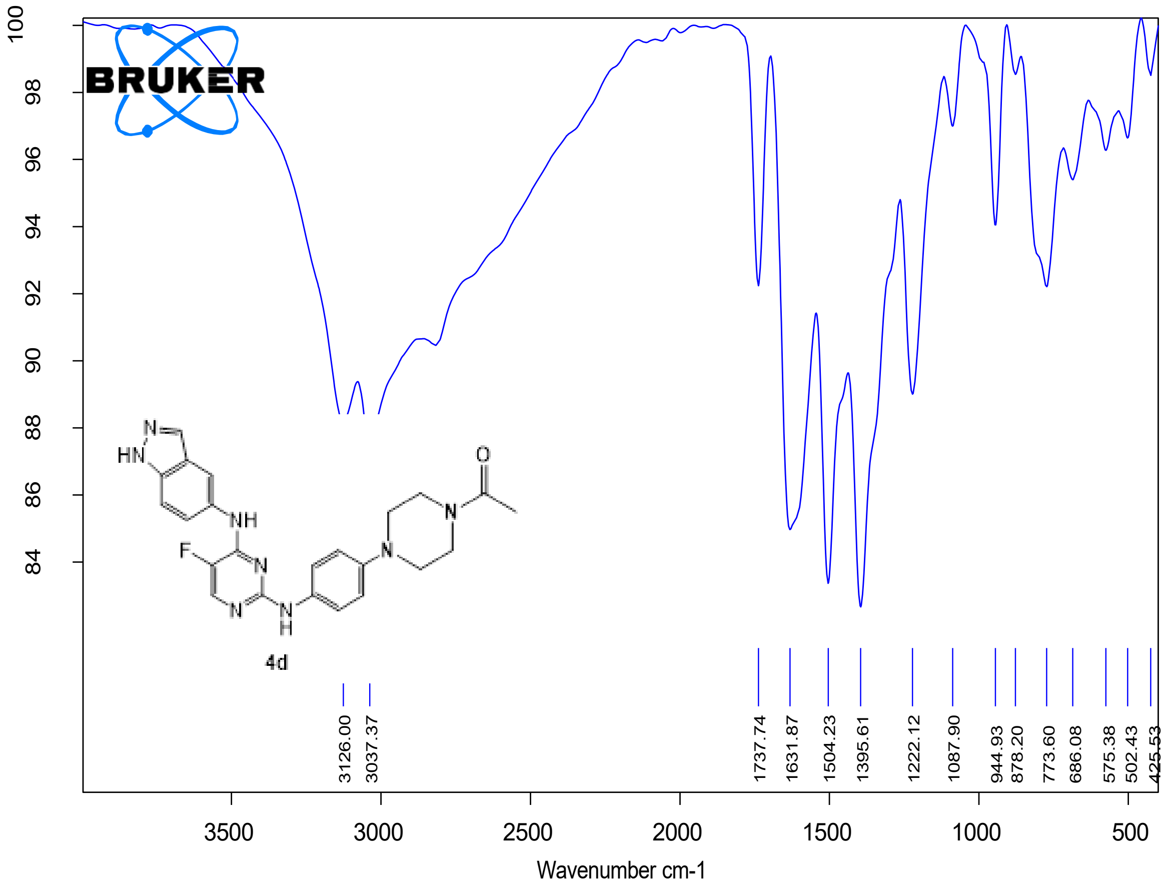

Figure S25: IR spectrum of compound **4d**

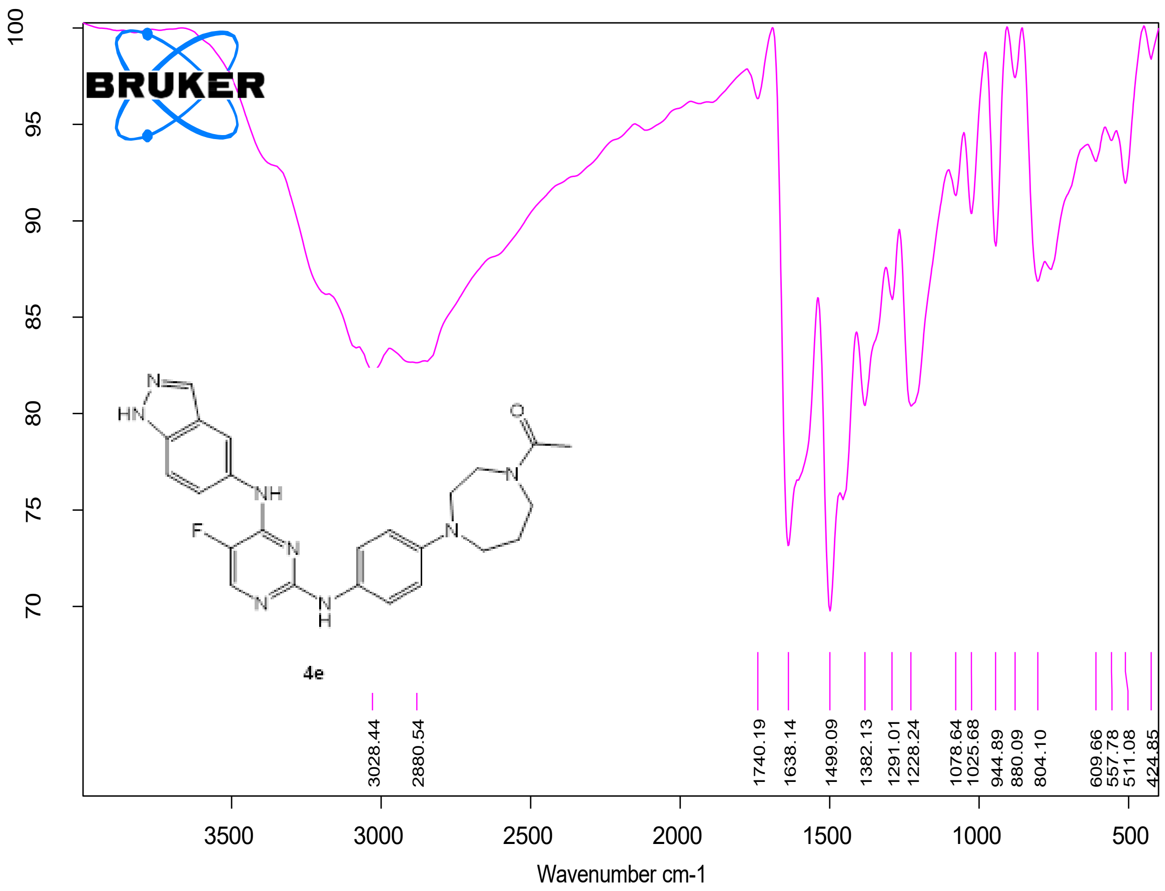

Figure S26: IR spectrum of compound **4e**

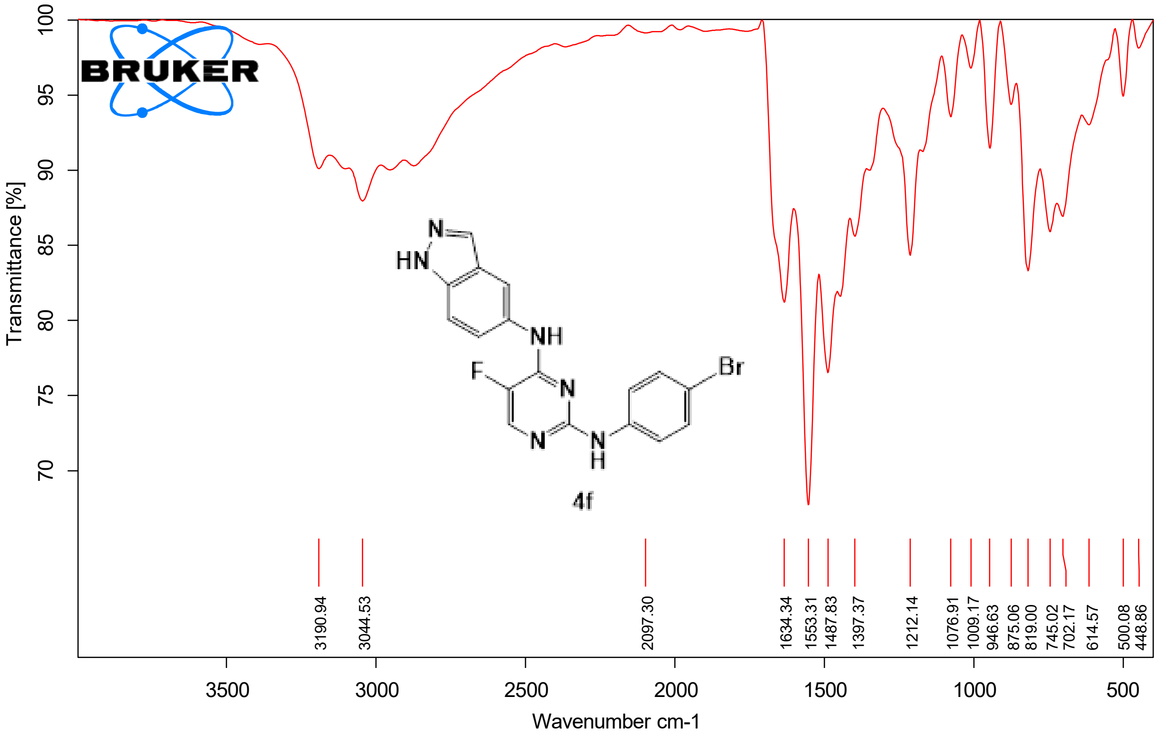

Figure S27: IR spectrum of compound **4f**

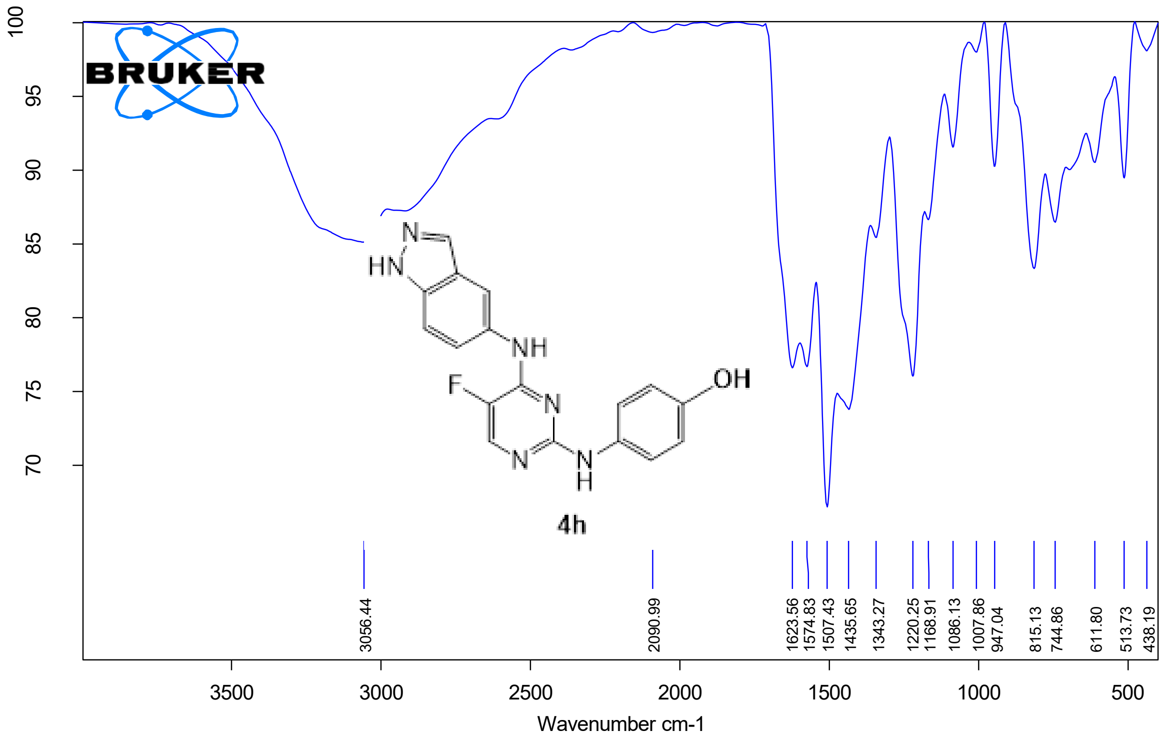

Figure S28: IR spectrum of compound **4h**

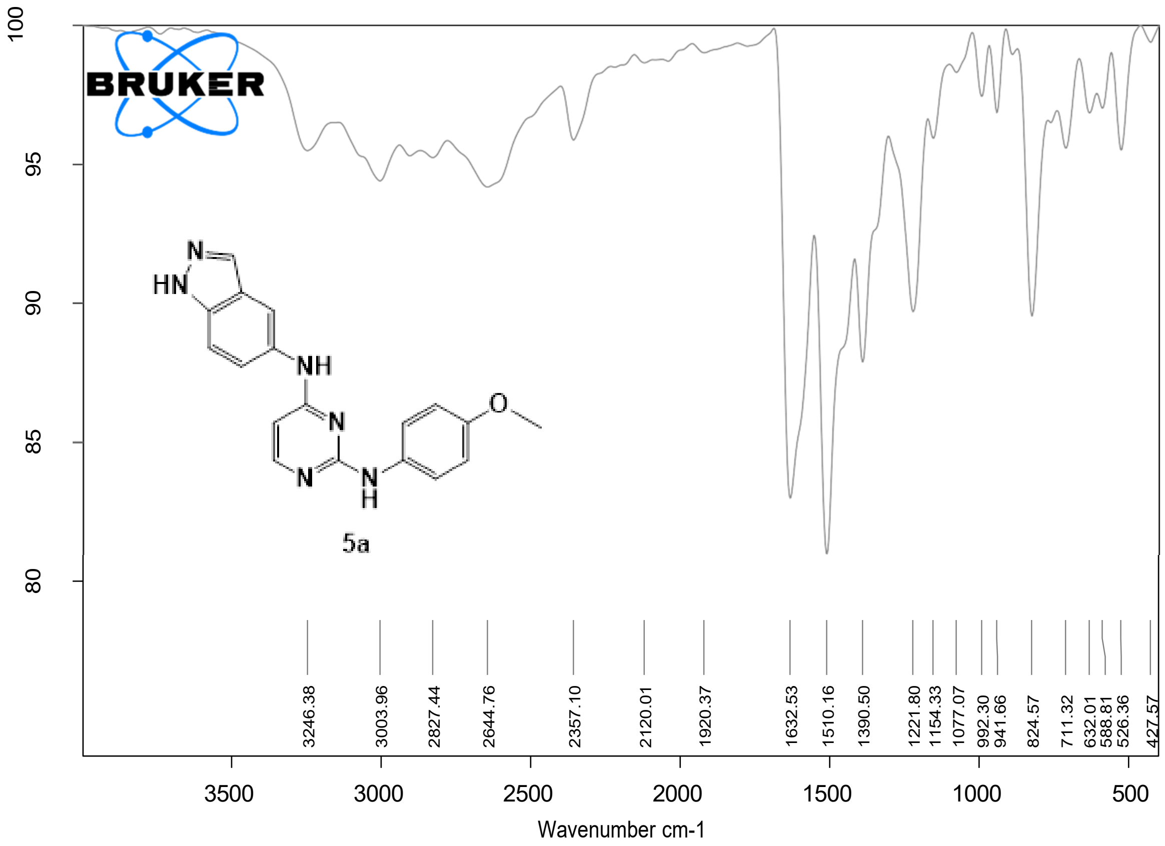

Figure S29: IR spectrum of compound **5a**

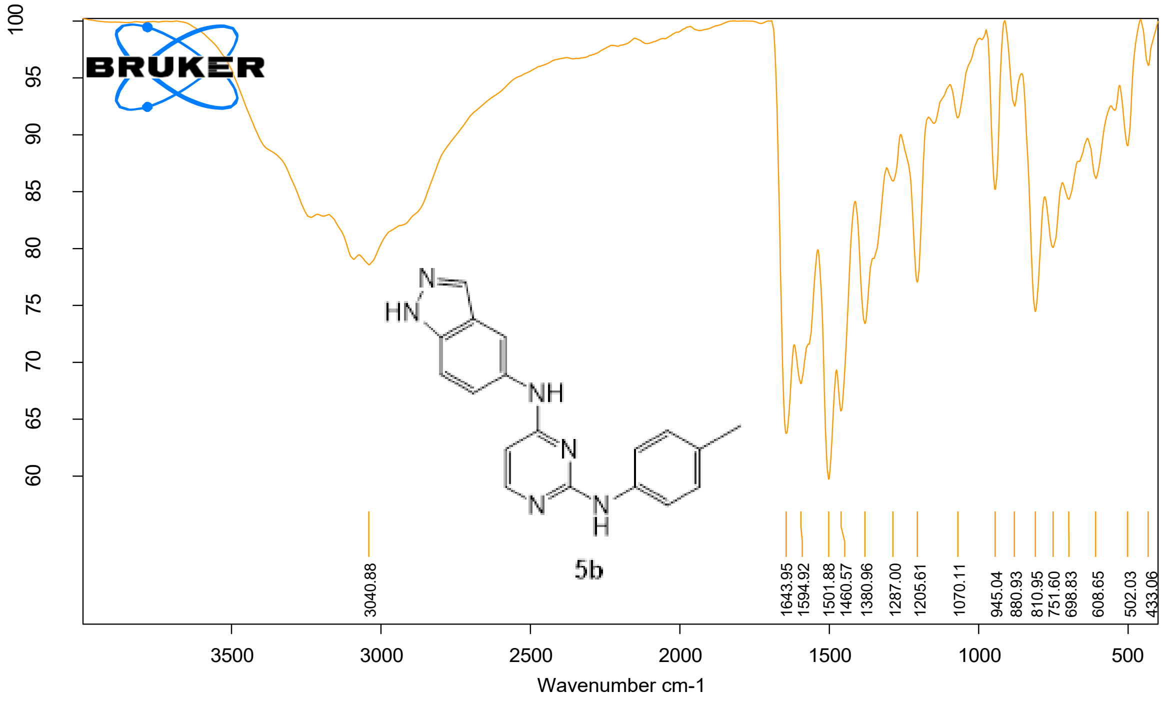

Figure S30: IR spectrum of compound **5b**

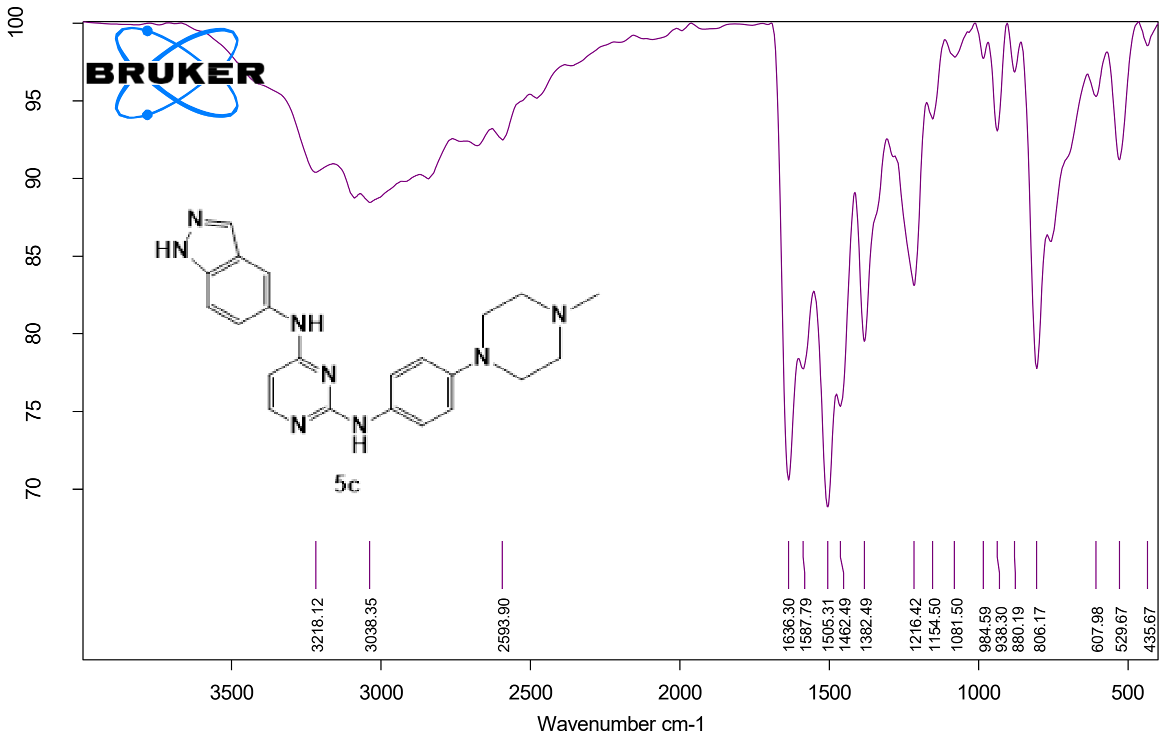

Figure S31: IR spectrum of compound **5c**

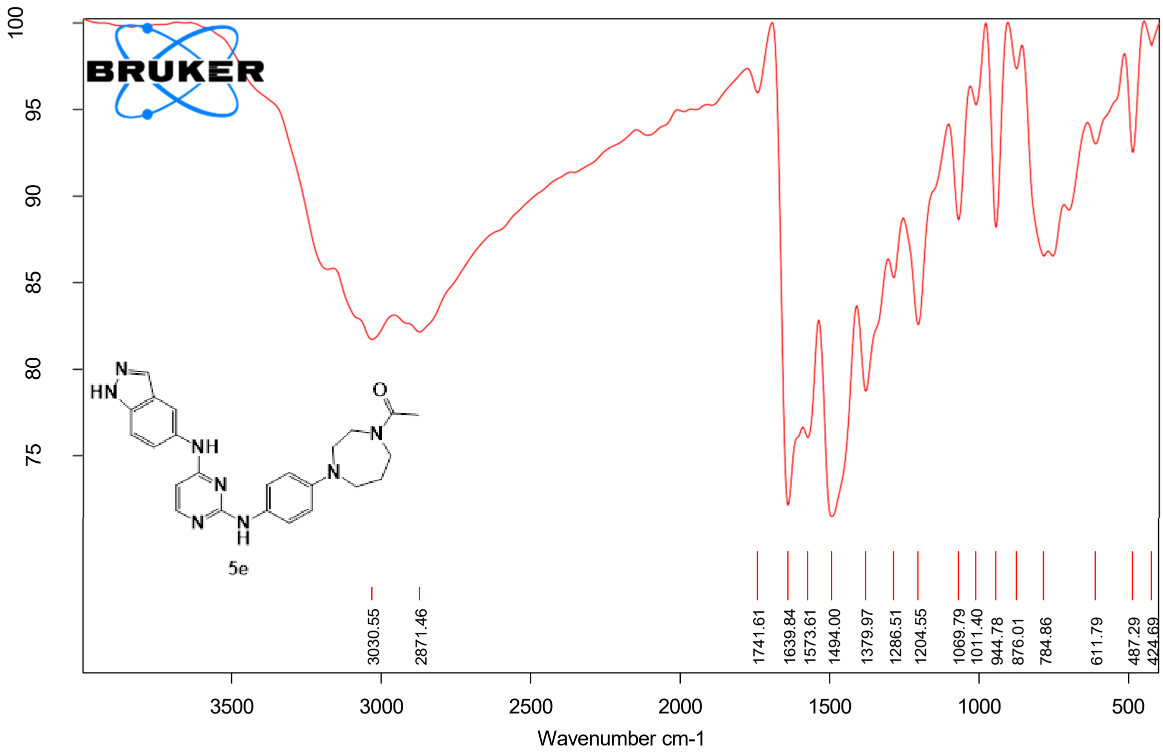

Figure S32: IR spectrum of compound **5e**

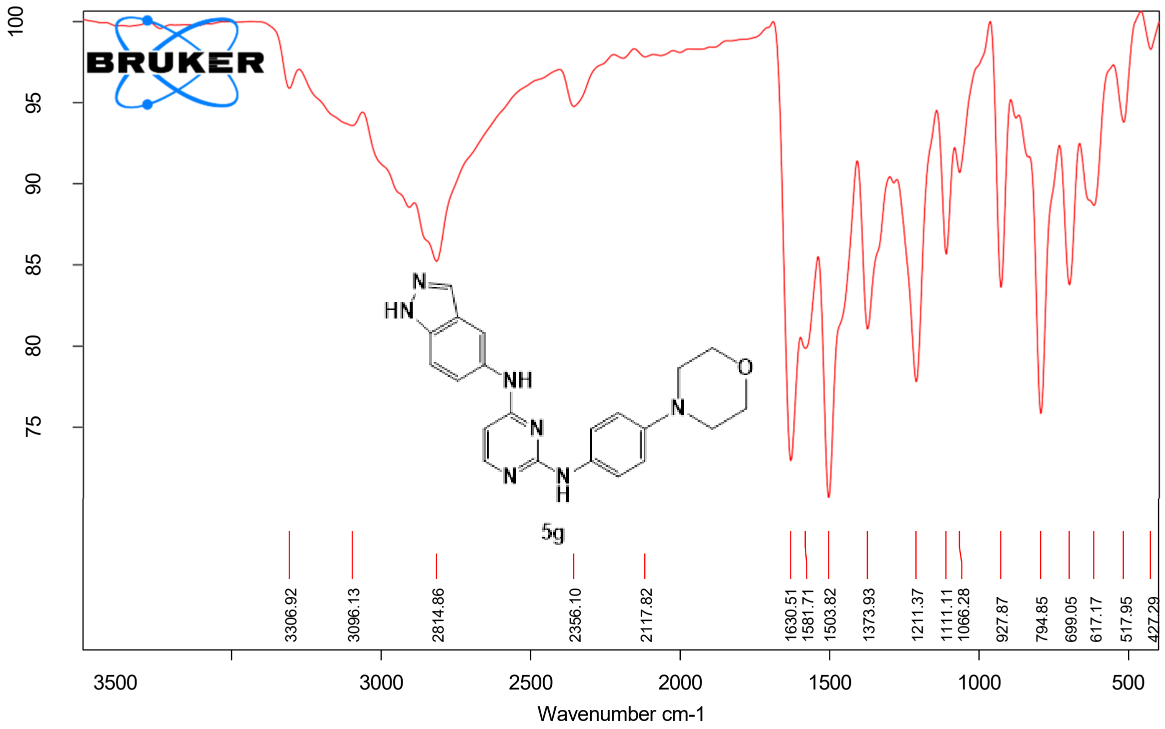

Figure S33: IR spectrum of compound **5g**

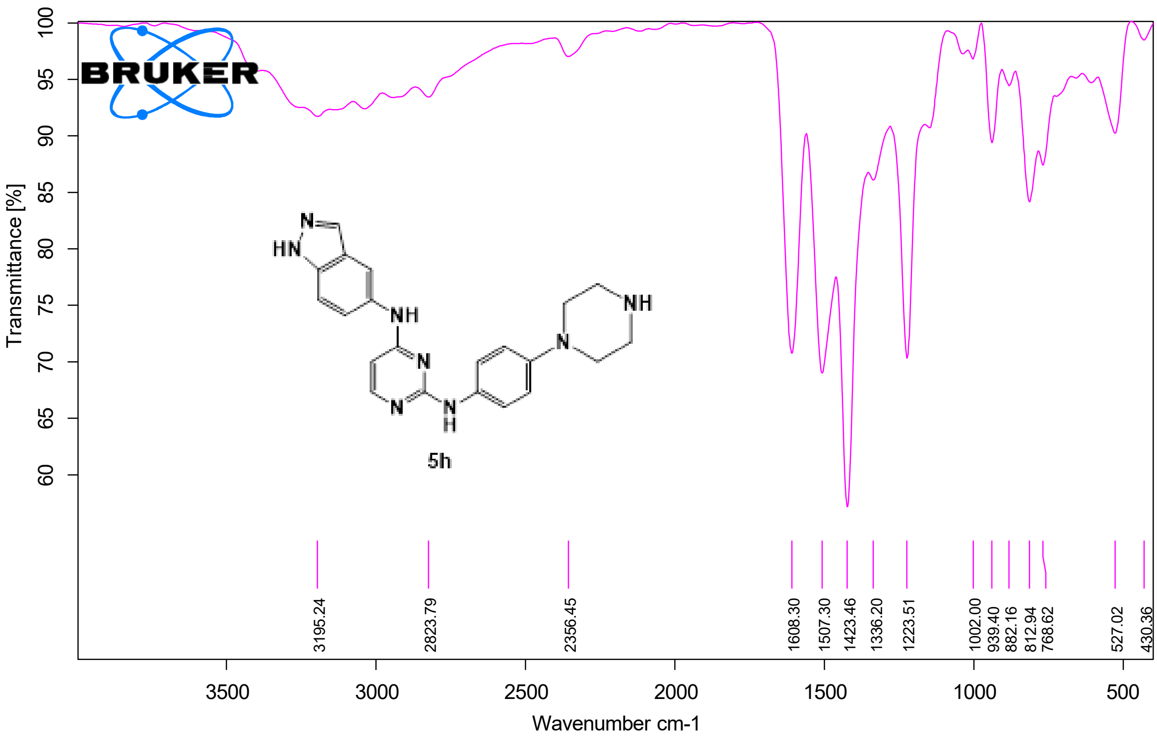

Figure S34: IR spectrum of compound **5h**

Supplementary Material

Indazol-Pyrimidine Hybrids: Design, Synthesis, and Antiproliferative Activity Against Human Cancer Cell Lines

Contents: Mass spectra

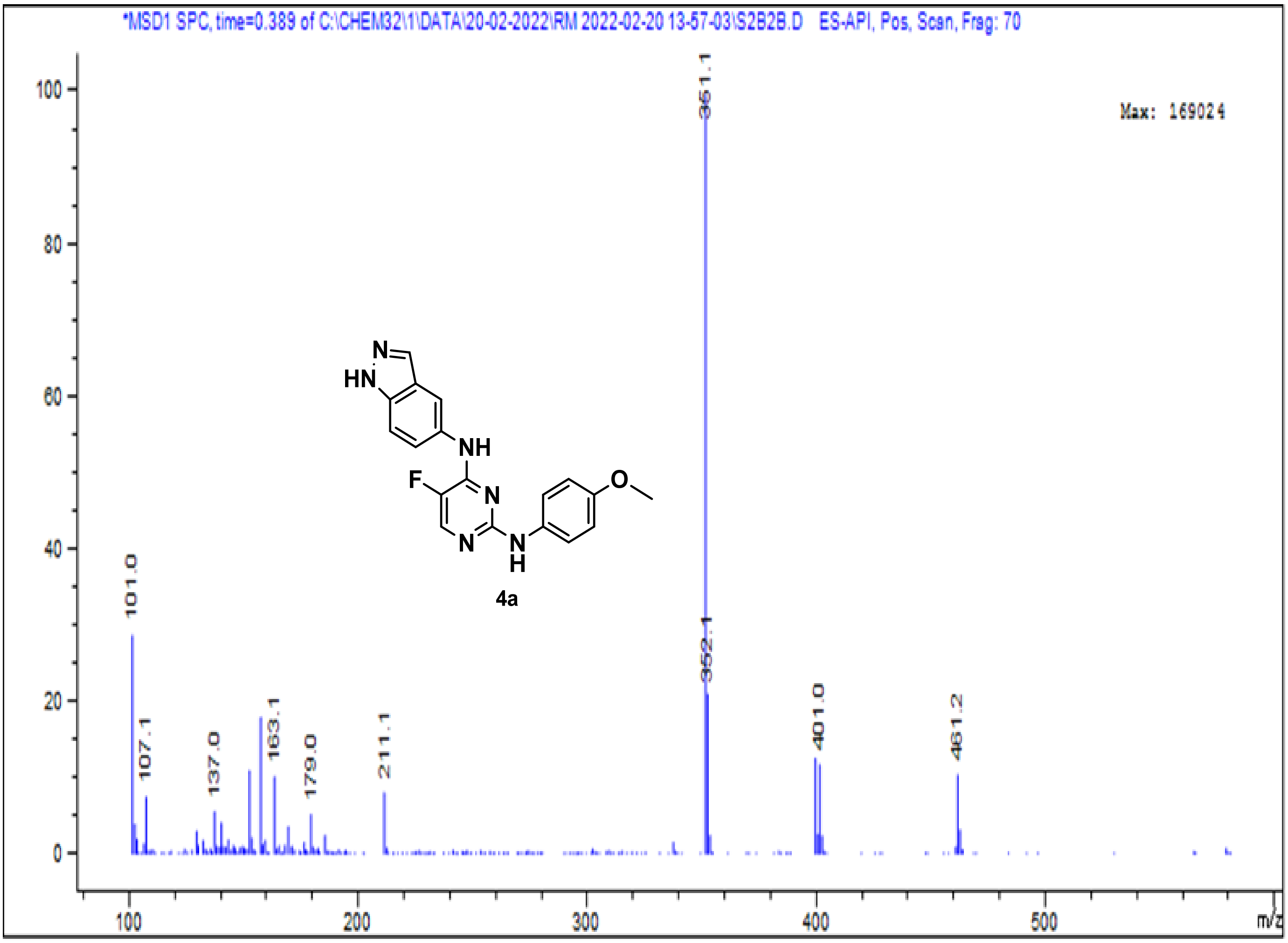

Figure S35: Mass spectrum of compound **4a**

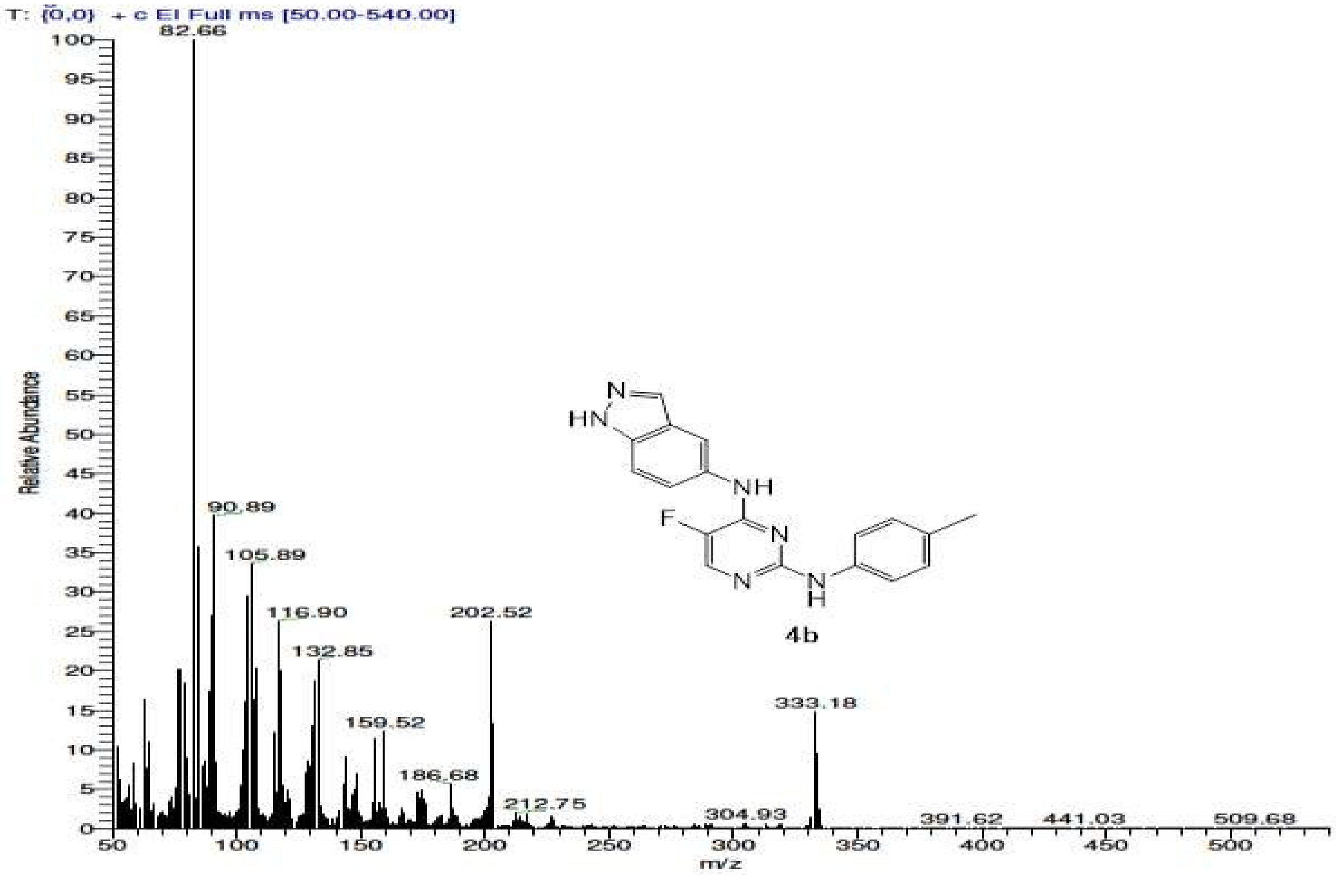

Figure S36: Mass spectrum of compound **4b**

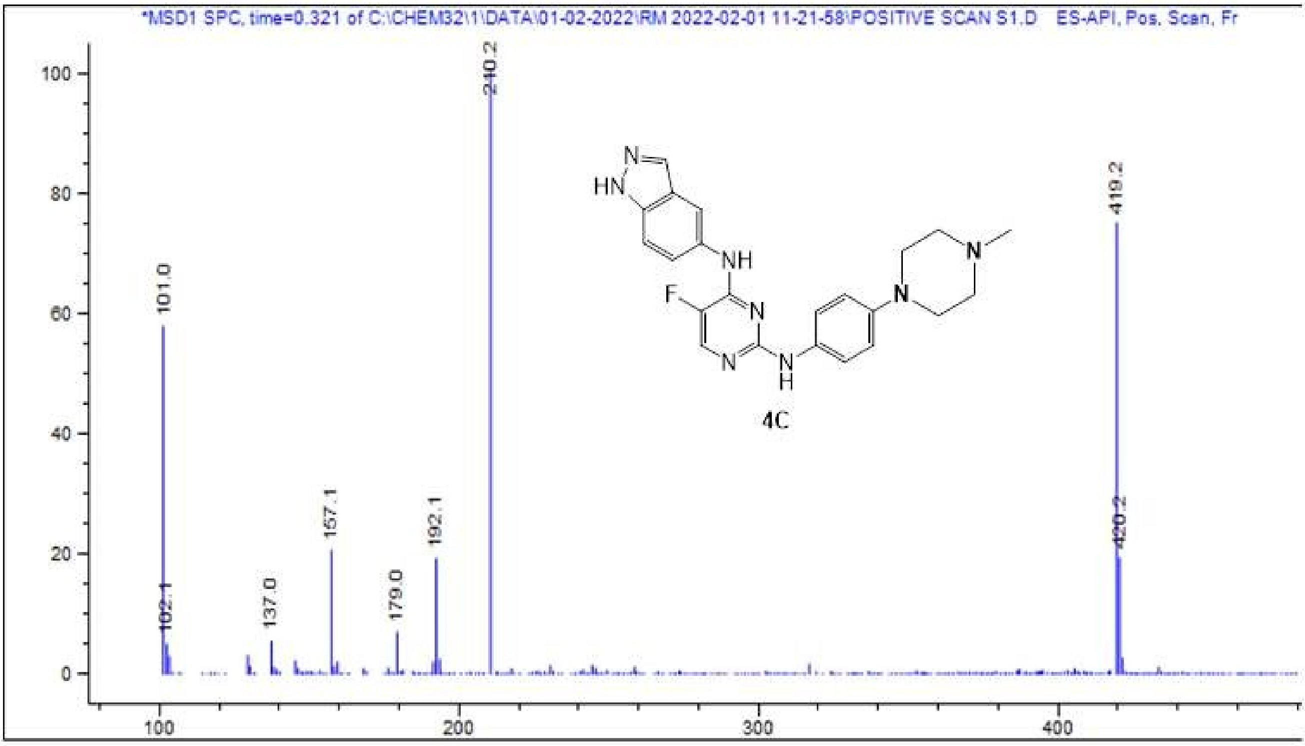

Figure S37: Mass spectrum of compound **4c**

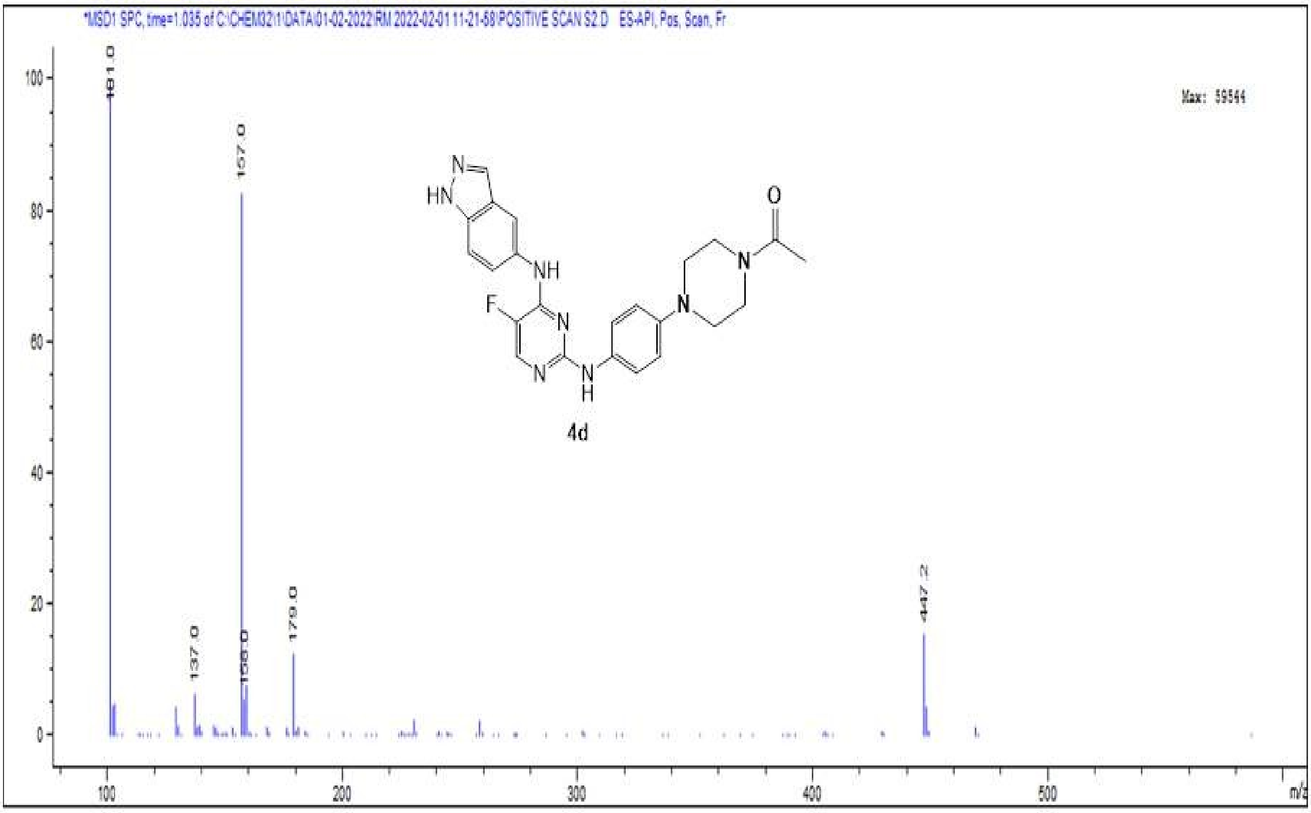

Figure S38: Mass spectrum of compound **4d**

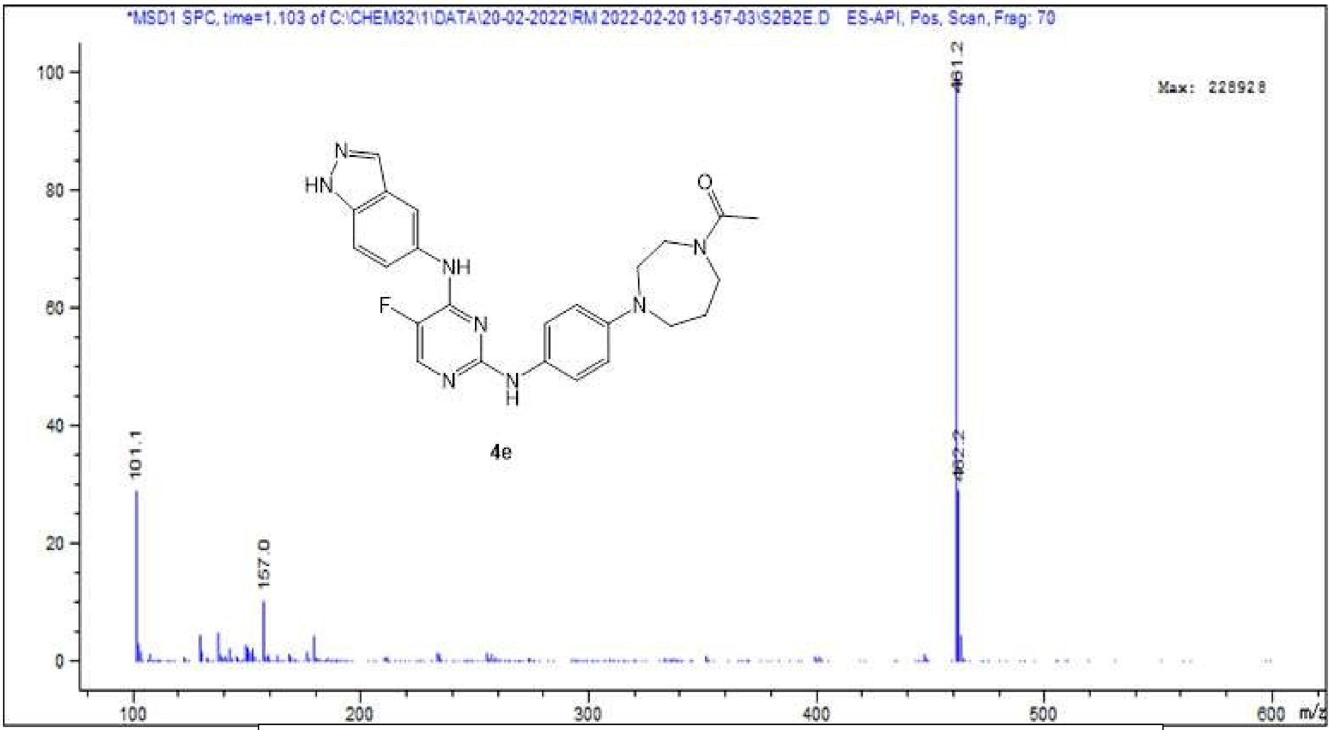

Figure S39: Mass spectrum of compound **4e**

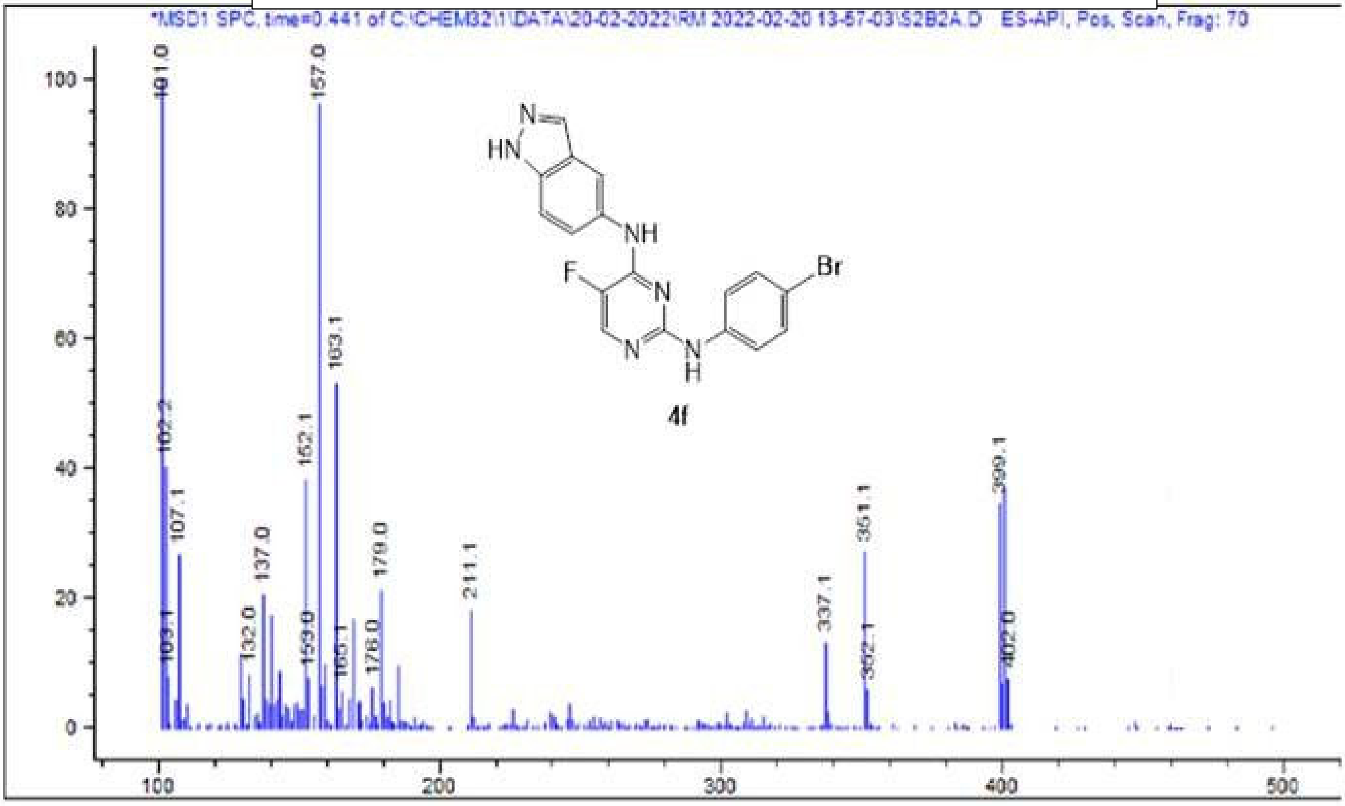

Figure S40: Mass spectrum of compound **4f**

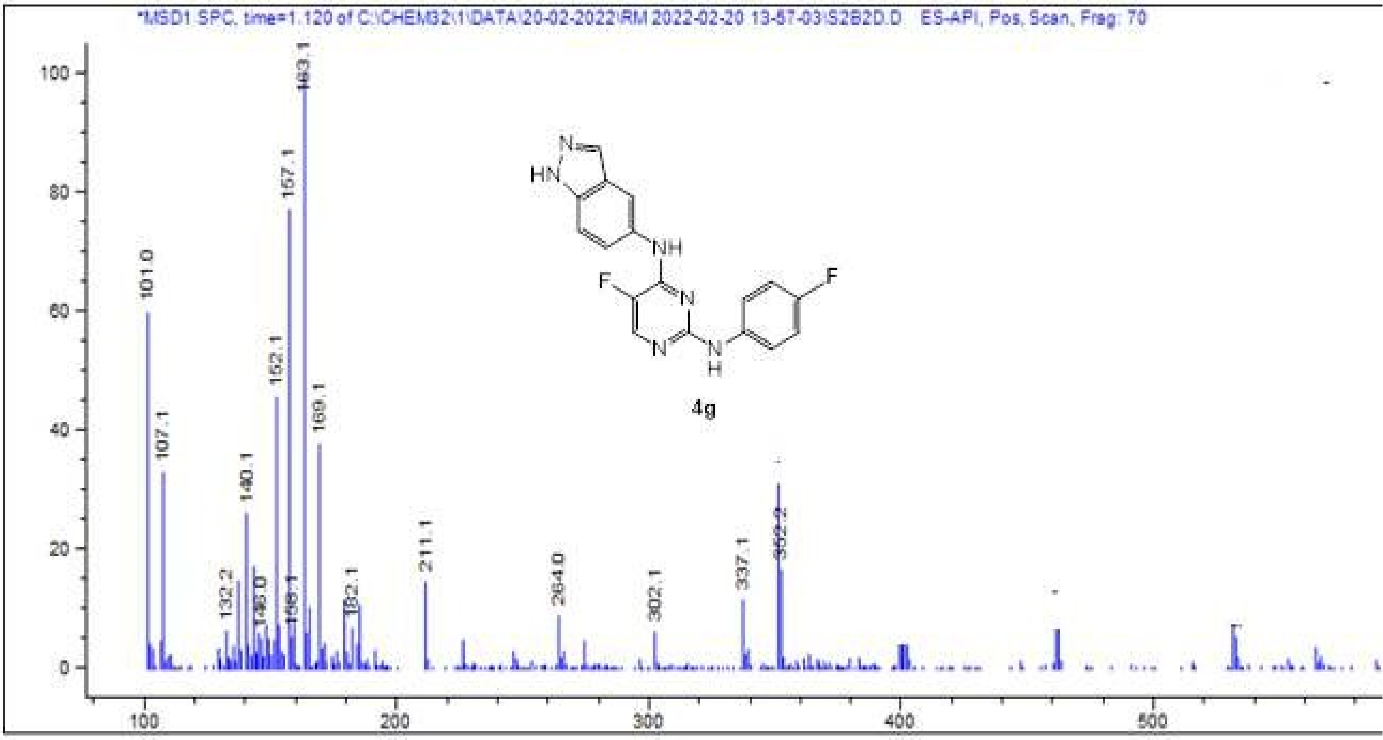

Figure S41: Mass spectrum of compound **4g**

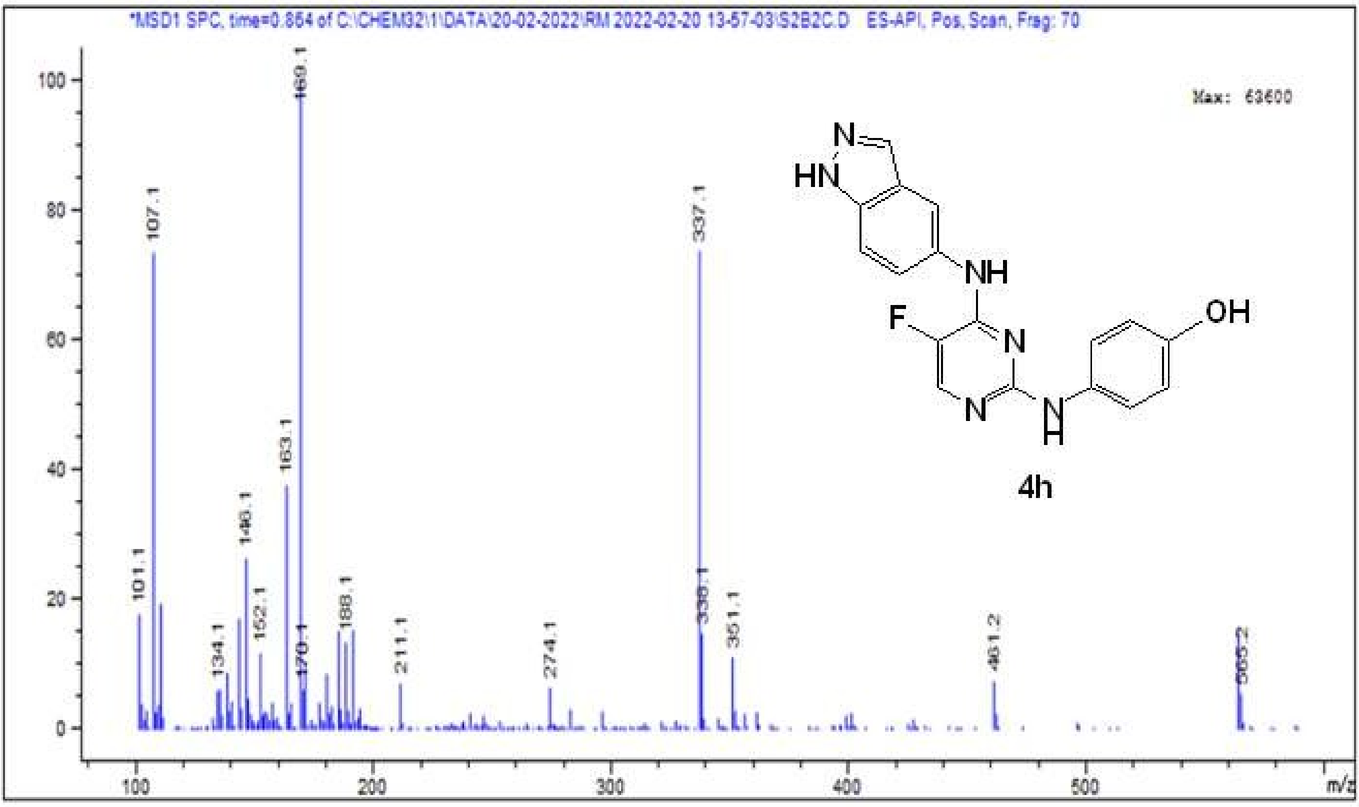

Figure S42: Mass spectrum of compound **4h**

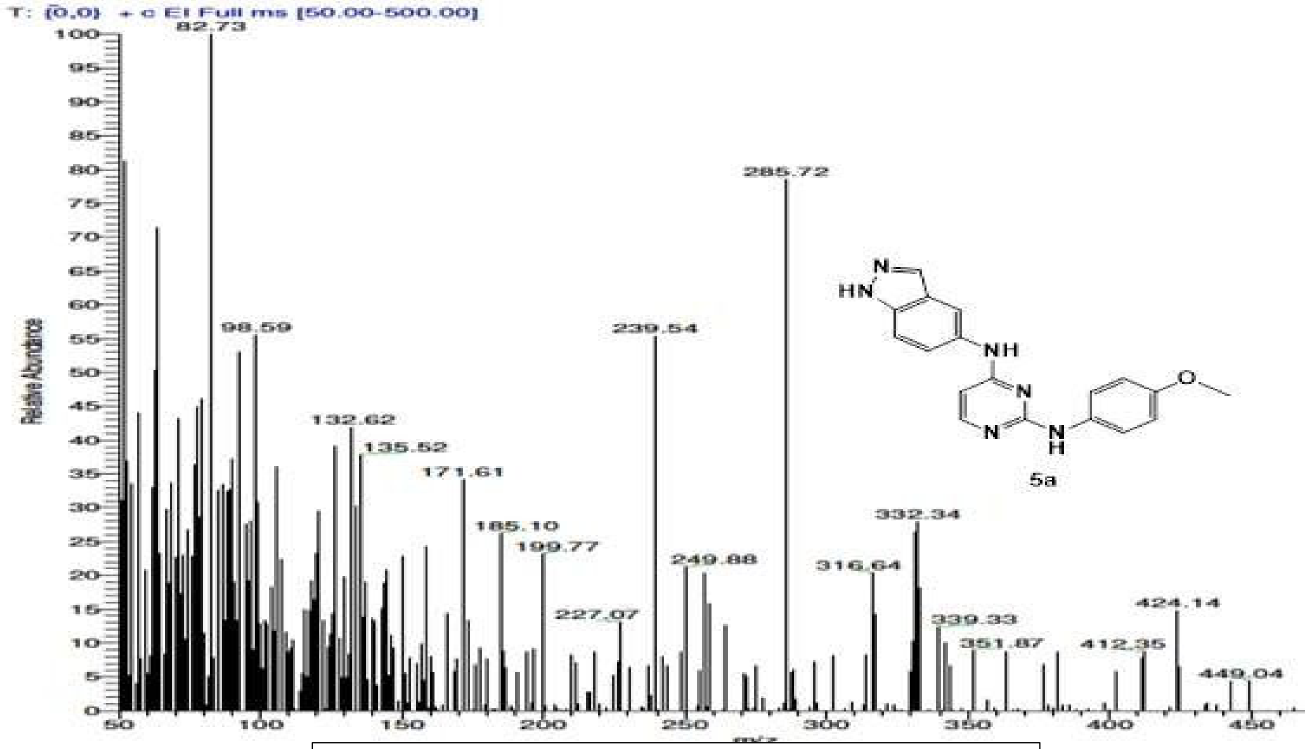

Figure S43: Mass spectrum of compound **5a**

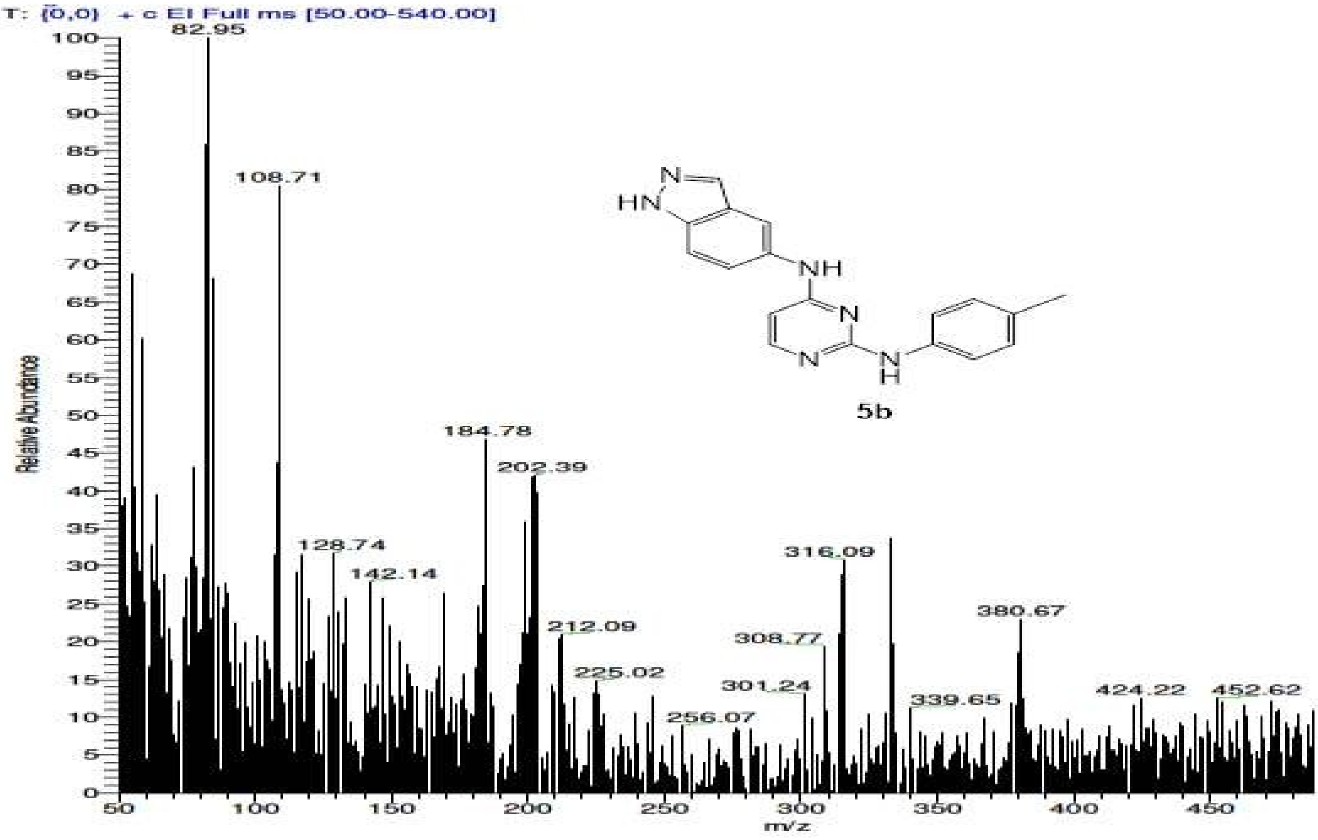

Figure S44: Mass spectrum of compound **5b**

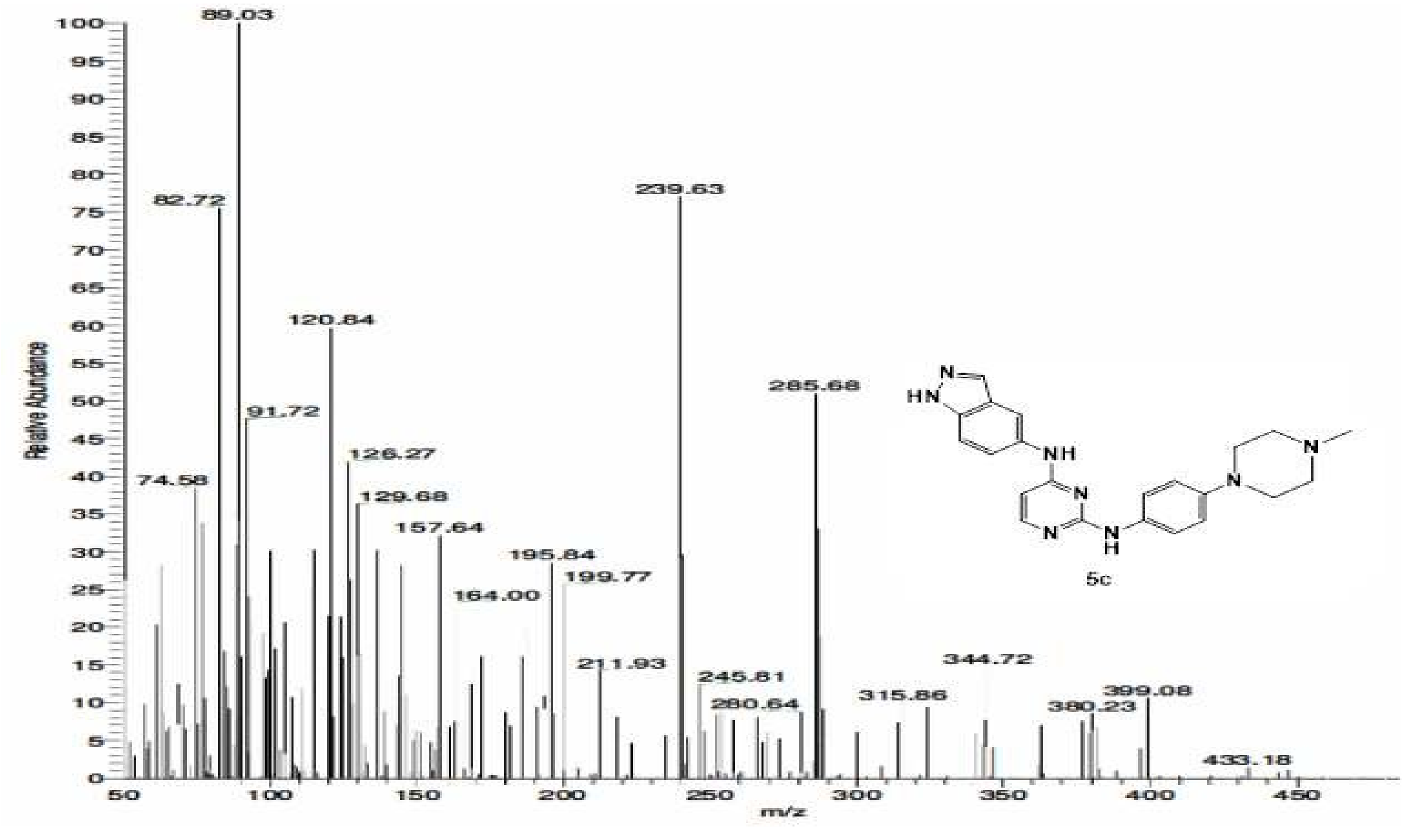

Figure S45: Mass spectrum of compound **5c**

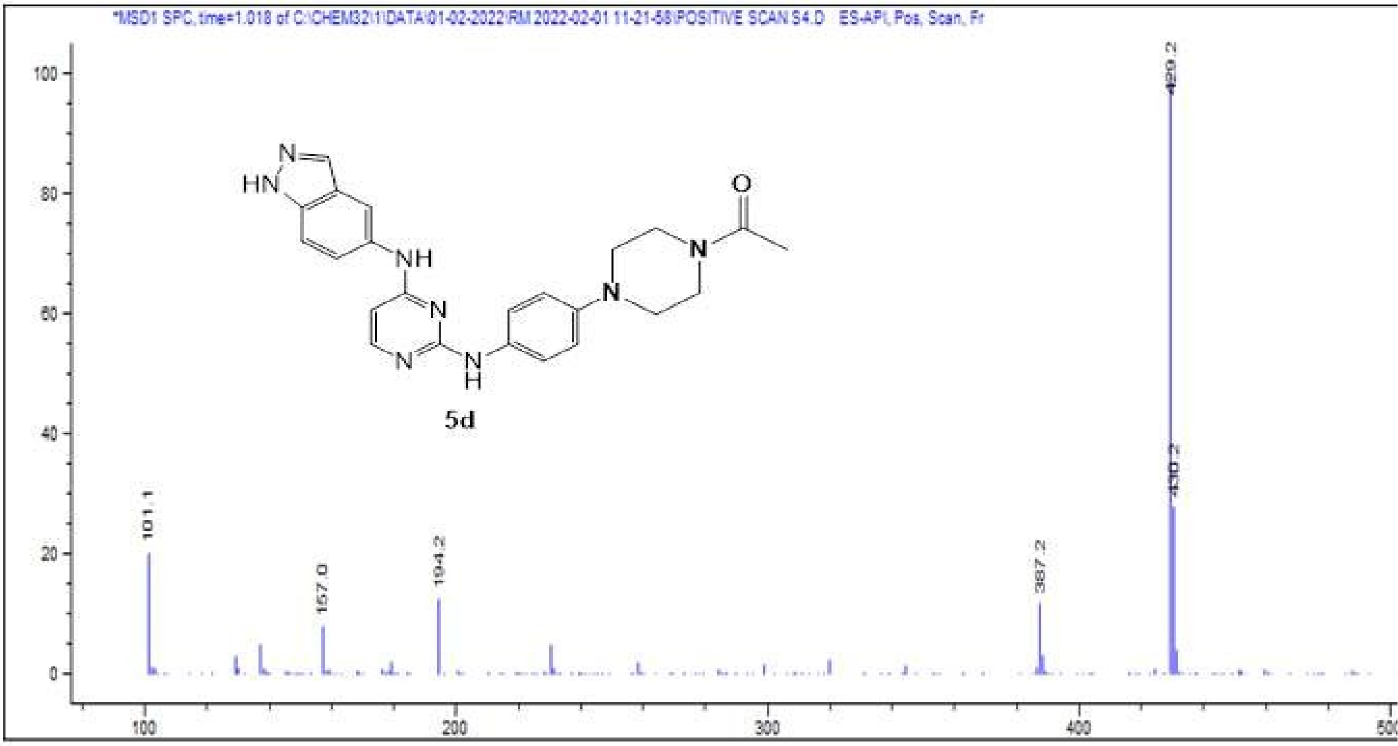

Figure S46: Mass spectrum of compound **5d**

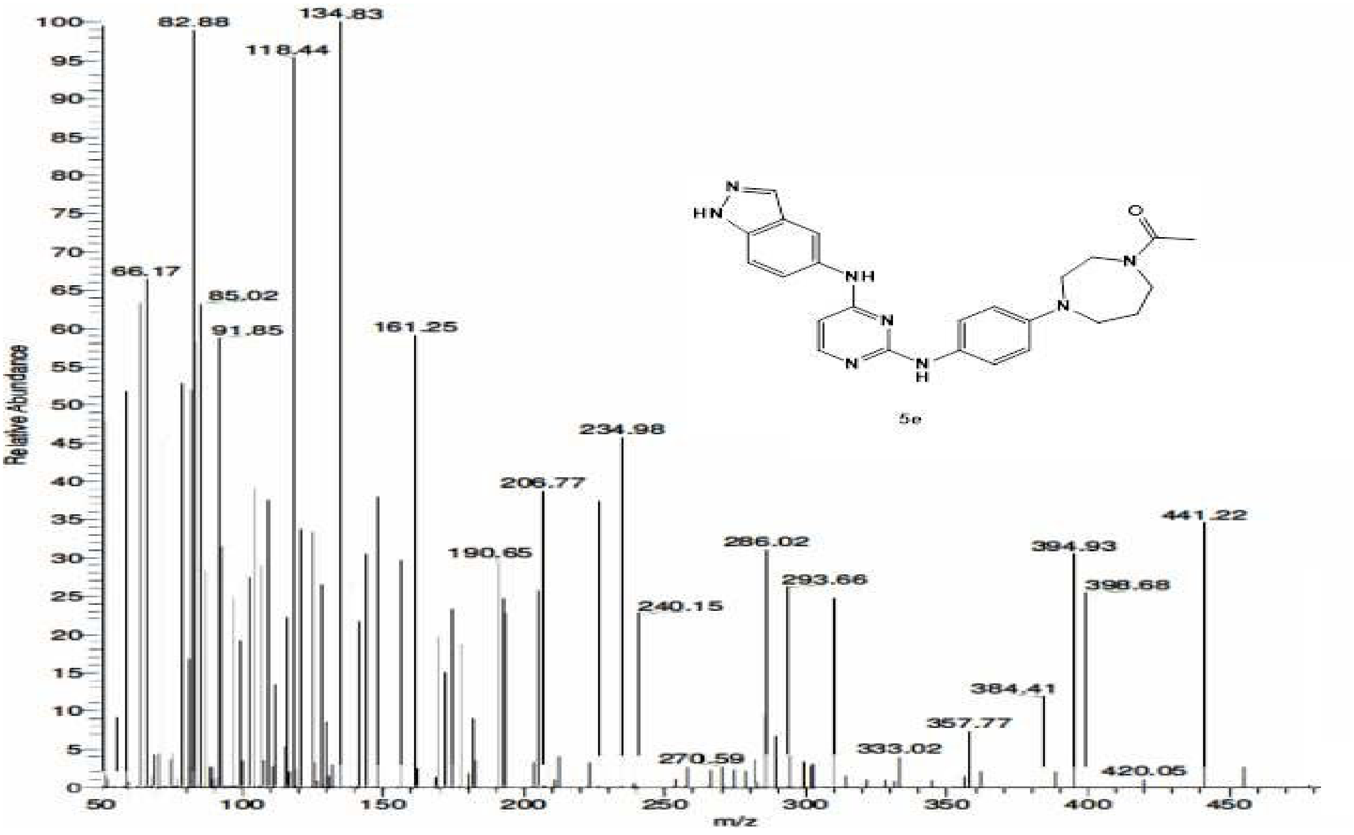

Figure S47: Mass spectrum of compound **5e**

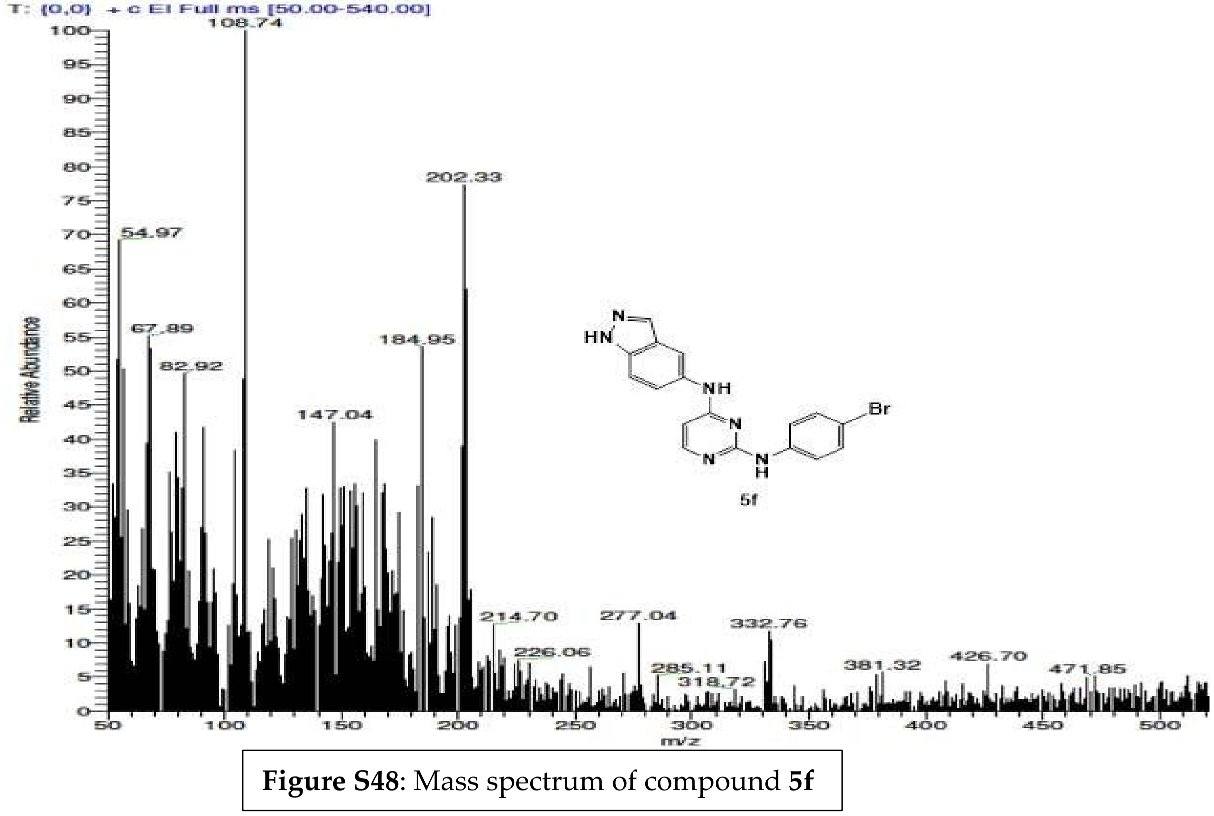

Figure S48: Mass spectrum of compound **5f**

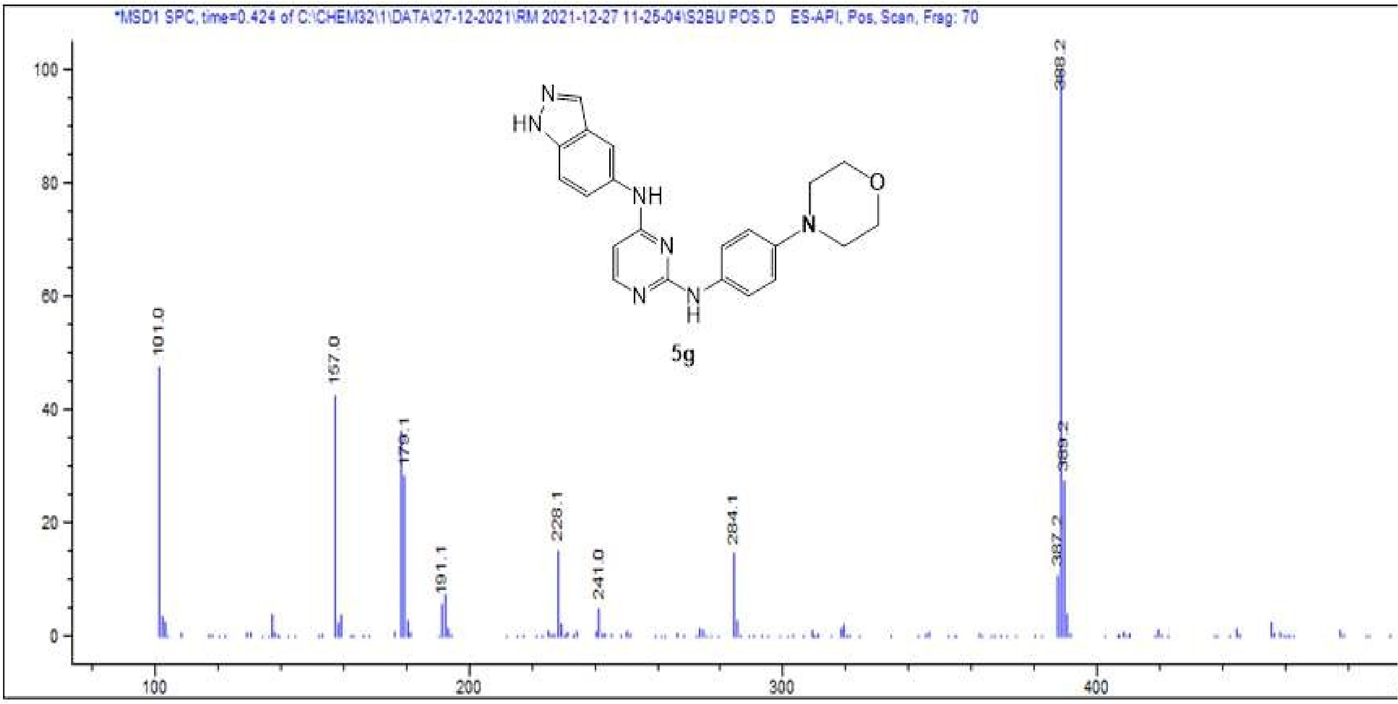

Figure S49: Mass spectrum of compound **5g**

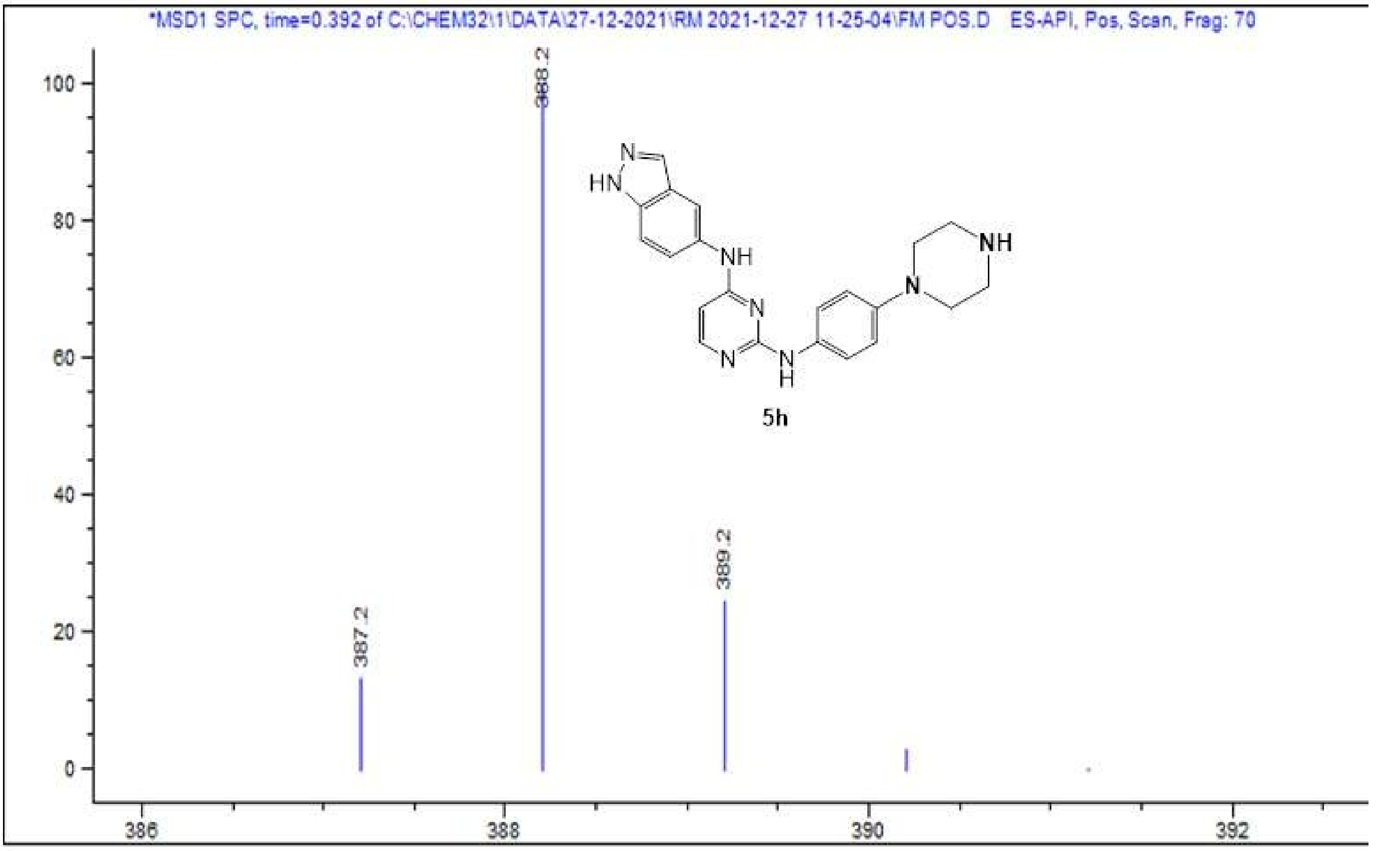

Figure S50: Mass spectrum of compound **5h**
